# New Sulfonate-Semicarbazone Hybrid Molecules: Synthesis, Theoretical Evaluations, Molecular Simulations, and Butyrylcholinesterase Inhibition Activity

**DOI:** 10.1021/acsomega.5c09763

**Published:** 2025-12-26

**Authors:** Bedriye Seda Kurşun-Aktar, Emine Elçin Oruç-Emre, Zafer Bulut, Emel Ekinci, Volkan Eyüpoğlu, Şevki Adem, Ayşegül Karaküçük-İyidoğan

**Affiliations:** † Department of Engineering Basic Sciences, Faculty of Engineering and Natural Sciences, 531771 Malatya Turgut Özal University, Battalgazi, Malatya 44210, Türkiye; ‡ Department of Chemistry, Faculty of Arts and Sciences, 37512 Gaziantep University, Gaziantep 27310, Türkiye; § 175171 Çankırı Karatekin University, Central Research Laboratory Application and Research Center, Çankırı 18100, Türkiye; ∥ Çankırı Karatekin University, Faculty of Science, Department of Chemistry, Çankırı 18100, Türkiye

## Abstract

In the present work, 18 novel semicarbazone-sulfonate hybrids were synthesized to evaluate their potential as butyrylcholinesterase (BChE) inhibitors. Among all compounds, 4-[(*E*)-(2-carbamoylhydrazinylidene)­methyl]­phenyl 2-(trifluoromethoxy)­benzene-1-sulfonate (**12**), 4-[(*E*)-(2-carbamoylhydrazinylidene)­methyl]­phenyl naphthalene-1-sulfonate (**17**), and 4-[(*E*)-(2-carbamoylhydrazinylidene)­methyl]­phenyl naphthalene-2-sulfonate (**18**) exhibited the most potent BChE inhibition, with IC_50_ values of 61.88, 77.02, and 93.67 μM, respectively, outperforming the reference drug pyridostigmine bromide (IC_50_: 130.04 μM). As shown by molecular docking studies, numerous interactions, including hydrogen bonds, π–π stacking, π–sulfur contacts, and halogen bonds, supported the high binding of compounds’ affinities to the BChE active site. Also, this study employed molecular dynamics (MD) simulations to assess the inhibitory potential of compounds **12**, **17**, and **18** against BChE. All ligands retained structural integrity during simulations. Compound **17** exhibited the highest conformational stability (minimal RMSD values) and formed robust interactions with critical binding site residues. Additionally, compound **18** demonstrated superior hydrogen bonding capacity, while compound **17** achieved the strongest binding affinity. Furthermore, *in silico* ADME predictions for the most active molecules showed good pharmacokinetic profiles and drug-likeness. Consequently, the results suggested that semicarbazone-sulfonate hybrid compounds **12**, **17**, and **18** were promising potential multifunctional agents targeting cholinergic dysfunction in Alzheimer’s disease (AD).

## Graphical abstract

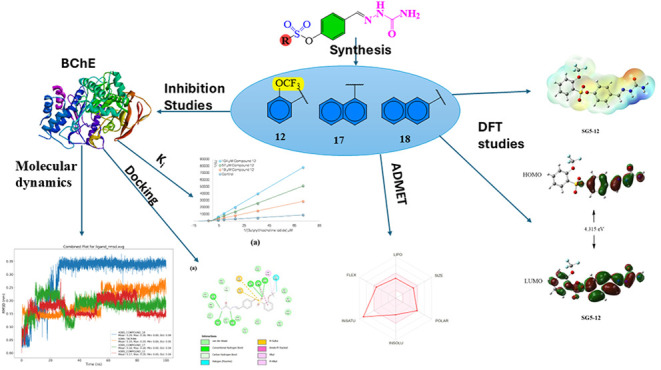

## 1 Introduction

The chronic neurodegenerative disease known as Alzheimer’s disease (AD) causes the brain’s neurons and synaptic connections to deteriorate gradually, which eventually impairs memory, reduces cognitive function, and changes behavior. It is also the primary cause of dementia, a general term for conditions characterized by severe cognitive decline that impairs daily functioning and self-sufficiency.^1–3^ Globally, AD affects more than 50 million individuals, a figure that is projected to nearly triple, reaching up to 150 million cases by 2050.^4^ AD advances through distinct stages, beginning with preclinical changes before progressing to mild, moderate, and eventually severe forms, with symptoms intensifying at each stage. Although its precise cause is still not fully understood, its development is thought to result from a complex interplay of genetic predisposition, lifestyle choices, and environmental influences, with advancing age being a predominant risk factor.^5,6^ On a pathological level, AD is marked by the accumulation of amyloid-β plaques, the formation of neurofibrillary tangles, and the deterioration of neuronal connections, all of which contribute to cognitive decline.^7^ While a definitive cure remains unavailable, current interventions, including cholinesterase inhibitors and memantine, lifestyle modifications, and comprehensive supportive care, can help alleviate symptoms and improve patients’ quality of life.^5,6,8^ The loss of cholinergic neurons leads to a significant decline in acetylcholine (ACh), a neurotransmitter vital for cognition. In AD, the enzymes acetylcholinesterase (AChE) and butyrylcholinesterase (BChE) play crucial roles in both its progression and treatment.^9^ AChE inhibitors such as donepezil, rivastigmine, and galantamine help increase ACh levels, thereby improving cognitive function in early to moderate AD. As the disease advances, BChE activity rises, further contributing to ACh depletion.^10–12^ Thus, inhibiting BChE to enhance the level of ACh has gained importance as an alternative therapeutic target for managing symptoms and slowing AD progression.^13^ Also, BChE has been reported to slow the formation of Aβ fibrils by acting as a molecular chaperone, stabilizing soluble Aβ groups.^12^ From this point of view, it is thought that compounds that inhibit BChE by binding to both the catalytic active site (CAS) and the PAS region can prevent Aβ-associated toxicity while simultaneously reducing ACh hydrolysis. Only rivastigmine is the most selective for BChE and is particularly suitable for moderate to severe AD, among FDA-approved anticholinesterase drugs.^14^ However, in addition to side effects such as nausea, vomiting, diarrhea, stomach pain, anorexia, and weight loss, 17–35% of patients experienced delusions, hallucinations, agitation and aggression, disinhibition, irritability and lability, and abnormal motor behavior.^15^ Consequently, the exploration of potential BChE inhibitor candidates for the management of AD has become imperative.

In light of this knowledge, semicarbazones are one of the scaffolds that could be used as drug candidates due to their structural modifiability and diverse biological activities, including anticonvulsant, anticancer, antimicrobial, antidiabetic, and anticholinesterase properties.^16–20^ They are also imine derivatives that can be easily and simply obtained by the condensation reaction between a ketone or aldehyde and a semicarbazide.^21^ There is evidence that semicarbazones can interact more readily with cholinesterases by mimicking the structure of the substrate, thanks to the presence of the imine group, which facilitates hydrogen bonding and electrostatic interactions with critical residues in the active sites of cholinesterases, and the carbonyl group, which polarizes the ligand and makes it more electrophilic.^16,22^ Although there are some studies proving that semicarbazone derivatives are effective on AChE and BChE, the therapeutic potential of semicarbazones in the treatment of AD has not been fully appreciated.^23–26^ Tripathi et al. reported that among semicarbazone derivatives derived from 2-amino-5-nitrothiazole, a compound bearing a chloro group in the *para* position was the lead compound with an IC_50_: 0.024 μM against BChE.^27^ Also, in another study, a series of carbazole-based semicarbazones and hydrazones were designed, synthesized, and assessed for their anticholinesterase inhibitory activity.^21^ Neto et al. synthesized a thiosemicarbazone and a semicarbazone containing an acridine group and evaluated their cholinesterase inhibitory potential. They also reported that the compounds were weaker than tacrine against BChE but less toxic *in silico* ^20^ and noted that semicarbazones likely have a more rigid or specific structure, allowing for stronger and more stable interactions with ChEs.^12^ However, these limited studies suggest that semicarbazones may have significant potential in the development of more selective and effective drug candidates for the treatment of AD. Therefore, a new series of semicarbazone derivatives was designed in this study to contribute to a more comprehensive investigation of semicarbazones, as they may have significant potential as effective BChE inhibitor candidates for anti-Alzheimer’s agents. On the other hand, due to their high lipophilicity, sulfonates are molecules with excellent binding capacity to the active sites of target enzymes, owing to their favorable physicochemical properties. Moreover, arylsulfonate derivatives are one of the most remarkable pharmacophore groups recently and are promising, especially in terms of their inhibitory activity against butyrylcholinesterase.^28,29^

In this study, to identify new hybrid scaffolds and continue our studies on cholinesterase inhibitors, we designed a series of hybrid compounds consisting of 18 semicarbazone-sulfonate hybrid molecules to investigate the effect of substituents present on the aryl ring attached to the sulfonate structure and ring size on BChE inhibitory activity. These new hybrid compounds were designed and synthesized by a molecular hybridization approach (**1–18**), which is a drug design method based on combining pharmacophore groups to discover new drug candidates. Furthermore, molecular docking studies were conducted to evaluate the inhibition mechanisms and determine the stability of the ligand–protein complexes for the most active compounds. The physicochemical properties of the compounds and their binding affinities to the target protein were investigated by using molecular docking, molecular dynamics simulations, and quantum chemical calculations. Finally, *in silico* ADME (Absorption, Distribution, Metabolism, and Excretion) profiling was performed for all compounds to explore their drug-likeness.

## 2 Results and Discussion

### 2.1 Chemistry

In this study, 18 novel (*E*)-4-[(2-carbamoylhydrazinylidene)­methyl]­phenyl-substituted benzenesulfonate derivatives (**1–18**) were designed and synthesized for the first time, as illustrated in Scheme 1. *p*-Hydroxybenzaldehyde was reacted with sulfonyl chloride derivatives in the presence of triethylamine to afford arylsulfonyloxybenzaldehyde intermediates (**A1–A18**). Subsequent reactions of compounds **A1–A18** with semicarbazide yielded the corresponding semicarbazones (**1–18**) in excellent yields (93–98%). The substituents of the synthesized compounds are listed in Scheme 1. The chemical structures of the new derivatives were confirmed by spectroscopic techniques, including FT-IR, ^1^H NMR, and ^13^C NMR analyses.

**Scheme 1:**
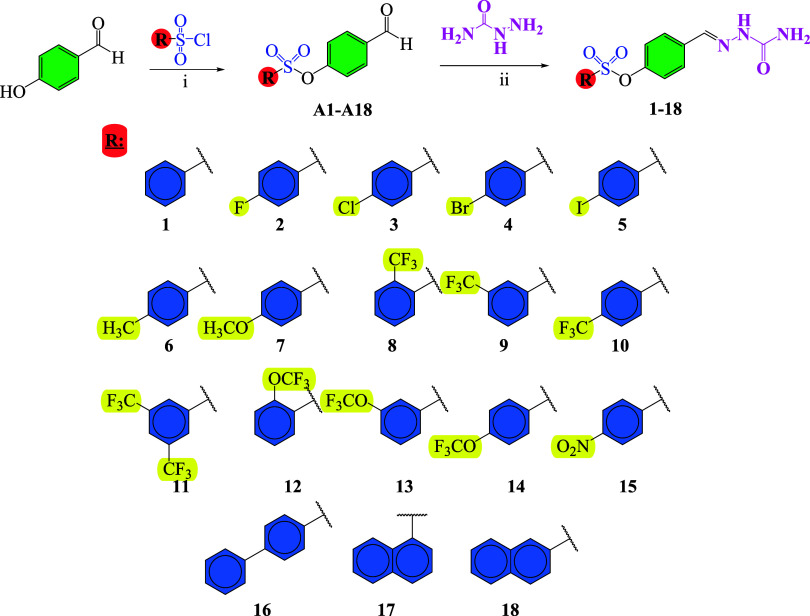
Synthetic Pathway of Semicarbazone-Sulfonate Hybrid Molecules

In the FT-IR spectra of compounds (**1–18**), it was determined that the C=N stretching band, which is one of the characteristic absorptions of the semicarbazone moiety, appeared in the range of 1567–1596 cm^–1^ and the N–H stretching vibrations were observed in the range of 3545–3145 cm^–1^. Also, the asymmetric and symmetric stretching bands of SO_2_ were determined at 1350–1384 and 1133–1199 cm^–1^, respectively. In the ^1^H NMR spectra, evidence for the formation of semicarbazones was provided by the appearance of N–H proton signals between δ 10.36 and 10.30 ppm as a singlet, together with the – NH_2_ protons appearing as a doublet (2H) between δ 6.56 and 6.50 ppm. In addition, the −CH proton of the azomethine (C=N) group was observed as a singlet at δ 7.80–7.71 ppm. In the ^13^C NMR spectra, the azomethine (C=N) carbon and the carbonyl (C=O) carbon, which are among the most significant signals of hydrazone-type compounds, resonated in the range of δ 166.92–149.05 ppm. Signals of aromatic carbons were also clearly observed in their expected regions. All spectroscopic data obtained for the characterization of semicarbazone-sulfonate derivatives (**1–18**) were consistent with the previously reported values.^30–36^

### 2.2 DFT

#### 2.2.1 Frontier Molecular Orbitals

In quantum chemistry, frontier molecular orbitals (FMOs)—namely, the highest occupied molecular orbital (HOMO) and the lowest unoccupied molecular orbital (LUMO)—along with their energy difference (Δ*E* = *E*_HOMO_ – *E*_LUMO_), are fundamental indicators of a molecule’s electronic behavior. These parameters provide important information about the kinetic stability, biological potential, and reactivity of a molecule.^37^ The FMO profiles and associated HOMO–LUMO energy gaps of compounds **12**, **17**, and **18** are shown in Figure 1.

**Figure 1:**
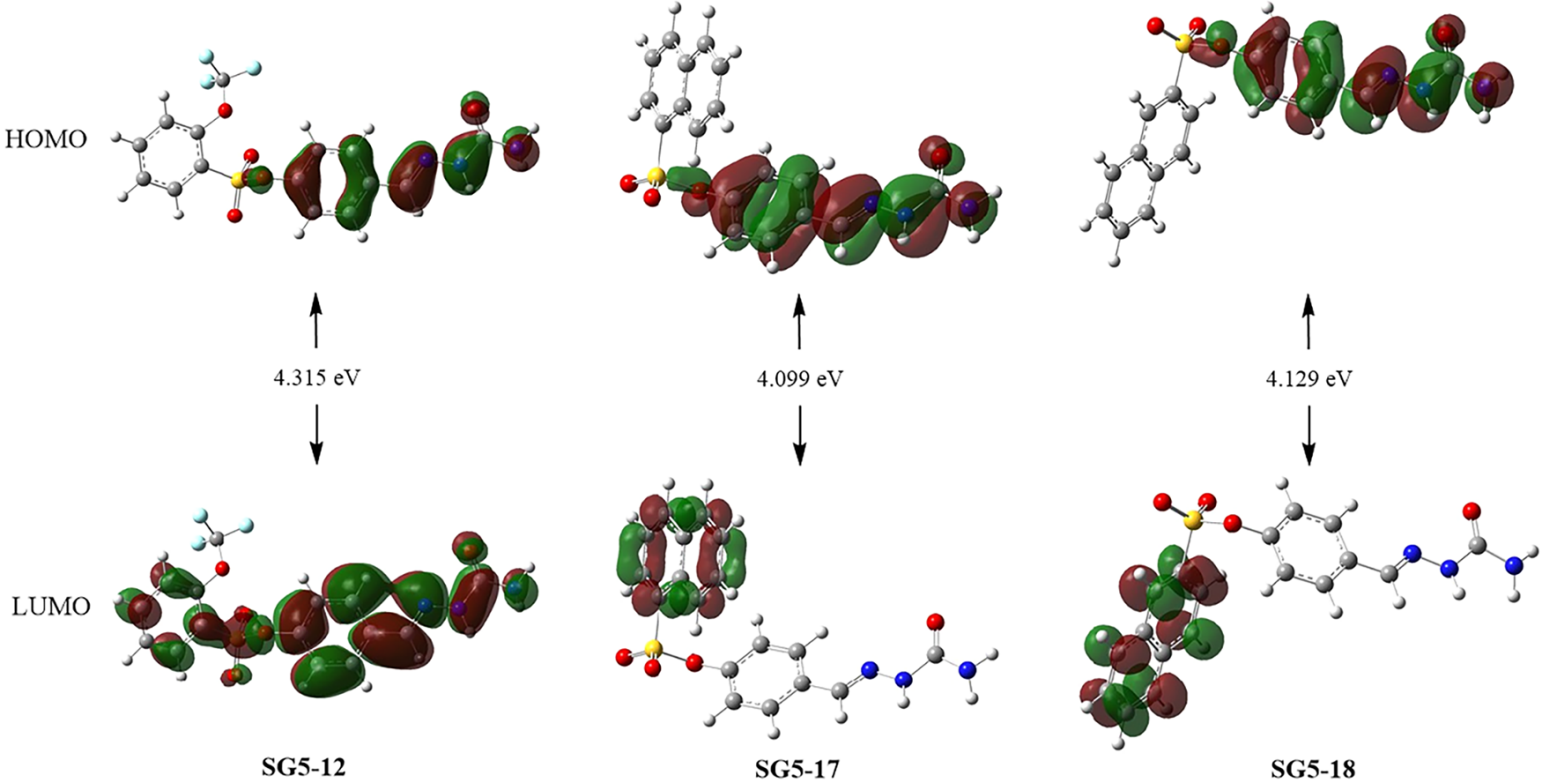
HOMO and LUMO plots for **12, 17**, and **18** at the B3LYP/6–311++G­(d,p) level.

The evaluation of HOMO and LUMO energy levels in compounds facilitates the determination of several global reactivity parameters, including the ionization energy (I), electron affinity (A), chemical hardness (η), chemical softness (σ), electronegativity (χ), chemical potential (μ), and electrophilicity index (ω). These descriptors of quantum chemistry are essential for forecasting the behavior of molecules in various chemical environments, providing information on their stability, reactivity, and possible interactions with biological targets.^38^

#### 2.2.2 Global Reactivity Parameters

The global reactivity descriptors are presented in Table 1, which provides important information about the electrical properties of compounds **12**, **17**, and **18**. A chemical with a lower *E*_LUMO_ value indicates a better ability to accept electrons, while a compound with a higher *E*_HOMO_ value generally has a greater tendency to donate electrons. Among the compounds, **18** stood out with its remarkable capacity to donate electrons, with the highest *E*_HOMO_ level of −6.368 eV. On the other hand, compound **12** had the lowest *E*_LUMO_ value of −2.047 eV in the gas phase, indicating a strong capacity to accept electrons.

**Table 1:** Global Reactivity Parameters (in eV) for **12, 17**, and **18**

compound	*E*_HOMO_	*E*_LUMO_	Δ*E*	IP	EA	μ	η	σ	χ	ω
**12**	–6.362	–2.047	4.315	6.362	2.047	–4.204	2.158	0.463	4.204	4.096
**17**	–6.351	–2.252	4.099	6.351	2.252	–4.302	2.049	0.488	4.302	4.515
**18**	–6.368	–2.239	4.129	6.368	2.239	–4.303	2.064	0.484	4.303	4.485

In conclusion, the analysis of global reactivity descriptors revealed notable differences in the electronic properties of the studied compounds. While compound **12**, which had the lowest electronegativity and electrophilicity index, mostly functions as an electron donor, compound **18**, which had the highest electronegativity, was a notable strong electron acceptor. On the other hand, compound **17** exhibited the highest electrophilicity, indicating its enhanced electrophilic character. Notably, increased biological activity was typically linked to reduced electrophilicity.^38^ These findings suggested that the electron-donating capacity and reduced electrophilicity could be advantageous features for designing biologically active molecules.

#### 2.2.3 Molecular Electrostatic Potential (MEP) Maps

The molecular electrostatic potential (MEP) maps of compounds **12**, **17**, and **18** are given in Figure 2. These maps provided visual insights into the charge distribution over the molecular surfaces, highlighting regions prone to electrophilic and nucleophilic interactions. The color gradient ranged from red (electron-rich, negative potential) to blue (electron-deficient, positive potential). Those with a high electron density were shown on these maps as red, and those with a low electron density were shown as blue.^39,40^ In compound **12**, intense red regions were observed around the carbonyl and sulfonyl oxygen atoms, indicating a high electron density and potential nucleophilic attack sites. The blue areas near hydrogen atoms suggested regions susceptible to electrophilic attack. Compounds **17** and **18** showed a more balanced distribution with prominent red regions at the sulfonyl moiety and medium blue regions near the amine groups, indicating the potential for both electrophilic and nucleophilic interactions. Overall, the MEP profiles indicated that differences in electrostatic potential distribution could influence the binding behavior and biological activity of these molecules.

**Figure 2:**
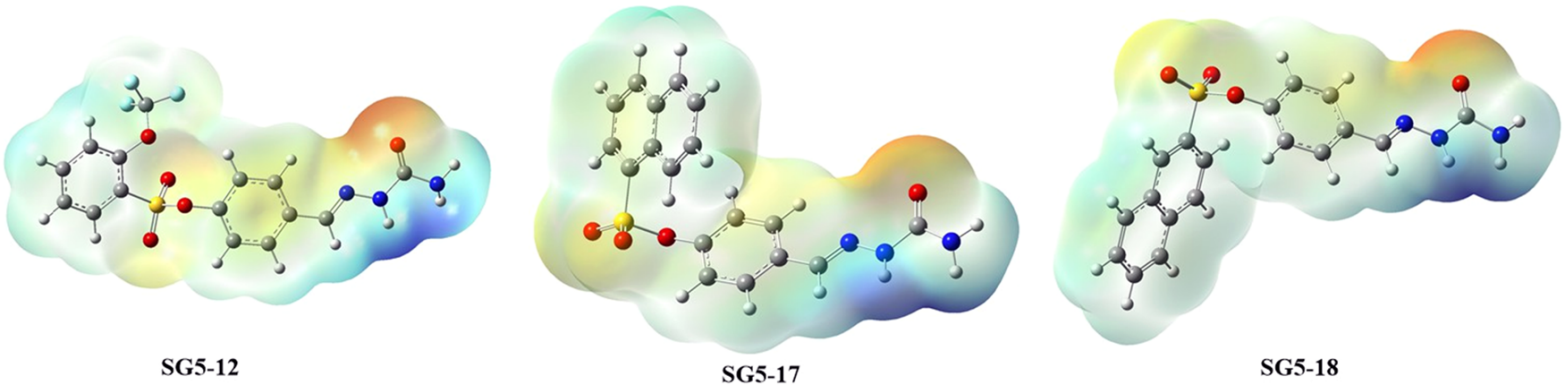
MEP maps of compounds at the B3LYP/cc-pVDZ level.

### 2.3 *In Vitro* Cholinesterase Inhibition Studies

As a result of *in vitro* inhibition studies of the BChE enzyme, IC_50_ values of semicarbazone-sulfonate hybrid compounds were found in the range of 61.88–346.57 μM (Table 2). The IC_50_ value of pyridostigmine bromide used as a reference inhibitor was 130.04 μM, and compounds **11**, **12**, **13**, **14**, **16**, **17**, and **18** remained below this value and showed a stronger inhibitory effect.

**Table 2:** Inhibitory Effects of Semicarbazone-Sulfonate Derivatives on BChE

Comp.	BChE IC_50_ (μM)	Comp.	BChE IC_50_ (μM)
**1**	139.466	**10**	138.629
**2**	161.197	**11**	113.631
**3**	141.459	**12**	61.888
**4**	176.823	**13**	115.525
**5**	173.287	**14**	129.560
**6**	177.730	**15**	346.574
**7**	136.178	**16**	111.798
**8**	172.424	**17**	77.016
**9**	147.165	**18**	93.669
**Pyridostigmine bromide**	**130.04**		

The butyrylcholinesterase (BChE) inhibitory activities of the compounds in the series show marked differences depending on the electronic, steric, and hydrophobic properties of the *R* groups on the aromatic ring in the structure. The IC_50_ values range from 61.88 to 346.57 μM.

The highest inhibitory activity was observed in compound **12** (R = OCF_3_, IC_50_ = 61.88 μM). This result may be due to the strong electron-withdrawing and polar trifluoromethoxy (−OCF_3_) group, providing a balanced combination of hydrophobic and polar interactions in the active site of BChE. Similarly, the relatively high activities of compounds **17** (R = naphthyl, IC_50_ = 77.02 μM) and **18** (R = 2-naphthyl, IC_50_ = 93.67 μM) may be attributed to the potential of their broad aromatic surfaces to form π–π stacking interactions within the enzyme’s hydrophobic pocket. This is consistent with the large hydrophobic active site of BChE.

In contrast, compound **15** (R = NO_2_, IC_50_ = 346.57 μM) exhibits the lowest inhibitory activity. The strong electron-withdrawing character and steric bulk of the nitro group likely impede access to the active site or a suitable binding conformation. Furthermore, the nonhydrophobic nature of the NO_2_ group may result in weak interactions with the apolar residues in the enzyme’s active site.

Halogen derivatives (*e.g.*, 1–4; F, Cl, Br, and I) generally exhibited moderate activity (IC_50_ ≈ 130–175 μM). Differences in size and electronegativity among the halogens did not cause significant variation in activity, suggesting that the interaction is primarily based on hydrophobic contacts. Similarly, compounds containing CF_3_ (*e.g.*, 8–11, 13) showed moderate activity; although these groups are electron-withdrawing, their high steric volume may have limited their complete integration into the active site.

Overall, it can be concluded that electron-withdrawing substituents that do not create excessive steric hindrance (*e.g.*, −OCF_3_) or groups providing a large aromatic surface (*e.g.*, naphthyl derivatives) are more advantageous for BChE inhibition. This result is consistent with the literature information that BChE has a larger active site compared to that of AChE and can tolerate large aromatic systems.

Overall, a strong correlation between the BChE inhibition activity and structural characteristics was found. These findings implied that compounds’ structural characteristics, particularly those of **12**, **17**, and **18**, can be assessed as pharmacophores and optimized by further molecular modeling.

### 2.4 Enzyme Kinetic Studies

Enzyme kinetic studies were performed on butyrylcholinesterase (BChE) using the most potent inhibitors, compounds **12**, **17**, and **18**, to elucidate their inhibition mechanisms. The enzymatic activity of BChE was evaluated at various concentrations of the substrate butyrylthiocholine iodide (BTChI) in the presence of fixed concentrations of each inhibitor. The resulting data were analyzed by Lineweaver–Burk double reciprocal plots to determine kinetic parameters such as *K*_m_, *V*_max_, and the inhibition type (Figure 3).

**Figure 3:**
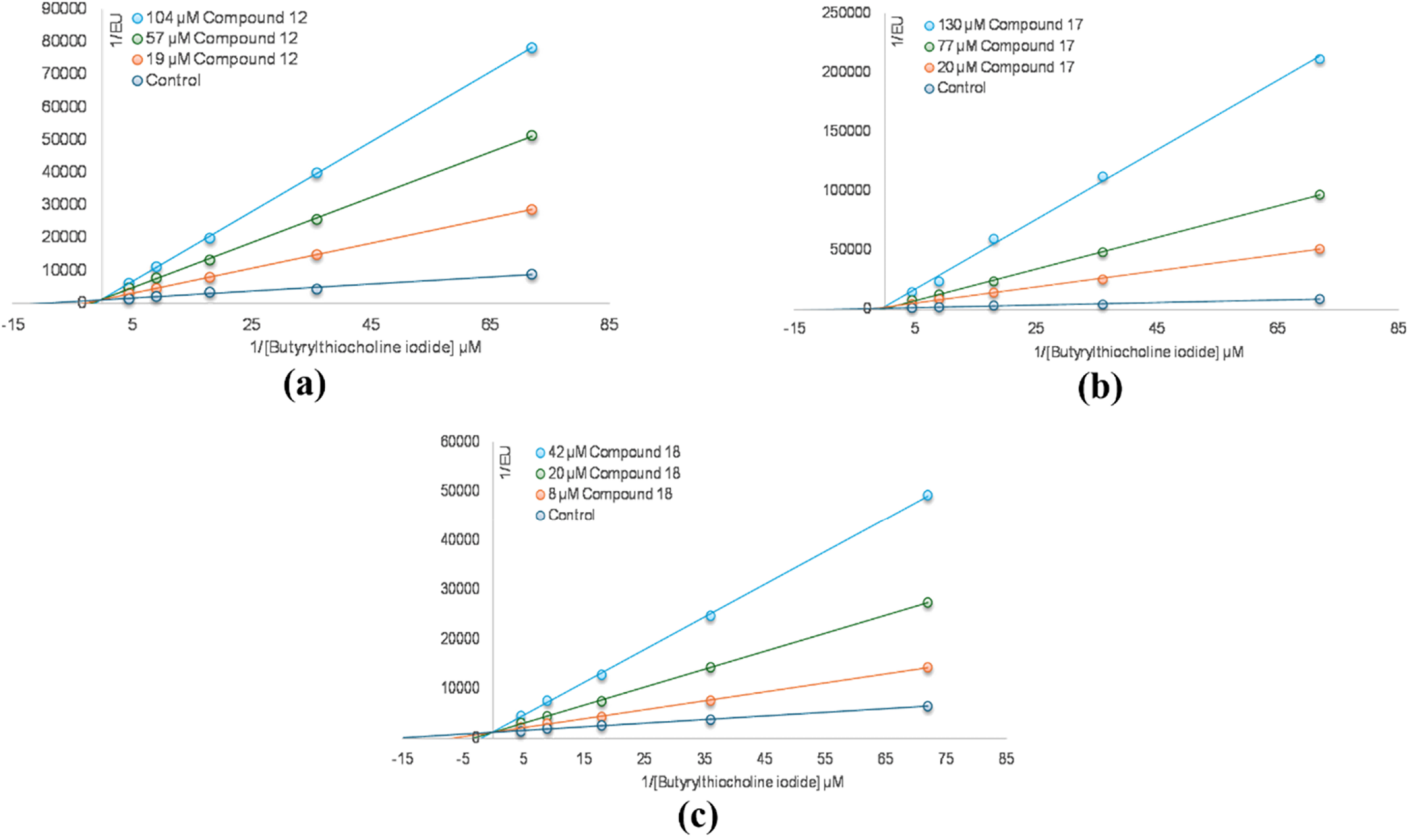
IC_50_ graph, Lineweaver–Burke plot of the inhibition kinetics of BChE for **12** (a), **17** (b), and **18** (c).

The Lineweaver–Burk plots demonstrated that for all tested compounds, the lines intersected at a common point on the *y*-axis while exhibiting progressively steeper slopes as the inhibitor concentration increased. This characteristic pattern indicates a competitive inhibition mechanism, where the inhibitors compete with the substrate for the enzyme’s active site. Consequently, the Km values increased with increasing inhibitor concentrations, reflecting a reduction in substrate affinity, while Vmax remained constant, confirming that the inhibition can be overcome by higher substrate levels.

The calculated inhibition constants (*K*_i_) further supported these findings, with values of 11.87 ± 3.31 μM for compound **12**, 9.08 ± 3.51 μM for compound **17**, and 5.49 ± 0.33 μM for compound **18**. Among these, compound **18** exhibited the strongest binding affinity toward the enzyme, suggesting a more effective competition with the substrate at the catalytic site. The relatively low *K*_i_ values observed for all three compounds indicate a high inhibitory potency against BChE.

Collectively, these results reveal that compounds **12**, **17**, and **18** act as reversible competitive inhibitors of BChE by occupying the active site and preventing substrate binding. This behavior aligns with the structural features predicted from molecular docking studies, reinforcing the conclusion that the interaction occurs primarily within the catalytic pocket. Therefore, these compounds may serve as promising scaffolds for the further design and development of potent and selective BChE inhibitors with potential therapeutic applications in neurodegenerative disorders such as Alzheimer’s disease.

### 2.5 Molecular Docking

Molecular docking analyses were performed to evaluate the binding affinity and interaction modes of the compounds with the target enzyme. In the analyses conducted with the Molegro Virtual Docker 6.0 program, compound **18** showed the highest binding score (MolDock score: −138.27), followed by compounds **12** (−128.81) and **17** (−111.27). These results indicated that compound **18** bound most strongly to the enzyme’s active site. It was thought that compound **12** established multifaceted interactions with the enzyme’s active site, attributed to the aromatic ring and halogen group in its structure, and also the conventional hydrogen bonds were observed, especially with the amino acid residues *Trp82, Gln*6*7, Asn83, Ser*1*98, Gly116*, and *Gly117*. In addition, alkyl interactions with *Trp*231 and *Leu28*6, π-alkyl interaction with *Trp231*, π-sulfur interactions with *Phe329* and *His438*, and halogen bond with *Leu28*6 also made important contributions (Table 3).

**Table 3:** Interactions between Compounds **12, 17**, and **18** with the BChE Active Site

types	category	**12**	**17**	**18**
hydrogen bond		Trp82, Gln67, Asn83, Ser198, Gly116, Gly117	Ile69, Gln71, Ser72	Asn83, Gln67, Asn68, Gly121, Trp82
hydrophobic	Pi–pi stacked		Tyr332, His438	Trp82
	Amide–pi stacked	Gly16		
	Alkyl	Trp231, Leu286		
	Pi–alkyl	Trp231	Ala328	
halogen		Gly117, Leu286		
π-sulfur		Phe329, His438	His438	His438
π-anion				Asp70
MolDock score		–128.81	–111.27	–138.27

These interactions supported the stable placement of the compounds in the active site. The bond between the halogen group in compound **12** and *Leu*2*8*6 revealed the binding stability (Figure 4a,b). Compound **17**, in which hydrogen bonds were limited to *Ile*6*9*, *Gln71*, and *Ser7*2, showed fewer interactions than the other compounds. The π–π interactions were observed with *Tyr332* and *His438*, while π–alkyl interaction occurred with *Ala328* and π–sulfur interaction occurred with *His438*. Although the aromatic groups in the molecular structure entering into π–sulfur interaction with *His438* slightly increased the binding affinity, the total interaction diversity and number were lower (Figure 4c,d). Compound **18** exhibited the most extensive profile in terms of both hydrogen bonds and hydrophobic interactions and achieved the lowest MolDock score. The hydrogen bonds with *Gln*6*7*, *Asn*6*8*, *Asn83*, *Gly1*2*1*, and *Trp82* ensured that the compound fits tightly into the active site. In addition, the π–π interaction with *Trp82*, the π–sulfur interaction with *His438*, the π–anion interaction with *Asp70*, and the van der Waals interactions with *Tyr332* were important interactions that increase the stability of the complex. In particular, the interactions between the large conjugated π-system in compound **18** and aromatic amino acids such as *Trp82* and *His438* strengthened the binding energy (Figure 4e,f). In conclusion, compound **18** exhibited a strong binding affinity with the enzyme active site due to the establishment of numerous and diverse bond interactions.

**Figure 4:**
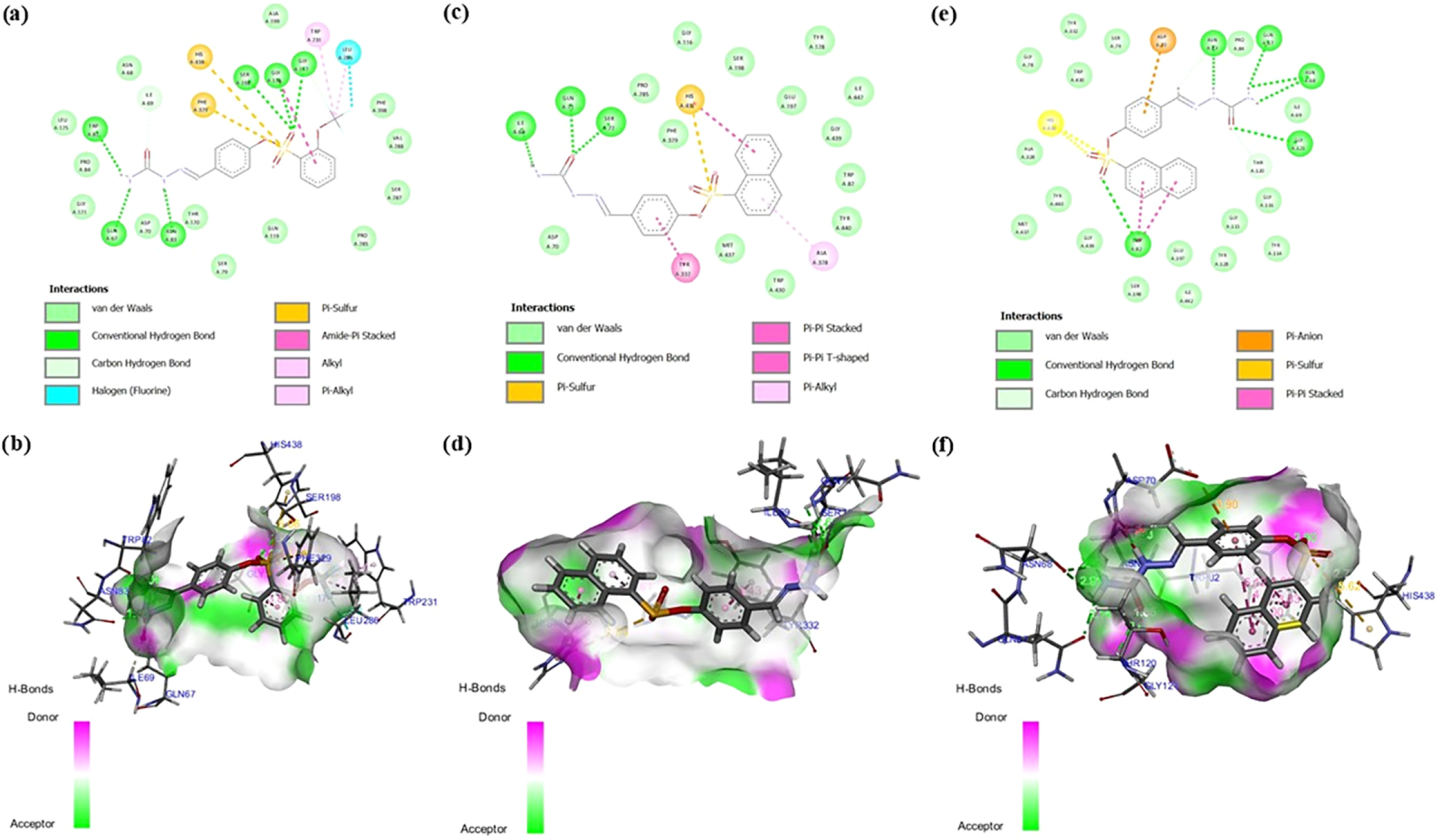
BChE binding modes of compounds **12** (a, b), **17** (c, d), and **18** (e, f) in both 2D and 3D configurations.

### 2.6 Molecular Dynamics Simulations

To assess the binding stability of bioactive compounds, molecular dynamics (MD) simulations were carried out for five systems: apo-4DBS, 4DBS-**Tacrine**, and 4DBS complexes with compounds **12**, **17**, and **18** (Figure 5), each simulated for 100 ns. The trajectories were analyzed using statistical metrics, including root-mean-square deviation (RMSD), root-mean-square fluctuation (RMSF), hydrogen bond interactions (with occupancy percentages), and MMGBSA binding energy calculations throughout the simulation time frame.

**Figure 5:**
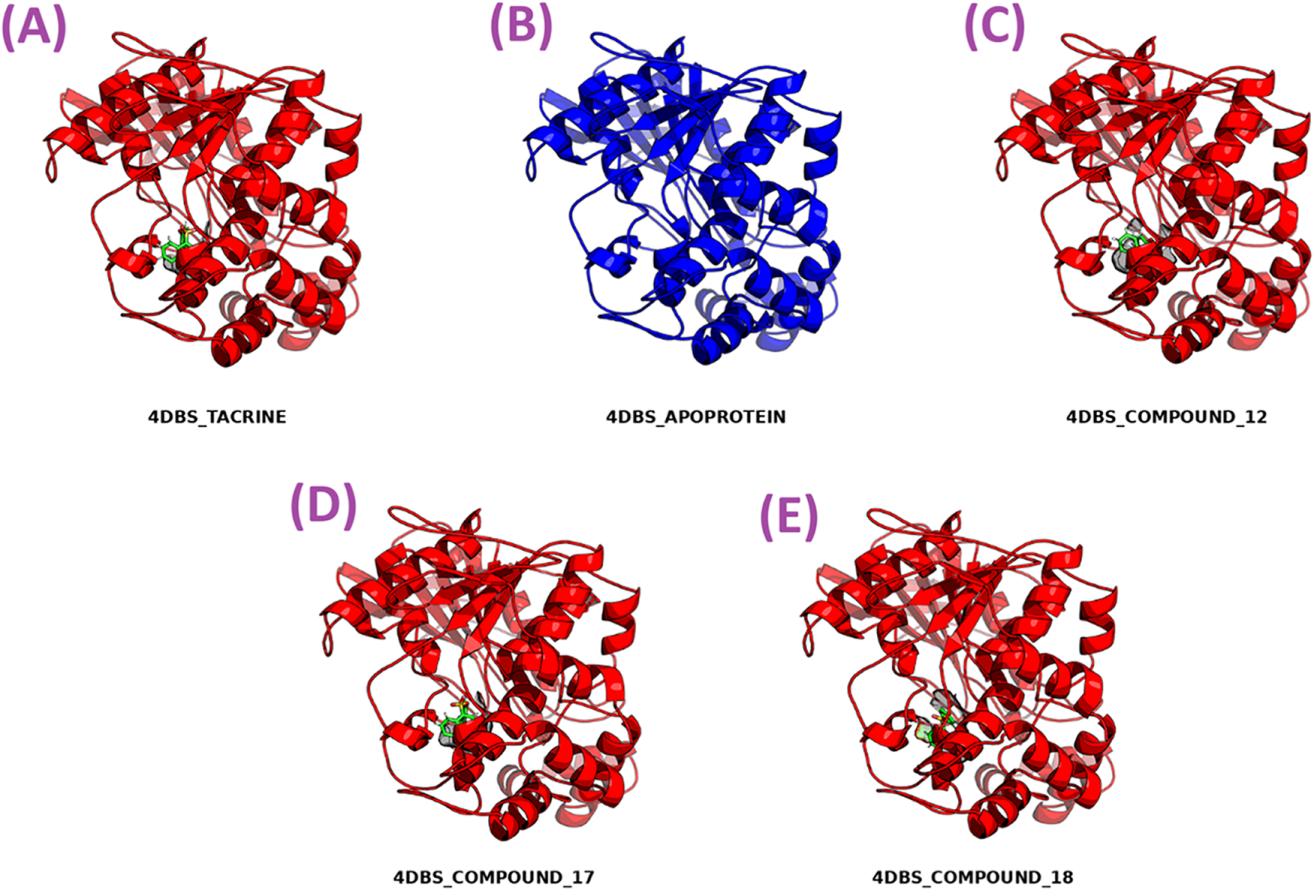
Graphical representation of protein and protein–ligand complexes: (A) 4DBS-Tacrine, (B) 4DBS Apoprotein, (C) 4DBS-Compound **12**, (D) 4DBS-Compound **17**, and (E) 4DBS-Compound **18**, where protein is shown in cartoon representation, and the ligand is shown in CPK representation with transparent surface.

#### 2.6.1 RMSD Analysis

The Cα-RMSD profiles showing the structural stability of the 4DBS protein upon binding with various ligands, including the native ligand Tacrine, which was docked at its known binding site, are presented in Figure 6. RMSD values stabilized across all systems after ∼20 ns, indicating convergence and equilibration of the simulations. The apo form exhibited the lowest mean RMSD (0.15 nm, Std: 0.01), confirming its intrinsic structural stability. Tacrine and compound **18** demonstrated low mean RMSD values (0.16 nm), comparable to the apoprotein, suggesting that these ligands maintain the native-like conformation of 4DBS with minimal perturbation. In contrast, compounds **12** and **17** showed slightly higher average RMSDs (0.18 and 0.19 nm, respectively) yet remained well below 0.3 nm, indicating acceptable structural integrity. Overall, these results implied that ligand binding, particularly with **Tacrine** and compound **18**, did not significantly disrupt the protein backbone, reinforcing the suitability of these compounds for further analysis.

**Figure 6:**
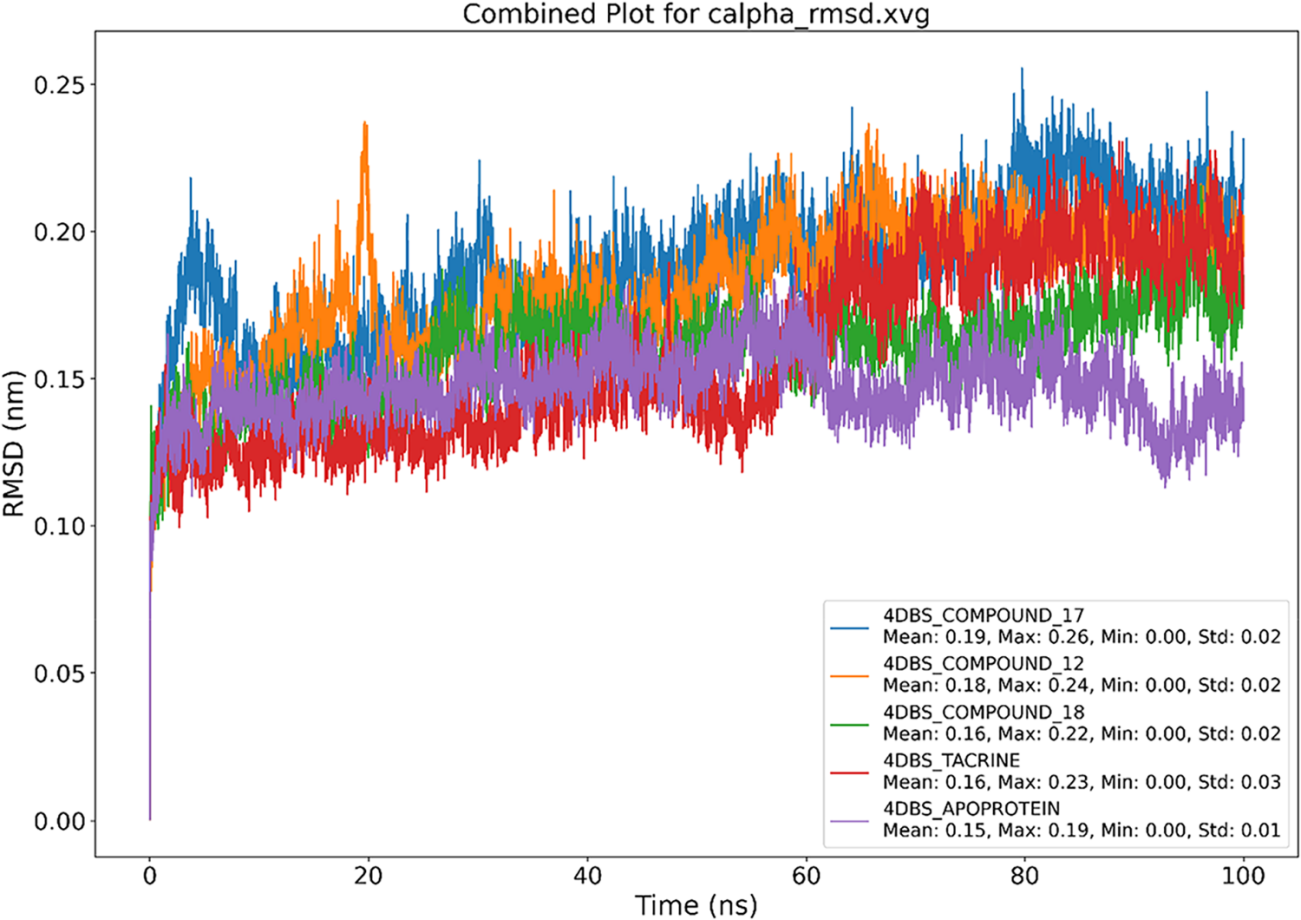
Graphical representation of the plots showing protein RMSD (nm) *versus* time (100 ns); (A) 4DBS-Tacrine, (B) 4DBS Apoprotein, (C) 4DBS-Compound **12**, (D) 4DBS-Compound **17**, and (E) 4DBS-Compound **18**.

The ligand RMSD trajectories over 100 ns (Figure 7) provided valuable insight into the conformational stability and binding persistence of the compounds within the 4DBS active site. Notably, 4DBS-Tacrine, the native ligand docked into its crystallographic position, exhibited a relatively low mean RMSD of 0.19 nm with a moderate standard deviation of 0.05, suggesting a stable binding pose and validating the docking protocol. Similarly, compounds **12** and **17** maintained low RMSD values (0.17 and 0.18 nm, respectively) with minimal deviations (Std: 0.04), indicating consistent and well-retained interactions throughout the simulation. In contrast, compound **18** showed a significantly higher mean RMSD of 0.29 nm and the largest fluctuation range (Max: 0.38 nm; Std: 0.09), implying less stable binding and larger conformational shifts. These results suggest that compounds **12** and **17** are promising candidates with stability profiles comparable to those of the native ligand, while **18** may require further optimization for enhanced binding persistence.

**Figure 7:**
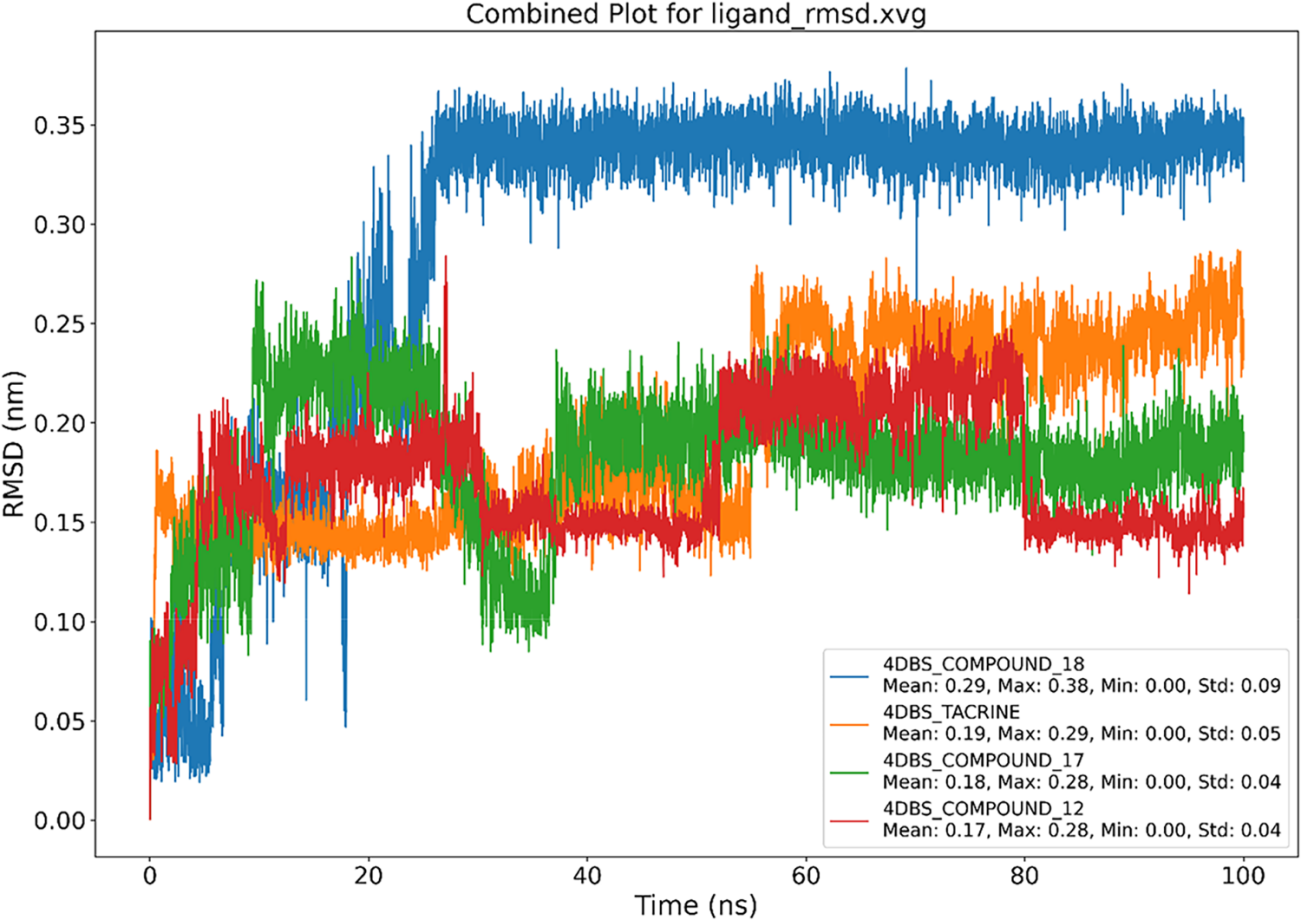
Graphical representation of the plots showing ligand RMSD (nm) *versus* time (100 ns) for (A) 4DBS-Tacrine, (B) 4DBS Apoprotein, (C) 4DBS-Compound **12**, (D) 4DBS-Compound **17**, and (E) 4DBS-Compound **18**.

#### 2.6.2 RMSF Analysis

The Cα-RMSF analysis (Figure 8) delineates residue-specific flexibility variations in the 4DBS protein across ligand-free (apo) and ligand-bound states during simulations. The apo form displayed the lowest overall fluctuations (Mean: 0.08 nm, Max: 0.35 nm), reflecting intrinsic structural rigidity. While global flexibility trends remained conserved across ligand-complexed systems, localized differences emerged. Notably, the native ligand Tacrine induced minimal perturbations (Mean: 0.09 nm, Std: 0.05), closely resembling the apo state. In contrast, compound **12** exhibited pronounced flexibility at the C-terminal region (∼residue 530), reaching a maximum of 0.79 nm, suggesting destabilization in the peripheral loop regions. Crucially, the core active site (residues 120–180) retained low RMSF values (<0.15 nm) in all systems, confirming that ligand binding—whether natural (**Tacrine**) or synthetic (**17/18**)—preserves the catalytic pocket’s stability. These results indicate that Tacrine, compound **17**, and compound **18** maintain native-like conformational dynamics, whereas compound **12** introduces localized flexibility outside the functional site, potentially influencing noncatalytic regions.

**Figure 8:**
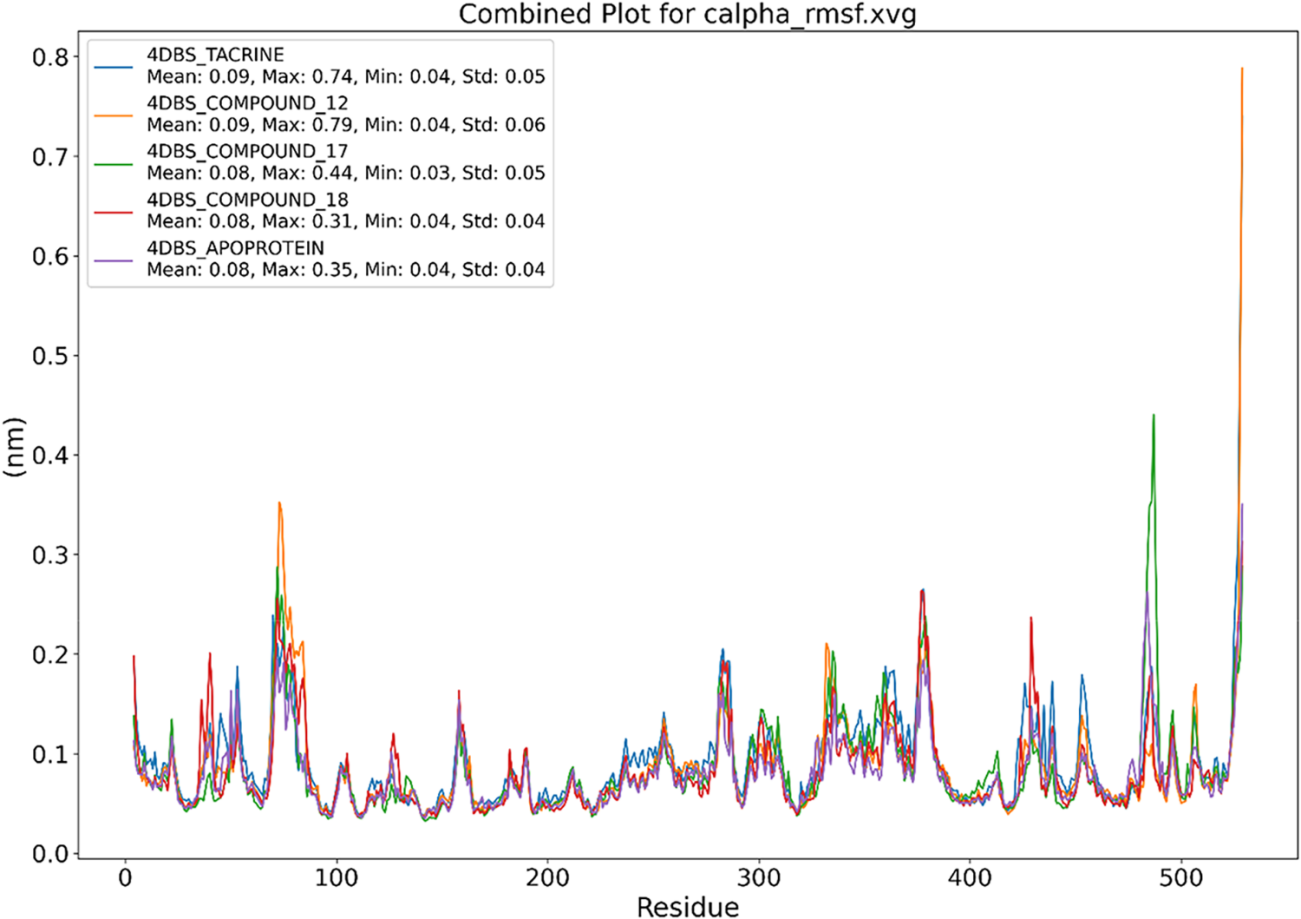
Graphical representation of the plots showing the protein RMSF (nm) *versus* residue index number of protein for (A) 4DBS-Tacrine, (B) **4DBS** Apoprotein, (C) 4DBS-Compound **12**, (D) 4DBS-Compound **17**, and (E) 4DBS-Compound **18**.

#### 2.6.3 H-Bond Interaction

Figure 9 illustrates the number of hydrogen bonds formed between the 4DBS protein and each ligand throughout the simulation, providing insights into binding consistency and stability. Compound **18** formed the highest average number of hydrogen bonds (1.93) with a maximum of 7, indicating frequent and stable interactions with the protein’s binding site. Compound **17** and the native ligand Tacrine followed with average hydrogen bond counts of 1.03 and 0.92, respectively, and maximum values of 6, suggesting moderately stable interactions, consistent with their favorable binding poses. Compound **12** showed the lowest hydrogen bonding performance (Mean: 0.62, Max: 4), reflecting fewer or less persistent contacts within the binding pocket. The relatively stable hydrogen bonding behavior of Tacrine, docked into its crystallographic binding site, further validates the simulation setup. Overall, the data highlight compound **18** as the ligand with the strongest hydrogen bonding profile, supporting its potential for high-affinity binding to 4DBS.

**Figure 9:**
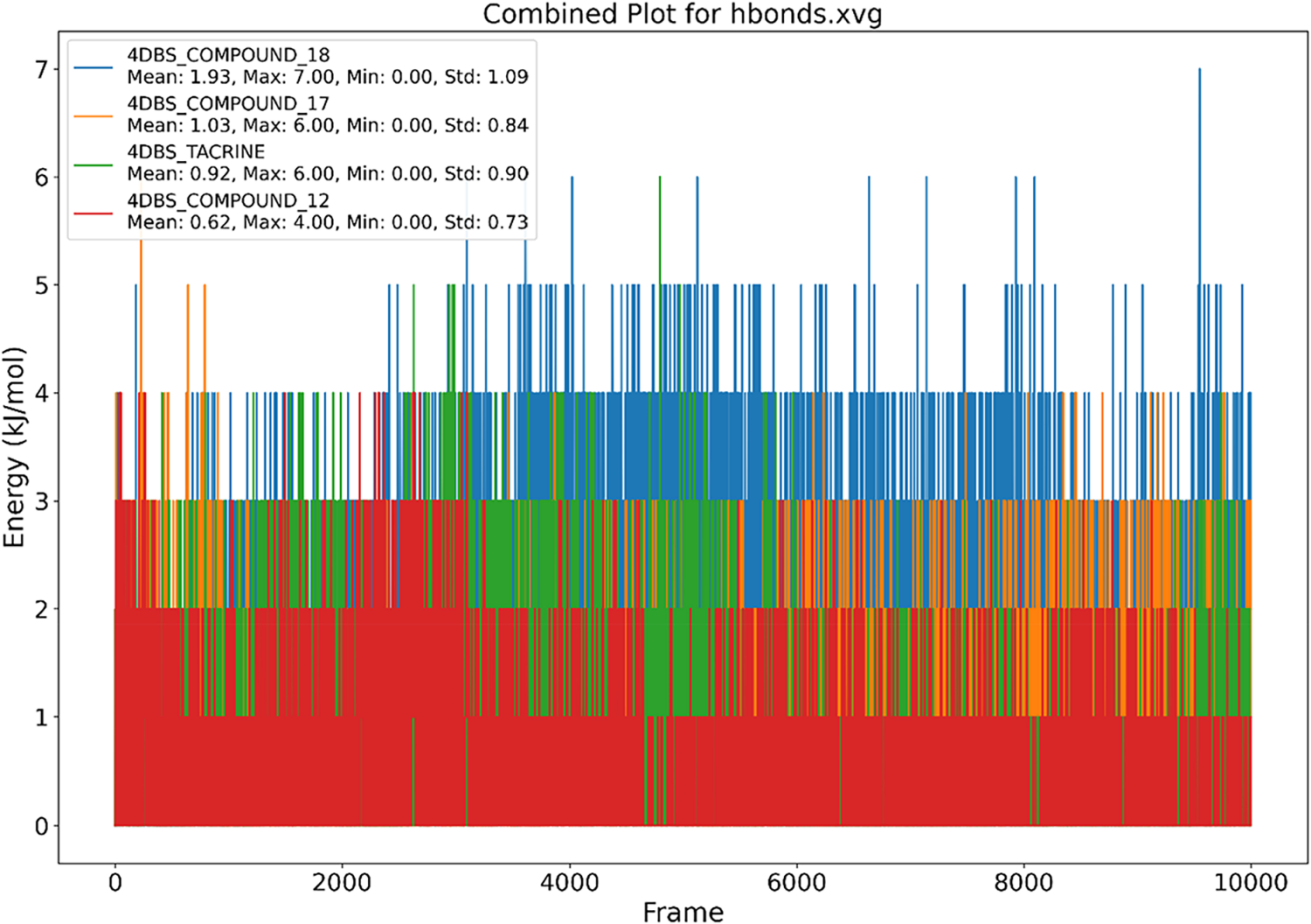
Pictorial representation of the number of h-bond contacts formed by (A) 4DBS-Tacrine, (B) 4DBS Apoprotein, (C) 4DBS-Compound **12**, (D) 4DBS-Compound **17**, and (E) 4DBS-Compound **18**.

Hydrogen bond occupancy analysis across ligand-bound 4DBS complexes (Figure 10) highlights distinct interaction patterns with critical residues, elucidating their binding mechanisms and stability. The native ligand Tacrine, bound to its crystallographic site, demonstrated moderate yet stable hydrogen bonding, primarily with *Thr120*, *Tyr332*, and *Tyr440*, with peak occupancy of ≤2%, reflecting dynamic but stable interactions characteristic of natural ligand–protein associations. Compound **17** displayed the most robust interactions, forming highly stable hydrogen bonds with *Thr120* (29% occupancy) and *Tyr440* (38% occupancy), underscoring its targeted binding to conserved active-site residues. Compound **18** exhibited notable interactions, including strong engagement with *Tyr114* (22% occupancy) and moderate bonding with *Thr120*, *Trp112*, and *Gly11*6, suggesting a wider interaction network within the active site. While compound **12** showed weaker overall hydrogen bonding, it maintained modest interactions with *Gly439*, *Thr120*, and *Tyr440*, aligning with its lower overall interaction capacity observed in time-dependent analyses. These results emphasize *Thr120* and *Tyr440* as pivotal anchoring points for ligand binding, with compound **17** demonstrating superior stability through focused, high-occupancy hydrogen bonds in the catalytic pocket.

**Figure 10:**
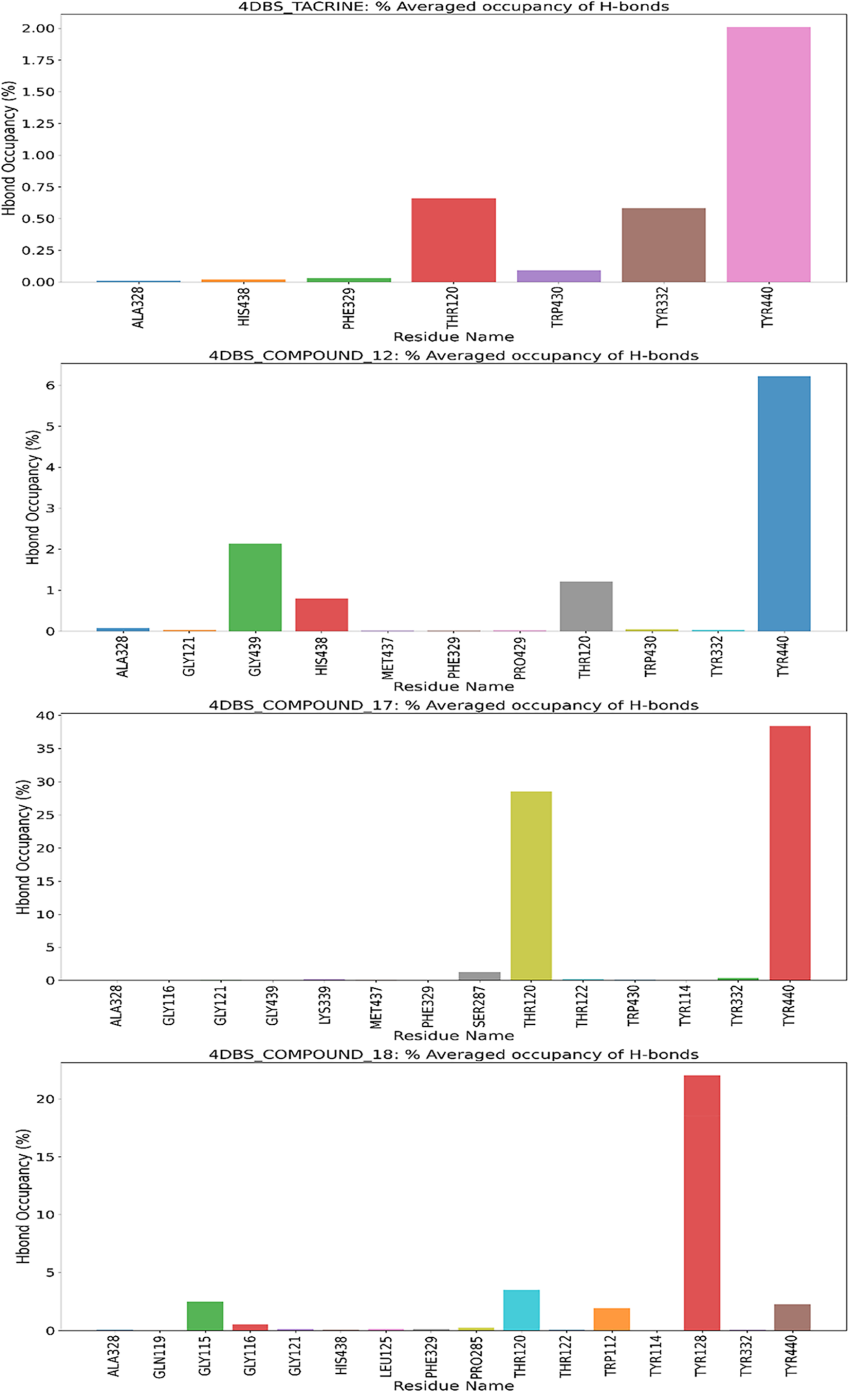
Histogram representation of % occupancies of the H-bond protein–ligand contacts of (A) 4DBS-Tacrine, (B) 4DBS Apoprotein, (C) 4DBS-Compound **12**, (D) 4DBS-Compound **17**, and (E) 4DBS-Compound **18**.

#### 2.6.4 MMGBSA Calculations

Figure 11 illustrates the binding energy profiles, offering a comparative analysis of the ligand interactions with the 4DBS protein during simulations. Compound **17** emerged as the strongest binder, displaying the lowest average binding energy (−54.74 kJ/mol) and a peak interaction strength of −99.90 kJ/mol, signifying robust, consistent engagement with the protein. Compounds **18** (−53.28 kJ/mol) and **12** (−48.98 kJ/mol) followed, both showing stable binding but with marginally greater variability. The crystallographically docked native ligand Tacrine had a moderate average energy of −47.57 kJ/mol, aligning with its experimentally confirmed affinity but underperforming relative to those of the leading compounds. The ligand-free apo system recorded a similar mean energy (−46.24 kJ/mol), implying that nonspecific interactions prevail without a bound ligand. These results position compound **17** as the most thermodynamically stable binder, surpassing even the native ligand in predicted efficacy, and underscore its viability as a prospective inhibitory agent.

**Figure 11:**
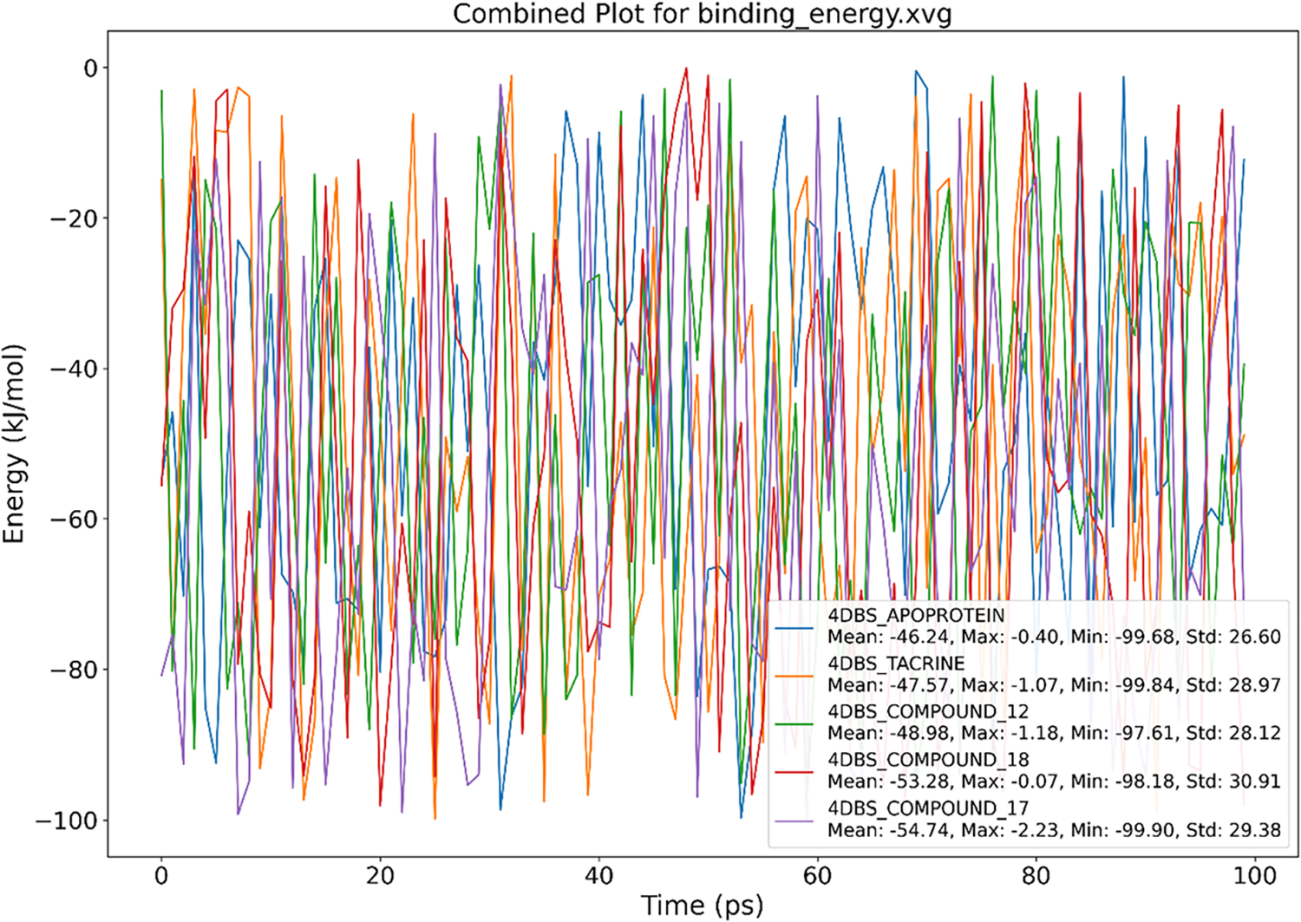
MMGBSA Δ*G* binding energy calculations for (A) 4DBS-Tacrine, (B) 4DBS Apoprotein, (C) 4DBS-Compound **12**, (D) 4DBS-Compound **17**, and (E) 4DBS-Compound **18**.

### 2.7 ADMET

A molecule must reach its target location in the body in a high enough concentration and stay in a physiologically active state for an extended period of time in order to elicit the desired biological reaction.^41^ Due to limited access to physical samples, predicting ADMET properties with computer-aided models at an early stage in the drug development process offers an important alternative.^42^ In this study, the ADMET properties of compounds **12, 17**, and **18** were calculated using online tools such as SwissADME, ProTox-II, and PKCS,M and these results are given in Table 4.

**Table 4:** ADMET Profile of Compounds **12, 17**, and **18**

	value		
properties	**12**	**17**	**18**
molecular weight (g/mol)	403.33	369.39	369.39
rotatable bonds	8	6	6
consensus log *P*_o/w_	2.55	2.59	2.58
topological polar surface area (TPSA, Å)	128.46	119.23	119.23
water solubility (log mol/L)	–4.618	–4.219	–4.237
intestinal absorption (human) (%)	81.26	86.121	89.338
BBB permeant	No	No	No
predicted LD_50_ (mg/kg)	10750	10750	10750
predicted toxicity class	6	6	6
	**Prediction**	**Prediction**	**Prediction**
hepatotoxicity	inactive	inactive	inactive
immunotoxicity	inactive	inactive	inactive
cytotoxicity	inactive	inactive	inactive
carcinogenicity	inactive	active	active
mutagenicity	inactive	inactive	inactive
Caco-2 permeability (log Papp in 10^–6^ cm/s)	0.865	0.889	0.979
AMES toxicity	no	yes	yes
P-gp substrate	yes	yes	yes
Lipinski	yes; 0 violation	yes; 0 violation	yes; 0 violation

Accordingly, compounds **12**, **17**, and **18** meet Lipinski’s “drug-likeness” rules without violating them, which is a positive indicator in terms of oral bioavailability. Their molecular weights are in the range of 369.39–403.33 g/mol, and the rotatable bond numbers vary between 6 and 8, indicating that they can establish a balance between structural flexibility and target site interaction potential. The efficacy of drugs targeting the central nervous system is predicated on their ability to traverse the blood–brain barrier (BBB). However, no compounds have the capacity to penetrate this physiological barrier. Their water solubility values were similar, and although they showed low solubility, they were within acceptable limits. Their intestinal absorption rates were high (81.26 to 89.34%), indicating that the compounds could be effectively absorbed from the gastrointestinal tract. It was observed that compounds **12, 17**, and **18** had good oral absorption rates in the human body. The Ames test is employed to evaluate the mutagenic potential of a drug or compound by determining its ability to cause DNA damage, thereby serving as an important indicator of genetic toxicity. According to the toxicity parameters, compound **12** was negative for AMES, indicating that the mutagenic potential was low. On the other hand, compounds **17** and **18** gave positive AMES results, which was important for their potential genotoxic effects. For optimal polarity-related properties, the topological polar surface area (TPSA) should generally be in the range of 20–130 Å^2^. Compounds **12, 17**, and **18** exhibited TPSA values within this acceptable range, indicating favorable polarity for potential bioavailability. Caco-2 value is a remarkable parameter for human colorectal cancer cell lines. This cell line is used as a model that mimics the intestinal epithelial barrier. The Caco-2 permeability values of compounds **12, 17**, and **18** varied between 0.865 and 0.979. LD50, in toxicology, is the amount of a drug that causes the death of 50% of the total population when given at once. As can be seen from Table 4, the estimated LD_50_ value of these compounds was determined as 10750 mg/kg. A high value indicates that the acute toxicity of the compound is low and therefore may have a positive potential in terms of safety profile.^43^ According to Protox-II estimation, all three molecules are classified as toxicity class 6 and are not expected to exhibit harmful effects if ingested (ref: 10.1093/nar/gky318). Additionally, the ProTox-II web server predicts five toxicological parameters: hepatotoxicity, carcinogenicity, immunotoxicity, mutagenicity, and cytotoxicity. According to the results, all three compounds were considered inactive in terms of hepatotoxic, immunotoxic, cytotoxic, and mutagenic effects (Table 4). In contrast, compounds **17** and **18** were predicted to be carcinogenic, while compound **12** was predicted to be noncarcinogenic. These findings indicate that the investigated compounds generally have a favorable toxicological profile with low acute toxicity potential.

## 3 Experimental Section

### 3.1 Reagents and Instruments

All starting materials and reagents were obtained from Sigma-Aldrich and Merck in analytical-grade purity and used without any purification. The progress of the reactions and the purity of the products were monitored by thin-layer chromatography (TLC) on silica gel 60 *F*_254_ plates (20 cm × 20 cm, 0.25 mm thickness, Merck). Hexane:ethyl acetate (2:1, v/v) was employed as the mobile phase, and chromatographic development was carried out at room temperature in a solvent-saturated chamber. Infrared (IR) spectra were recorded on a PerkinElmer Spectrum-100 FT-IR spectrometer equipped with a Universal ATR Sampling Accessory in the range of 4000–400 cm^–1^, using approximately 2 mg of each solid sample. The ^1^H and ^13^C NMR spectra of the compounds (**1–18**) were obtained with Bruker AVANCE III 400 and 600 MHz spectrometers. Samples (≈20 mg) were dissolved in DMSO-*d*_*6*_, and the chemical shifts (δ) were reported in parts per million (ppm) relative to tetramethylsilane (TMS) as the internal reference. Mass spectra (MS) were obtained using a liquid chromatography–tandem mass spectrometer (LC–MS/MS, Shimadzu 8045) operated in electrospray ionization (ESI) mode. To determine the purity of the compounds, an Agilent 1260 Infinity HPLC instrument was used with a C18 column (3 μm, 4.6 mm × 50 mm) under gradient elution conditions using an acetonitrile/water (80:20) mobile phase. Analysis by integrating the areas of the main peaks detected at 254 nm revealed purities of ≥ 90% for all tested compounds (Figures S76–S93).

### 3.2 General Procedure for the Synthesis of 4-Formylphenyl-Substituted Sulfonates (**A1–A18**)

A mixture of 4-hydroxybenzaldehyde (1 mmol) and triethylamine (TEA, 2 mmol) was dissolved in dichloromethane (DCM, 10 mL) and stirred vigorously at room temperature for 45 min. Subsequently, substituted benzenesulfonyl chloride (1 mmol) in DCM (10 mL) was added dropwise, and the reaction mixture was refluxed for approximately 5 h. The progress of the reaction was monitored by thin-layer chromatography (TLC). After being completed, the mixture was cooled to room temperature and extracted twice with 2 M HCl. The combined organic layers were dried over anhydrous sodium sulfate and concentrated under reduced pressure. The crude product was purified by recrystallization from ethanol to afford the compound in pure form..^44–46^ The ^1^H NMR spectra of the intermediate 4-formylphenyl-substituted sulfonates (**A8, A12**, and **A13**), reported here for the first time, are included in the Supporting Information (Figures S1–S3). The other 4-formylphenyl-substituted sulfonates were previously reported.^47–49^

#### 3.2.1 4-Formylphenyl 2-(Trifluoromethyl)­benzenesulfonate (**A8**)

^1^H NMR (600 MHz, DMSO-*d*_6_) δ ppm: 9.99 (*s*, 1H, *H*C=O), 8.25 (*t*, 2H, *J*_1_ = 8.0, *J*_2_ = 8.4 Hz, Ar*H*), 8.15 (*s*, 1H, Ar*H*), 7.97 (*d*, 3H, *J* = 8.8 Hz, Ar*H*), 7.35 (*d*, 2H, *J* = 8.8 Hz, Ar*H*).

#### 3.2.2 4-Formylphenyl 2-(Trifluoromethoxy)­benzenesulfonate (**A12**)

^1^H NMR (600 MHz, DMSO-*d*_6_) δ ppm: 9.98 (*s*, 1H, *H*C=O), 7.97 (*d*, *J* = 7.4 Hz, 4H, Ar*H*), 7.79 (*d*, *J* = 7.4 Hz, 1H, Ar*H*), 7.63 (*t*, *J* = 7.3, 7.3 Hz, 1H, Ar*H*), 7.32 (*d*, *J* = 7.8 Hz, 2H, Ar*H*).

#### 3.2.3 4-Formylphenyl 3-(Trifluoromethoxy)­benzenesulfonate (**A13**)

^1^H NMR (600 MHz, DMSO-*d*_6_) δ ppm: 9.99 (*s*, 1H, *H*C=O), 7.98 (*s*, 1H, Ar*H*), 7.96 (*d*, 2H, *J* = 8.7 Hz, Ar*H*), 7.84–7.91 (*m*, 3H, Ar*H*), 7.33 (*d*, 2H, *J* = 8.5 Hz, Ar*H*).

### 3.3 General Procedure for the Synthesis of Semicarbazone-Sulfonate Derivatives (**1–18**)

Semicarbazide hydrochloride (1 mmol) and sodium acetate (2 mmol) were added to a solution of 4-formylphenyl-substituted sulfonates (1 mmol, **A1–A18**) in methanol (10 mL) with continuous stirring. The reaction mixture was stirred until the formation of a precipitate, which was subsequently filtered, washed with cold methanol, dried, and recrystallized from ethanol to afford the corresponding semicarbazone-sulfonate derivatives. The purity and completion of the reaction were confirmed by thin-layer chromatography (TLC, single spot).^50^ All spectral data employed for the structural characterization of the compounds are provided in the Supporting Information (Figures S4–S75).

#### 3.3.1 4-[(*E*)-(2-Carbamoylhydrazinylidene)­methyl]­phenyl Benzenesulfonate (**1**)

White solid, yield: 81%, mp: 227–228 °C. IR (ν, cm^–1^): 3480, 3435 (N–H); 3070 (Ar–CH); 2988, 2923 (aliphatic CH); 1695 (C=O); 1585 (C=N stretching band); 1498, 1472 (Ar–C=C); 1370, 1199 (SO_2_); 688 (Ar–CH). ^1^H NMR (600 MHz, DMSO-*d*_6_) δ ppm: 10.32 (*s*, 1H, N*H*), 7.86 (*d*, *J* = 7.6 Hz, 2H, Ar*H*), 7.82 (*t*, *J* = 7.6 Hz, 1H, Ar*H*), 7.78 (*s*, 1H, N=C*H*), 7.72 (d, *J* = 8.7 Hz, 2H, Ar*H*), 7.67 (*t*, *J* = 7.8 Hz, 2H, Ar*H*), 7.00 (*d*, *J* = 8.6 Hz, 2H, Ar*H*), 6.53 (*s*, 2H, N*H*_2_). ^13^C NMR (151 MHz, DMSO-*d*_6_) δ ppm: 157.12 (*C*=O), 149.52 (*C*=N), 138.00, 135.58, 134.64, 131.82, 130.30, 128.73, 128.45, 122.71 (Ar*C*). ESI-MS (*m*/*z*): 318 [M – H]^+^. Elemental analysis, C_14_H_13_N_3_O_4_S (319,34 g/mol). Found, %: C, 52.77; H, 4.41; N, 13.40; S, 10.23. Calculated, %: C, 52.66; H, 4.10; N, 13.16; S, 10.04. HPLC: purity 90%.

#### 3.3.2 4-[(*E*)-(2-Carbamoylhydrazinylidene)­methyl]­phenyl 4-Fluorobenzene-1-sulfonate (**2**)

White solid, yield: 86%, mp: 205–206 °C. IR (ν, cm^–1^): 3491, 3405 (N–H); 3051 (Ar–CH); 2969, 2923 (aliphatic CH); 1694 (C=O); 1591 (C=N stretching band); 1574, 1495 (Ar–C=C); 1371, 1150 (SO_2_);1091 (Ar–C–F); 688 (Ar–CH). ^1^H NMR (600 MHz, DMSO-*d*_6_) δ ppm: 10.35 (*s*, 1H, N*H*), 7.94 (*dd*, *J* = 8.8, 5.0 Hz, 2H, Ar*H*), 7.79 (*s*, 1H, N=C*H*), 7.74 (*d*, *J* = 8.7 Hz, 2H, Ar*H*), 7.51 (*t*, *J* = 8.7 Hz, 2H, Ar*H*), 7.02 (*d*, *J* = 8.7 Hz, 2H, Ar*H*), 6.56 (*s*, 2H, N*H*_2_). ^13^C NMR (151 MHz, DMSO-*d*_6_) δ ppm: 166.92 (*C*=O), 165.23, 157.17, 149.43 (*C*=N), 138.05, 134.70, 132.18, 128.51, 122.78, 117.73 (ArC). ESI-MS (*m*/*z*): 336 [M – H]^+^. Elemental analysis, C_14_H_12_FN_3_O_4_S (337,33 g/mol). Found, %: C, 50.09; H, 3.65; N, 12.60; S, 10.00. Calculated, %: C, 49.85; H, 3.59; N, 12.46; S, 9.50. HPLC: purity 98%.

#### 3.3.3 4-[(*E*)-(2-carbamoylhydrazinylidene)­methyl]­phenyl 4-Chlorobenzene-1-sulfonate (**3**)

White solid, yield: 84%, mp: 213–215 °C. IR (ν, cm^–1^): 3492, 3294 (N–H); 3095 (Ar–CH); 2968, 2925 (aliphatic CH); 1692 (C=O); 1575 (C=N stretching band); 1497, 1481 (Ar–C=C); 1373, 1152 (SO_2_); 1087 (Ar–C–Cl); 680 (Ar–CH). ^1^H NMR (600 MHz, DMSO-*d*_6_) δ ppm: 10.35 (*s*, 1H, N*H*), 7.87 (*d*, *J* = 7.3 Hz, 2H, Ar*H*), 7.80 (*s*, 1H, N=C*H*), 7.75 (*d*, *J* = 8.5 Hz, 4H, Ar*H*), 7.04 (*d*, *J* = 7.5 Hz, 2H, Ar*H*), 6.55 (*s*, 2H, N*H*_2_). ^13^C NMR (151 MHz, DMSO-*d*_6_) δ ppm: 157.14 (*C*=O), 149.38 (*C*=N), 140.64, 138.00, 134.77, 133.39, 130.71, 130.49, 128.54, 122.77 (Ar*C*). ESI-MS (*m*/*z*): 353 [M – H]^+^. Elemental analysis, C_14_H_12_ClN_3_O_4_S (353,78 g/mol). Found, %: C, 47.38; H, 3.25; N, 11.65; S, 9.18. Calculated, %: C, 47.53; H, 3.42; N, 11.88; S, 9.06. HPLC: purity 93%.

#### 3.3.4 4-[(*E*)-(2-Carbamoylhydrazinylidene)­methyl]­phenyl 4-Bromobenzene-1-sulfonate (**4**)

White solid, yield: 89%, mp: 219–220 °C. IR (ν, cm^–1^): 3491, 3288 (N–H); 3025 (Ar–CH); 2972, 2902 (aliphatic CH); 1692 (C=O); 1574 (C=N stretching band); 1531, 1449 (Ar–C=C); 1070 (Ar–C–Br); 1372, 1151 (SO_2_); 670 (Ar–CH). ^1^H NMR (400 MHz, DMSO-*d*_6_) δ ppm: 10.33 (*s*, 1H, N*H*), 7.90 (*t*, *J* = 8.5 Hz, 2H, Ar*H*), 7.80–7.74 (*m*, 5H, Ar*H*), 7.04 (*t*, *J* = 9.0 Hz, 2H, Ar*H*), 6.53 (*s*, 2H, N*H*_2_). ^13^C NMR (101 MHz, DMSO-*d*_6_) δ ppm: 157.13 (*C*= O), 149.35 (*C*=N), 137.92, 134.78, 133.78, 133.44, 130.69, 129.84, 128.55, 122.78 (Ar*C*). ESI-MS (*m*/*z*): 398 [M+2]^+^. Elemental analysis, C_14_H_12_BrN_3_O_4_S (396,97 g/mol). Found, %: C, 42.30; H, 3.11; N, 10.72; S, 8.33. Calculated, %: C, 42.23; H, 3.04; N, 10.55; S, 8.05. HPLC: purity 99%.

#### 3.3.5 4-[(*E*)-(2-Carbamoylhydrazinylidene)­methyl]­phenyl 4-Iodobenzene-1-sulfonate (**5**)

White solid, yield: 91%, mp: 106–107 °C. IR (ν, cm^–1^): 3485, 3145 (N–H); 3063 (Ar–CH); 2919 (aliphatic CH); 1745 (C=O); 1567 (C=N stretching band); 1504, 1446 (Ar–C=C); 1088 (Ar–C–I); 1361, 1198 (SO_2_); 669 (Ar–CH). ^1^H NMR (400 MHz, DMSO-*d*_6_) δ ppm: 10.33 (*s*, 1H, N*H*), 8.06 (*d*, *J* = 8.6 Hz, 2H, Ar*H*), 7.78 (*s*, 1H, N=C*H*), 7.74 (*d*, *J* = 8.7 Hz, 2H, Ar*H*), 7.59 (*d*, *J* = 8.6 Hz, 2H, Ar*H*), 7.02 (*d*, *J* = 8.7 Hz, 2H, Ar*H*), 6.54 (*s*, 2H, N*H*_2_). ^13^C NMR (101 MHz, DMSO-*d*_6_) δ ppm: 157.12 (*C*=O), 149.37 (*C*=N), 139.22, 137.92, 134.74, 134.13, 130.16, 128.54, 122.75, 104.78 (Ar*C*). ESI-MS (*m*/*z*): 445 [M – H]^+^. Elemental analysis, C_14_H_12_IN_3_O_4_S (445,23 g/mol). Found, %: C, 37.91; H, 2.80; N, 9.49; S, 7.28. Calculated, %: C, 37.77; H, 2.72; N, 9.44; S, 7.20. HPLC: purity 98%.

#### 3.3.6 4-[(*E*)-(2-Carbamoylhydrazinylidene)­methyl]­phenyl 4-Methylbenzene-1-sulfonate (**6**)

White solid, yield: 94%, mp: 203–205 °C. IR (ν, cm^–1^): 3492, 3297 (N–H); 3090 (Ar–CH); 2972, 2925 (aliphatic CH); 1690 (C=O); 1596 (C=N stretching band); 1574, 1497 (Ar–C=C); 1365, 1152 (SO_2_); 686 (Ar–CH). ^1^H NMR (600 MHz, DMSO-*d*_6_) δ ppm: 10.34 (*s*, 1H, N*H*), 7.79 (*s*, 1H, N=C*H*), 7.74 (*d*, *J* = 7.8 Hz, 4H, Ar*H*), 7.47 (*d*, *J* = 8.0 Hz, 2H, Ar*H*), 7.00 (*d*, *J* = 8.5 Hz, 2H, Ar*H*), 6.55 (*s*, 2H, N*H*_2_), 2.42 (*s*, 3H, C*H*_3_). ^13^C NMR (151 MHz, DMSO-*d*_6_) δ ppm: 157.16 (*C*=O), 149.60 (*C*=N), 146.35, 138.10, 134.51, 131.74, 130.70, 128.75, 128.44, 122.72 (Ar*C*), 21.63 (*C*H_3_). ESI-MS (*m*/*z*): 332 [M – H]^+^. Elemental analysis, C_15_H_15_N_3_O_4_S (333,36 g/mol). Found, %: C, 54.17; H, 4.63; N, 12.81; S, 9.70. Calculated, %: C, 54.04; H, 4.54; N, 12.61; S, 9.62. HPLC: purity 98%.

#### 3.3.7 4-[(*E*)-(2-Carbamoylhydrazinylidene)­methyl]­phenyl 4-Methoxybenzene-1-sulfonate (**7**)

White solid, yield: 87%, mp: 173–175 °C. IR (ν, cm^–1^): 3485, 3145 (N–H); 3063 (Ar–CH); 2972 (aliphatic CH); 1693 (C=O); 1596 (C=N stretching band); 1574, 1445 (Ar–C=C); 1350, 1154 (SO_2_); 1028 (Ar–C–OCH_3_); 686 (Ar–CH). ^1^H NMR (400 MHz, DMSO-*d*_6_) δ ppm: 10.31 (*s*, 1H, N*H*), 7.75 (*dd*, *J* = 16.1, 8.7 Hz, 6H, Ar*H*), 7.16 (*d*, *J* = 9.0 Hz, 2H, Ar*H*), 6.98 (*d*, *J* = 8.7 Hz, 2H, Ar*H*), 6.52 (*s*, 2H, N*H*_2_), 3.86 (*s*, 3H, C*H*_3_). ^13^C NMR (101 MHz, DMSO-*d*_6_) δ ppm: 164.52 (*C*=O), 157.12, 149.62 (*C*=N), 138.01, 134.46, 131.18, 128.42, 125.75, 122.79, 115.42 (Ar*C*), 56.41 (O*C*H3). ESI-MS (*m*/*z*): 348 [M – H]^+^. Elemental analysis, C_15_H_15_N_3_O_5_S (349,36 g/mol). Found, %: C, 51.70; H, 4.56; N, 12.31; S, 9.05. Calculated, %: C, 51.57; H, 4.33; N, 12.03; S, 9.18. HPLC: purity 99%.

#### 3.3.8 4-[(*E*)-(2-Carbamoylhydrazinylidene)­methyl]­phenyl 2-(Trifluoromethyl)­benzene-1-sulfonate (**8**)

White solid, yield: 83%, mp: 204–206 °C. IR (ν, cm^–1^): 3447, 3329 (N–H); 3080 (Ar–CH); 2988 (aliphatic CH); 1694 (C=O); 1570 (C=N stretching band); 1499, 1441 (Ar–C=C); 1381, 1172 (SO_2_); 1146, 1122 (Ar–C–CF_3_); 688 (Ar–CH). ^1^H NMR (400 MHz, DMSO-*d*_6_) δ ppm: 10.34 (*s*, 1H, N*H*), 8.21 (*d*, *J* = 7.4 Hz, 1H, Ar*H*), 8.05 (*t*, *J* = 7.2 Hz, 2H, Ar*H*), 7.92 (*t*, *J* = 7.5 Hz, 1H, Ar*H*), 7.79 – 7.74 (*m*, 3H, Ar*H*), 7.03 (*d*, *J* = 8.6 Hz, 2H, Ar*H*), 6.54 (*s*, 2H, N*H*_2_). ^13^C NMR (101 MHz, DMSO-*d*_6_) δ ppm: 157.09 (*C*=O), 149.19 (*C*=N), 142.58, 137.85, 136.35, 134.89, 134.21, 133.52, 132.66, 129.82, 128.63, 127.72, 122.52 (Ar*C*). ESI-MS (*m*/*z*): 386 [M – H]^+^. Elemental analysis, C_15_H_12_F_3_N_3_O_4_S (387,33 g/mol). Found, %: C, 46.63; H, 14.85; N, 10.68; S, 8.33. Calculated, %: C, 46.51; H, 14.71; N, 10.85; S, 8.28. HPLC: purity 99%.

#### 3.3.9 4-[(*E*)-(2-Carbamoylhydrazinylidene)­methyl]­phenyl-3-(trifluoromethyl)­benzene-1-sulfonate (**9**)

Off-white solid, yield: 87%, mp: 38–40 °C. IR (ν, cm^–1^): 3512, 3483 (N–H); 3063 (Ar–CH); 2972 (aliphatic CH); 1694 (C=O); 1569 (C=N stretching band); 1496, 1473 (Ar–C=C); 1382, 1165 (SO_2_); 692 (Ar–CH). ^1^H NMR (400 MHz, DMSO-*d*_6_) δ ppm: 10.33 (*s*, 1H, N*H*), 7.87 (*dd*, *J* = 25.7, 11.2 Hz, 4H, Ar*H*), 7.79 – 7.73 (*m*, 3H, Ar*H*), 7.05 (*d*, *J* = 5.9 Hz, 2H, Ar*H*), 6.54 (*s*, 2H, N*H*_2_). ^13^C NMR (101 MHz, DMSO-*d*_6_) δ ppm: 170.28 (*C*=O), 157.10, 149.26 (*C*=N), 149.25, 148.83, 137.84, 136.35, 134.87, 132.91, 128.54, 128.11, 122.69, 121.40 (Ar*C*). ESI-MS (*m*/*z*): 386 [M – H]^+^. Elemental analysis, C_15_H_12_F_3_N_3_O_4_S (387,33 g/mol). Found, %: C, 46.46; H, 14.76; N, 10.89; S, 8.35. Calculated, %: C, 46.51; H, 14.71; N, 10.85; S, 8.28. HPLC: purity 98%.

#### 3.3.10 4-[(*E*)-(2-Carbamoylhydrazinylidene)­methyl]­phenyl-4-(trifluoromethyl)­benzene-1-sulfonate (**10**)

White solid, yield: 90%, mp: 38–40 °C. IR (ν, cm^–1^): 3482, 3301 (N–H); 3072 (Ar–CH); 2988 (aliphatic CH); 1697 (C=O); 1587 (C=N stretching band); 1496, 1435 (Ar–C=C); 1380, 1152 (SO_2_); 1124, 1085 (Ar–C–CF_3_); 629 (Ar–CH). ^1^H NMR (400 MHz, DMSO-*d*_6_) δ ppm: 10.35 (*s*, 1H, N*H*), 8.09 (*dd*, *J* = 12.8, 6.6 Hz, 4H, Ar*H*), 7.80–7.75 (*m*, 3H, Ar*H*), 7.06 (*d*, *J* = 10.9 Hz, 2H, Ar*H*), 6.55 (*s*, 2H, N*H*_2_). ^13^C NMR (101 MHz, DMSO-*d*_6_) δ ppm: 192.38, 157.12 (*C*=O), 153.10, 149.26 (*C*=N), 138.37, 135.61, 132.06, 129.86, 127.60, 123.39, 122.72 (Ar*C*). Elemental analysis, C_15_H_12_F_3_N_3_O_4_S (387,33 g/mol). Found, %: C, 46.39; H, 14.70; N, 10.88; S, 8.37. ESI-MS (*m*/*z*): 386 [M – H]^+^. Calculated, %: C, 46.51; H, 14.71; N, 10.85; S, 8.28. HPLC: purity 98%.

#### 3.3.11 4-[(*E*)-(2-Carbamoylhydrazinylidene)­methyl]­phenyl 3,5-Bis­(trifluoromethyl)­benzene-1-sulfonate (**11**)

White solid, yield: 88%, mp: 65–66 °C. IR (ν, cm^–1^): 3545, 3486, 3414 (N–H); 3087 (Ar–CH); 2915 (aliphatic CH); 1691 (C=O); 1572 (C=N stretching band); 1494, 1432 (Ar–C=C); 1384, 1171 (SO_2_); 1138, 1109 (Ar–C–CF_3_); 697 (Ar–CH). ^1^H NMR (400 MHz, DMSO-*d*_6_) δ ppm: 10.36 (*s*, 1H, N*H*), 8.72 (*s*, 1H, N=C*H*), 8.46 (*s*, 2H, Ar*H*), 7.79 (*d*, *J* = 9.1 Hz, 3H, Ar*H*), 7.17 (*d*, *J* = 8.6 Hz, 2H, Ar*H*), 6.56 (*s*, 2H, N*H*_2_). ^13^C NMR (101 MHz, DMSO-*d*_6_) δ ppm: 157.12 (*C*=O), 149.05 (*C*=N), 137.81, 137.17, 135.14, 132.52, 132.18, 129.55, 128.61, 122.87, 121.34 (Ar*C*). ESI-MS (*m*/*z*): 454 [M – H]^+^. Elemental analysis, C_16_H_11_F_6_N_3_O_4_S (455,33 g/mol). Found, %: C, 42.10; H, 2.39; N, 9.17; S, 7.10. Calculated, %: C, 42.21; H, 2.44; N, 9.23; S, 7.04. HPLC: purity 99%.

#### 3.3.12 4-[(*E*)-(2-Carbamoylhydrazinylidene)­methyl]­phenyl 2-(Trifluoromethoxy)­benzene-1-sulfonate (**12**)

White solid, yield: 92%, mp: 172–174 °C. IR (ν, cm^–1^): 3460, 3293 (N–H); 3065 (Ar–CH); 2988 (aliphatic CH); 1689 (C=O); 1596 (C=N stretching band); 1498, 1479 (Ar–C=C); 1382, 1194 (SO_2_); 1155, 1130 (Ar–C–OCF_3_); 694 (Ar–CH). ^1^H NMR (600 MHz, DMSO-*d*_6_) δ ppm: 10.35 (*s*, 1H, N*H*), 7.98 (*t*, *J* = 8.0 Hz, 1H, Ar*H*), 7.94 (*d*, *J* = 7.9 Hz, 1H, Ar*H*), 7.79 (*s*, 2H, Ar*H*), 7.76 (*d*, *J* = 8.6 Hz, 2H, Ar*H*), 7.62 (*t*, *J* = 7.7 Hz, 1H, Ar*H*), 7.04 (*d*, *J* = 8.5 Hz, 2H, Ar*H*), 6.54 (*s*, 2H, N*H*_2_). ^13^C NMR (151 MHz, DMSO-*d*_6_) δ ppm: 157.12 (*C*=O), 149.22 (*C*=N), 145.98, 138.27, 137.89, 134.90, 132.49, 128.64, 128.56, 127.16, 122.30, 122.06, 121.14 (Ar*C*). ESI-MS (*m*/*z*): 402 [M – H]^+^. Elemental analysis, C_15_H_12_F_3_N_3_O_5_S (403,33 g/mol). Found, %: C, 44.60; H, 3.05; N, 10.48; S, 8.00. Calculated, %: C, 44.67; H, 3.00; N, 10.42; S, 7.95. HPLC: purity 99%.

#### 3.3.13 4-[(*E*)-(2-Carbamoylhydrazinylidene)­methyl]­phenyl 3-(Trifluoromethoxy)­benzene-1-sulfonate (**13**)

White solid, yield: 90%, mp: 137–139 °C. IR (ν, cm^–1^): 3477, 3381 (N–H); 3082 (Ar–CH); 2987 (aliphatic CH); 1788 (C=O); 1587 (C=N stretching band); 1497, 1474 (Ar–C=C); 1378, 1173 (SO_2_); 1148, 1092 (Ar–C–OCF_3_); 692 (Ar–CH). ^1^H NMR (600 MHz, DMSO-*d*_6_) δ ppm: 10.35 (*s*, 1H, N*H*), 7.95–7.92 (*m*, 1H, Ar*H*), 7.88 (*s*, 1H, Ar*H*), 7.83 (*d*, *J* = 7.1 Hz, 2H, Ar*H*), 7.79 (*s*, 1H, N = C*H*), 7.76 (*d*, *J* = 8.6 Hz, 2H, Ar*H*), 7.05 (*d*, *J* = 8.6 Hz, 2H, Ar*H*), 6.55 (*s*, 2H, N*H*_2_). ^13^C NMR (151 MHz, DMSO-*d*_6_) δ ppm: 157.13 (*C*=O), 149.30 (*C*=N), 148.82, 137.93, 136.42, 134.88, 132.90, 131.89, 128.54, 128.40, 128.08, 122.67, 121.35 (Ar*C*). ESI-MS (*m*/*z*): 402 [M – H]^+^. Elemental analysis, C_15_H_12_F_3_N_3_O_5_S (403,33 g/mol). Found, %: C, 44.70; H, 2.95; N, 10.45; S, 7.90. Calculated, %: C, 44.67; H, 3.00; N, 10.42; S, 7.95. HPLC: purity 93%.

#### 3.3.14 4-[(*E*)-(2-Carbamoylhydrazinylidene)­methyl]­phenyl 4-(Trifluoromethoxy)­benzene-1-sulfonate (**14**)

White solid, yield: 90%, mp: 170–172 °C. IR (ν, cm^–1^): 3486, 3149 (N–H); 3069 (Ar–CH); 2972 (aliphatic CH); 1748 (C=O); 1576 (C=N stretching band); 1491, 1439 (Ar–C=C); 1363, 1198 (SO_2_); 1153, 1092 (Ar–C–OCF_3_); 702 (Ar–CH). ^1^H NMR (400 MHz, DMSO-*d*_6_) δ ppm: 10.34 (*s*, 1H, N*H*), 8.02 (*d*, *J* = 8.9 Hz, 2H, Ar*H*), 7.78 (*s*, 1H, N=C*H*), 7.75 (*d*, *J* = 8.7 Hz, 2H, Ar*H*), 7.66 (*d*, *J* = 8.4 Hz, 2H, Ar*H*), 7.04 (*d*, *J* = 8.6 Hz, 2H, Ar*H*), 6.54 (*s*, 2H, N*H*_2_). ^13^C NMR (101 MHz, DMSO-*d*_6_) δ ppm: 157.13 (*C*=O), 153.03, 149.33 (*C*=N), 137.90, 134.82, 133.29, 131.67, 128.55, 123.41, 122.74, 122.14 (Ar*C*). ESI-MS (*m*/*z*): 402 [M – H]^+^. Elemental analysis, C_15_H_12_F_3_N_3_O_5_S (403,33 g/mol). Found, %: C, 44.71; H, 3.07; N, 10.50; S, 8.01. Calculated, %: C, 44.67; H, 3.00; N, 10.42; S, 7.95. HPLC: purity 98%.

#### 3.3.15 4-[(*E*)-(2-Carbamoylhydrazinylidene)­methyl]­phenyl 4-Nitrobenzene-1-sulfonate (**15**)

White solid, yield: 95%, mp: 251–253 °C. IR (ν, cm^–1^): 3489 (N–H); 3075 (Ar–CH); 2988 (aliphatic CH); 1694 (C=O); 1576 (C=N stretching band); 1530, 1446 (Ar–C=C); 1351, 1149 (SO_2_); 679 (Ar–CH). ^1^H NMR (400 MHz, DMSO-*d*_6_) δ ppm: 10.34 (*s*, 1H, N*H*), 8.45 (*d*, *J* = 9.0 Hz, 2H, Ar*H*), 8.16 (*d*, *J* = 9.0 Hz, 2H, Ar*H*), 7.79 (*s*, 1H, N=C*H*), 7.75 (*d*, 2H, Ar*H*), 7.07 (*d*, *J* = 8.8 Hz, 2H, Ar*H*), 6.53 (*s*, 2H, N*H*_2_). ^13^C NMR (101 MHz, DMSO-*d*_6_) δ ppm: 157.18 (*C*=O), 151.54 (*C*=N), 149.12, 139.79, 137.83, 134.98, 130.58, 128.64, 125.49, 122.76 (Ar*C*). Elemental analysis, C_14_H_12_N_4_O_6_S (364,33 g/mol). Found, %: C, 46.21; H, 3.40; N, 15.40; S, 8.85. Calculated, %: C, 46.15; H, 3.32; N, 15.38; S, 8.80. HPLC: purity 99%.

#### 3.3.16 4-[(*E*)-(2-Carbamoylhydrazinylidene)­methyl]­phenyl [1,1′-Biphenyl]-4-sulfonate (**16**)

Off-white solid, yield: 89%, mp: 144–146 °C. IR (ν, cm^–1^): 3488 (N–H); 3091 (Ar–CH); 2923 (aliphatic CH); 1690 (C=O); 1573 (C=N stretching band); 1497, 1456 (Ar–C=C); 1373, 1150 (SO_2_); 671 (Ar–CH). ^1^H NMR (400 MHz, DMSO-*d*_6_) δ ppm: 10.32 (*s*, 1H, N*H*), 7.97 (*d*, *J* = 8.6 Hz, 2H, Ar*H*), 7.92 (*d*, *J* = 7.2 Hz, 2H, Ar*H*), 7.78 (*s*, 1H, N = C*H*), 7.74 (*d*, *J* = 7.3 Hz, 2H, Ar*H*), 7.57 – 7.46 (*m*, 5H, Ar*H*), 7.05 (*d*, *J* = 7.1 Hz, 2H, Ar*H*), 6.53 (*s*, 2H, N*H*_2_). ^13^C NMR (101 MHz, DMSO-*d*_6_) δ ppm: 157.16 (*C*=O), 149.53 (*C*=N), 146.69, 138.19, 138.01, 134.63, 133.27, 129.70, 129.54, 129.44, 128.50, 128.26, 127.69, 122.78 (Ar*C*) ESI-MS (*m*/*z*): 396 [M + H]^+^. Elemental analysis, C_20_H_17_N_3_O_4_S (395,43 g/mol). Found, %: C, 61.00; H, 4.36; N, 10.58; S, 8.15. Calculated, %: C, 60.75; H, 4.33; N, 10.63; S, 8.11. HPLC: purity 94%.

#### 3.3.17 4-[(*E*)-(2-Carbamoylhydrazinylidene)­methyl]­phenyl Naphthalene-1-sulfonate (**17**)

Brown solid, yield: 94%, mp: 88–90 °C. IR (ν, cm^–1^): 3486, 3454, 3320 (N–H); 3100 (Ar–CH); 2968 (aliphatic CH); 1708 (C=O); 1576 (C=N stretching band); 1497, 1444 (Ar–C=C); 1359, 1133 (SO_2_); 675 (Ar–CH). ^1^H NMR (400 MHz, DMSO-*d*_6_) δ ppm: 10.30 (*s*, 1H, N*H*), 8.67 (*d*, *J* = 8.8 Hz, 1H, Ar*H*), 8.43 (*d*, *J* = 8.4 Hz, 1H, Ar*H*), 8.23 (*d*, *J* = 8.2 Hz, 1H, Ar*H*), 8.10 (*d*, *J* = 7.3 Hz, 1H, Ar*H*), 7.94 (*t*, *J* = 7.8 Hz, 1H, Ar*H*), 7.81 (*t*, *J* = 7.5 Hz, 1H, Ar*H*), 7.71 (*s*, 1H, N= C*H*), 7.64 (*dd*, *J* = 8.2, 3.2 Hz, 3H, Ar*H*), 6.83 (*d*, *J* = 8.7 Hz, 2H, Ar*H*), 6.50 (*s*, 2H, N*H*_2_). ^13^C NMR (101 MHz, DMSO-*d*_6_) δ ppm: 157.09 (*C*=O), 149.48 (*C*=N), 137.80, 136.98, 134.63, 134.20, 132.03, 129.98, 128.45, 128.16, 128.03, 125.11, 124.53, 122.20 (Ar*C*). ESI-MS (*m*/*z*): 368 [M – H]^+^. Elemental analysis, C_18_H_15_N_3_O_4_S (369,40 g/mol). Found, %: C, 58.60; H, 4.11; N, 11.42; S, 8.71. Calculated, %: C, 58.53; H, 4.09; N, 11.38; S, 8.68. HPLC: purity 98%.

#### 3.3.18 4-[(*E*)-(2-Carbamoylhydrazinylidene)­methyl]­phenyl Naphthalene-2-sulfonate (**18**)

Yellow solid, yield: 90%, mp: 81–83 °C. IR (ν, cm^–1^): 3484 (N–H); 3100 (Ar–CH); 2968 (aliphatic CH); 1688­(C=O); 1573 (C=N stretching band); 1497, 1457 (Ar–C=C); 1372, 1152 (SO_2_); 659 (Ar–CH). ^1^H NMR (400 MHz, DMSO-*d*_6_) δ ppm: 10.30 (*s*, 1H, N*H*), 8.59 (*s*, 1H, Ar*H*), 8.23 (*d*, *J* = 8.8 Hz, 2H, Ar*H*), 8.13 (*d*, *J* = 8.4 Hz, 1H, Ar*H*), 7.90 (*d*, *J* = 8.8 Hz, 1H, Ar*H*), 7.82–7.77 (m, 1H, Ar*H*), 7.75 (*s*, 1H, N = C*H*), 7.73–7.68 (*m*, 3H, Ar*H*), 7.02 (*d*, *J* = 8.7 Hz, 2H, Ar*H*), 6.51 (*s*, 2H, N*H*_2_). ^13^C NMR (101 MHz, DMSO-*d*_6_) δ ppm: 157.12 (*C*=O), 149.56 (*C*=N), 137.93, 135.55, 134.60, 131.89, 131.63, 130.85, 130.53, 130.13, 128.61, 128.47, 123.01, 122.75 (Ar*C*). ESI-MS (*m*/*z*): 368 [M – H]^+^. Elemental analysis, C_18_H_15_N_3_O_4_S (369,40 g/mol). Found, %: C, 58.60; H, 4.06; N, 11.41; S, 8.70. Calculated, %: C, 58.53; H, 4.09; N, 11.38; S, 8.68. HPLC: purity 99%.

### 3.4 DFT Studies

Quantum chemical investigations were performed using density functional theory (DFT) as implemented in the Gaussian 09 software suite.^51^ Compounds with good enzyme activity (**12**, **17**, and **18**) were optimized, and vibrational frequency analyses were carried out to verify that these structures represent true local minima on the potential energy surface. The B3LYP functional combined with the 6–311++G­(d,p) basis set was employed for all calculations.^51–54^ To better understand the electronic distribution and reactive tendencies of the molecules, frontier molecular orbitals (FMOs) and molecular electrostatic potential (MEP) maps were generated. The energies of the highest occupied molecular orbital (HOMO) and the lowest unoccupied molecular orbital (LUMO) served as the basis for calculating various global reactivity descriptors (GRPs) (eq 1). These parameters include

1 $$\begin{array}{ll} I=-E_{\mathrm{HOMO}} & \left(1\right)\\ A=-E_{\mathrm{LUMO}} & \left(2\right)\\ \chi =-\left[\frac{E_{\mathrm{LUMO}}+E_{\mathrm{HOMO}}}{2}\right] & \left(3\right)\\ \chi =\frac{\left(I-A\right)}{2} & \left(4\right)\\ \mu =-\chi & \left(5\right)\\ \sigma =\frac{1}{\eta } & \left(6\right)\\ \omega =\mu^{2}/2\eta & \left(7\right) \end{array}$$

### 3.5 *In Vitro* Colinesterase Inhibition Studies

The semicarbazone derivatives were evaluated for their inhibitory activities against human acetylcholinesterase (AChE) and butyrylcholinesterase (BChE) enzymes using standard *in vitro* assays. The Ellman’s technique that was tailored to our lab’s needs was used to measure the activity of the cholinesterase enzyme.^55^ The test chemicals were first dissolved in DMSO at a concentration of 1 mg/mL to create stock solutions, which were then diluted 10 times with distilled water for use in the inhibition tests. Each compound’s inhibitory effects on the enzymes were evaluated at five different concentrations. Fifty microliters of distilled water, 50 μL of 0.1 M Tris–HCl buffer (pH 8.0), 15 μL of 0.015 M acetylcholine iodide or butyrylcholine iodide (as substrates), 30 μL of 0.06 M DTNB (5,5′-dithiobis­(2-nitrobenzoic acid)), and 10 μL of enzyme solution made up the reaction mixture for the AChE and BChE tests. The absorbance of the blank solutions was measured before the substrate was added. The substrate was then added to each well to start the enzymatic reaction. Enzyme activity was kinetically assessed at 408 nm by using a microplate reader. Each inhibition measurement was repeated three times, and average values were taken when measuring the inhibitory potentials of the molecules. The IC_50_ values, indicating each compound’s inhibitory strength against cholinesterase, were determined by plotting percent activity *versus* inhibitor concentration.

### 3.6 Enzyme Kinetics

To determine the inhibition type of BChE, kinetic studies were performed with the most active compounds (**12**, **17**, and **18**). Different concentrations of butyrylthiocholine iodide were used in the presence of three concentrations of each compound. Lineweaver–Burk plots were constructed by plotting the reciprocal of reaction velocity (1/V) against the reciprocal of substrate concentration (1/[S]). Based on these plots, the inhibition types were determined. The inhibition constant (*K*_i_) was calculated using the following equation (eq 2).

2 $$\mathrm{slope}=K_{m}/V_{\max}\times \left(1+\left[I\right]/K_{i}\right)$$

### 3.7 Molecular Docking

Molecular docking studies were conducted to elucidate the interactions between the synthesized compounds and the enzyme butyrylcholinesterase (BChE) at the atomic level. The docking simulations were carried out using Molegro Virtual Docker (MVD) 6.0.^56^ The crystal structure of BChE was obtained from the Protein Data Bank (4BDS).^57^ The MVD software automatically performed protein and ligand preparation, removal of water molecules, and automatic identification of the enzyme’s active site. The binding region targeted for docking corresponds to the site where a known reference ligand interacts. Each compound underwent 15 independent docking runs, and the best binding poses were selected based on MolDock scoring. Interaction details and 2D binding visualizations were further examined using BIOVIA Discovery Studio.^58^

### 3.8 Molecular Dynamic Simulation and Enzyme Preparation

Molecular dynamics (MD) simulations were conducted using GROMACS 2024,^59^ leveraging the VSmartMD tool^60^ for automated execution and visualization. Initial.pdb files of protein–ligand complexes were prepared, and simulations were performed with the CHARMM27 force field. Protein topologies were derived *via* pdb 2gmx, while ligand parameters were assigned using the SwissParam server.

The systems were solvated in a TIP3P water model within a cubic box, maintaining a 1 nm buffer between the protein and the box edges under periodic boundary conditions. Charge neutrality was achieved by adding Na^+^ ions, followed by energy minimization (50,000 steepest descent steps). Equilibration included NVT (100 ps, 300 K) and NPT (100 ps, 1 bar) phases, with temperature/pressure regulated *via* the Berendsen method (time constants: 0.1 ps for temperature and 2 ps for pressure). The Leapfrog integrator was used with separate coupling for protein, ligand, solvent, and ions.

Production runs spanned 100 ns under NPT conditions (1 ps pressure coupling), with LINCS-constrained bonds, a 1.2 nm cutoff for nonbonded interactions, and PME for long-range electrostatics. Trajectories were analyzed using VMD 1.9.2,^61^ HeroMDAnalysis,^62^ and VSmartMD.^60^

### 3.9 ADMET

ADME studies evaluate a compound’s absorption, distribution, metabolism, and excretion within the body through the application of predictive mathematical models. Since pharmacodynamic and pharmacokinetic properties are critical in drug development, such assessments play a vital role in guiding early-stage drug design. *In silico* ADMET analysis offers insight into a molecule’s drug-likeness and suitability as a therapeutic agent, contributing to more cost-effective and time-efficient drug discovery. In this study, the ADMET profiles of all synthesized compounds were predicted using the SwissADME web tool,^63^ while toxicity-related parameters were assessed with the ProTox-II platform.^64^

In this study, the ADMET profiles of all synthesized compounds were predicted using the SWISS ADME,^63^ ProTox-II,^64^ and PKCSM^65^ online sites.

## 4 Conclusion

In this study, a novel series of 18 (*E*)-4-((2-carbamoylhydrazinylidene)­methyl)­phenyl-substituted sulfonate derivatives (**1–18**) were successfully synthesized in high yields (93–98%) and structurally characterized by FT-IR, ^1^H NMR, and ^13^C NMR spectroscopy. *In vitro* inhibitory studies against BChE revealed that several compounds, particularly **11**, **12**, **13**, **14**, **16**, **17**, and **18**, exhibited stronger inhibition than the reference drug pyridostigmine bromide. Among them, compound 12 emerged as the most potent inhibitor (IC_50_ = 61.88 μM). Molecular dynamics and binding energy analyses were conducted to evaluate the inhibitory effectiveness of “Compounds **12**, **17**, and **18**” on the 4DBS protein, using “tacrine” as a reference ligand. The RMSD and RMSF findings indicated that all ligands preserved the structural integrity of the protein with “Compound **17**” and “tacrine” showing the smallest conformational fluctuations. Crucial binding site residues such as “*THR120*” and “*TYR440*” remained consistently involved across all complexes, with “Compound **17**” demonstrating the highest occupancy of hydrogen bonds at these positions. Additionally, “Compound **18**” recorded the highest average number of hydrogen bond interactions. Binding free energy results identified “Compound **17**” as having the most favorable interaction energy (−54.74 kJ/mol), exceeding that of tacrine. Overall, these findings highlight “compound **17”** as the most promising BChE inhibitor candidate, with “compound **18”** also demonstrating strong and stable binding characteristics, thereby supporting the potential of this newly synthesized semicarbazone series as lead scaffolds for further drug development.

## Supplementary Material



## Notes

1H NMR, ^13^C NMR, FT-IR, and Mass spectrum of compounds **A8, A12–13, and 1–18** (PDF)
