# Supplementary material for: New Sulfonate-Semicarbazone Hybrid Molecules: Synthesis, Theoretical Evaluations, Molecular Simulations, and Butyrylcholinesterase Inhibition Activity
Source: ACS Omega. 2025 Dec 26;11(1):1727–44. doi: 10.1021/acsomega.5c09763 (PMC12809313; doi:10.1021/acsomega.5c09763)
Supplement: Supplementary file 1 [file ao5c09763_si_001.pdf]

SUPPORTING INFORMATION FOR PUBLICATION

Title: New Sulfonate-Semicarbazone Hybrid Molecules: Synthesis, Theoretical Evaluations, Molecular Simulations, and Butyrylcholinesterase Inhibition Activity

Bedriye Seda Kurşun-Aktar[a,*], Emine Elçin Oruç-Emre[b], Zafer Bulut[b], Emel Ekinci[c], Volkan Eyüpoğlu[d], Şevki Adem[d], Ayşegül Karaküçük-İyidoğan[b]

[a]Department of Engineering Basic Sciences, Faculty of Engineering and Natural Sciences, Malatya Turgut Özal University, Battalgazi, Malatya 44210, Türkiye

[b]Department of Chemistry, Faculty of Arts and Sciences, Gaziantep University, Gaziantep 27310, Türkiye

[c]Çankırı Karatekin University, Central Research Laboratory Application and Research Center, Çankırı 18100, Türkiye

[d]Çankırı Karatekin University, Faculty of Science, Department of Chemistry, Çankırı 18100, Türkiye

***Correspondence Author:**

Bedriye Seda Kurşun Aktar

Department of Engineering Basic Sciences, Faculty of Engineering and Natural Sciences, Malatya Turgut Özal University, Battalgazi, Malatya, Türkiye

Phone number: +90 252 211 32 58

Fax number: +90 252 211 50 41

E-mail: bseda.kursunaktar@ozal.edu.tr

INDEX

Figure S1. ^{1}H NMR spectrum of A8 4
Figure S2. ^{1}H NMR spectrum of A12 4
Figure S3. ^{1}H NMR spectrum of A13 5
Figure S4. FT-IR spectrum of 1 5
Figure S5. ^{1}H NMR spectrum of 1 6
Figure S6. ^{13}C NMR spectrum of 1 6
Figure S7. Mass spectrum of 1 7
Figure S8. FT-IR spectrum of 2 7
Figure S9. ^{1}H NMR spectrum of 2 8
Figure S10. ^{13}C NMR spectrum of 2 8
Figure S11. Mass spectrum of 2 9
Figure S12. FT-IR spectrum of 3 9
Figure S13. ^{1}H NMR spectrum of 3 10
Figure S14. ^{13}C NMR spectrum of 3 10
Figure S15. Mass spectrum of 3 11
Figure S16. FT-IR spectrum of 4 11
Figure S17. ^{1}H NMR spectrum of 4 12
Figure S18. ^{13}C NMR spectrum of 4 12
Figure S19. Mass spectrum of 4 13
Figure S20. FT-IR spectrum of 5 13
Figure S21. ^{1}H NMR spectrum of 5 14
Figure S22. ^{13}C NMR spectrum of 5 14
Figure S23. Mass spectrum of 5 15
Figure S24. FT-IR spectrum of 6 15
Figure S25. ^{1}H NMR spectrum of 6 16
Figure S26. ^{13}C NMR spectrum of 6 16
Figure S27. Mass spectrum of 6 17
Figure S28. FT-IR spectrum of 7 17
Figure S29. ^{1}H NMR spectrum of 7 18
Figure S30. ^{13}C NMR spectrum of 7 18
Figure S31. Mass spectrum of 7 19
Figure S32. FT-IR spectrum of 8 19
Figure S33. ^{1}H NMR spectrum of 8 20
Figure S34. ^{13}C NMR spectrum of 8 20
Figure S35. Mass spectrum of 8 21
Figure S36. FT-IR spectrum of 9 21
Figure S37. ^{1}H NMR spectrum of 9 22
Figure S38. ^{13}C NMR spectrum of 9 22
Figure S39. Mass spectrum of 9 23
Figure S40. FT-IR spectrum of 10 23
Figure S41. ^{1}H NMR spectrum of 10 24
Figure S42. ^{13}C NMR spectrum of 10 24
Figure S43. Mass spectrum of 10 25
Figure S44. FT-IR spectrum of 11 25
Figure S45. ^{1}H NMR spectrum of 11 26
Figure S46. ^{13}C NMR spectrum of 11 26
Figure S47. Mass spectrum of 11 27
Figure S48. FT-IR spectrum of 12 27
Figure S49. ^{1}H NMR spectrum of 12 28

Figure S50. ^{13}C NMR spectrum of 12 28
Figure S51. Mass spectrum of 12 29
Figure S52. FT-IR spectrum of 13 29
Figure S53. ^{1}H NMR spectrum of 13 30
Figure S54. ^{13}C NMR spectrum of 13 30
Figure S55. Mass spectrum of 13 31
Figure S56. FT-IR spectrum of 14 31
Figure S57. ^{1}H NMR spectrum of 14 32
Figure S58. ^{13}C NMR spectrum of 14 32
Figure S59. Mass spectrum of 14 33
Figure S60. FT-IR spectrum of 15 33
Figure S61. ^{1}H NMR spectrum of 15 34
Figure S62. ^{13}C NMR spectrum of 15 34
Figure S63. Mass spectrum of 15 35
Figure S64. FT-IR spectrum of 16 35
Figure S65. ^{1}H NMR spectrum of 16 36
Figure S66. ^{13}C NMR spectrum of 16 36
Figure S67. Mass spectrum of 16 37
Figure S68. FT-IR spectrum of 17 37
Figure S69. ^{1}H NMR spectrum of 17 38
Figure S70. ^{13}C NMR spectrum of 17 38
Figure S71. Mass spectrum of 17 39
Figure S72. FT-IR spectrum of 18 39
Figure S73. ^{1}H NMR spectrum of 18 40
Figure S74. ^{13}C NMR spectrum of 18 40
Figure S75. Mass spectrum of 18 41
Figure S76. HPLC chromatogram of 1 41
Figure S77. HPLC chromatogram of 2 42
Figure S78. HPLC chromatogram of 3 43
Figure S79. HPLC chromatogram of 4 44
Figure S80. HPLC chromatogram of 5 45
Figure S81. HPLC chromatogram of 6 46
Figure S82. HPLC chromatogram of 7 47
Figure S83. HPLC chromatogram of 8 48
Figure S84. HPLC chromatogram of 9 49
Figure S85. HPLC chromatogram of 10 50
Figure S86. HPLC chromatogram of 11 51
Figure S87. HPLC chromatogram of 12 52
Figure S88. HPLC chromatogram of 13 53
Figure S89. HPLC chromatogram of 14 54
Figure S90. HPLC chromatogram of 15 55
Figure S91. HPLC chromatogram of 16 56
Figure S92. HPLC chromatogram of 17 57
Figure S93. HPLC chromatogram of 18 58

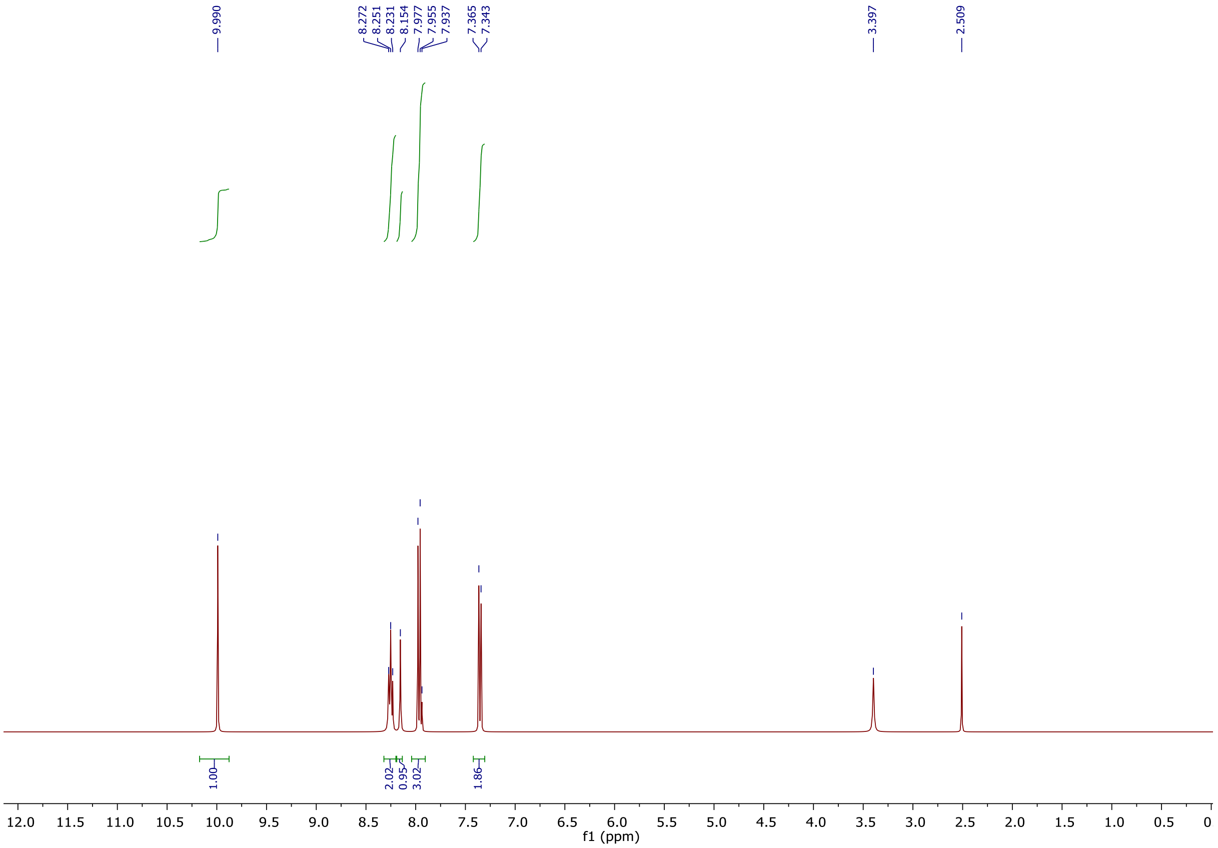

Figure S1. ^{1}H NMR spectrum of A8

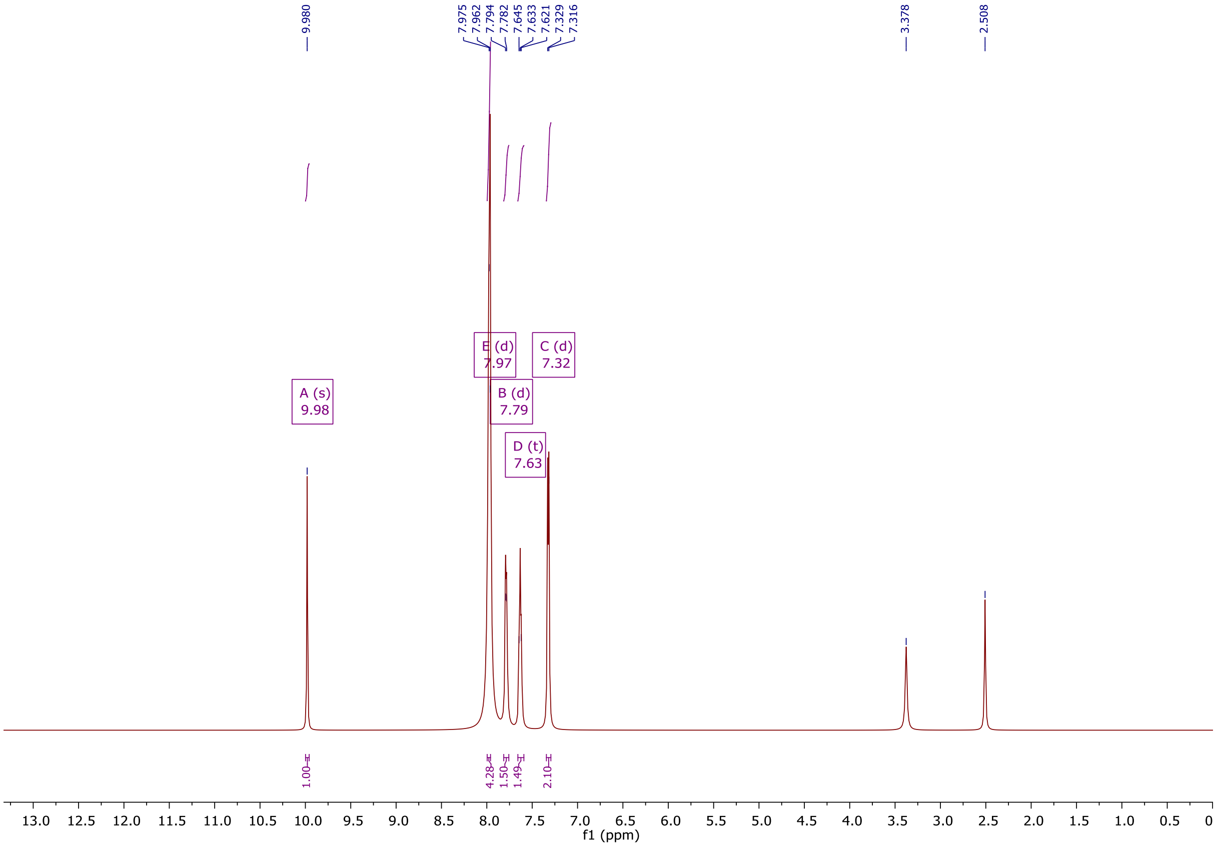

Figure S2. ^{1}H NMR spectrum of A12

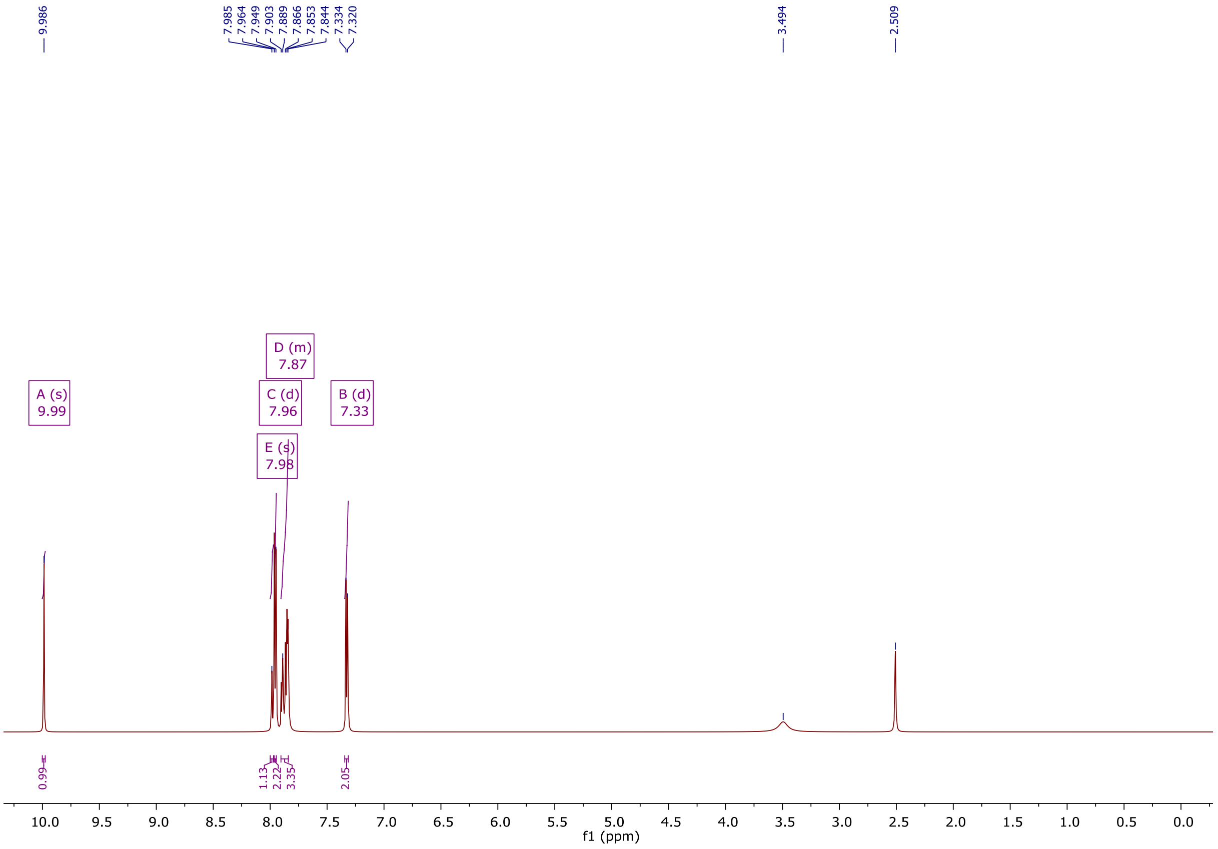

Figure S3. ^{1}H NMR spectrum of A13

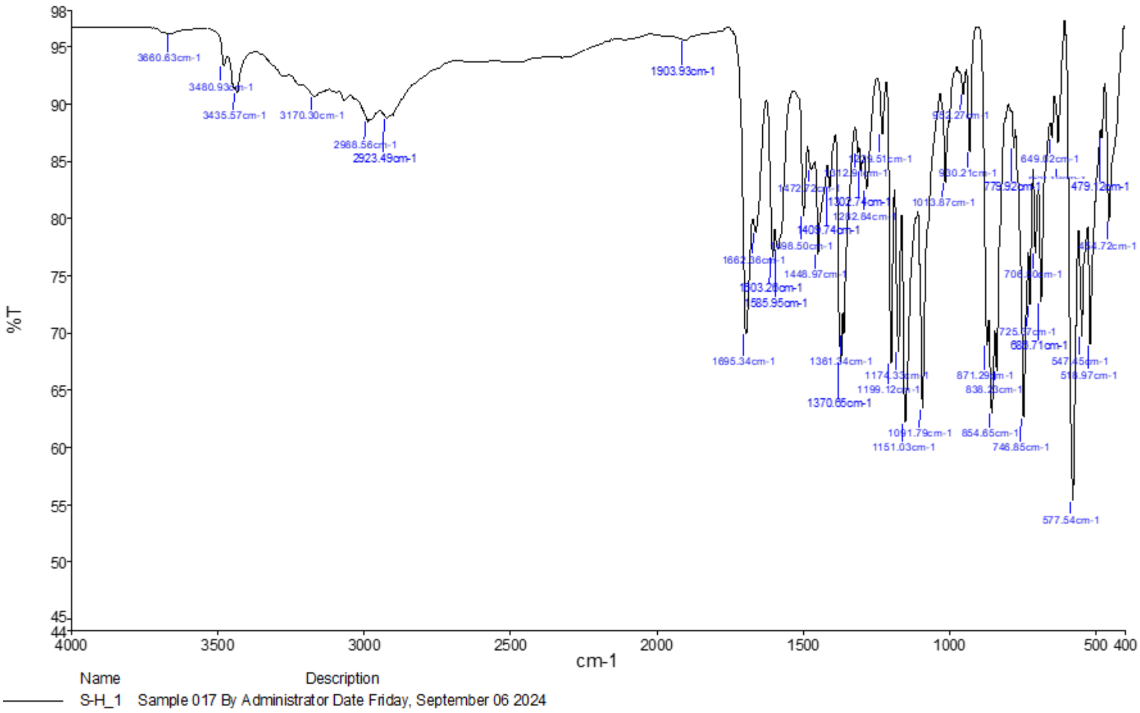

Figure S4. FT-IR spectrum of 1

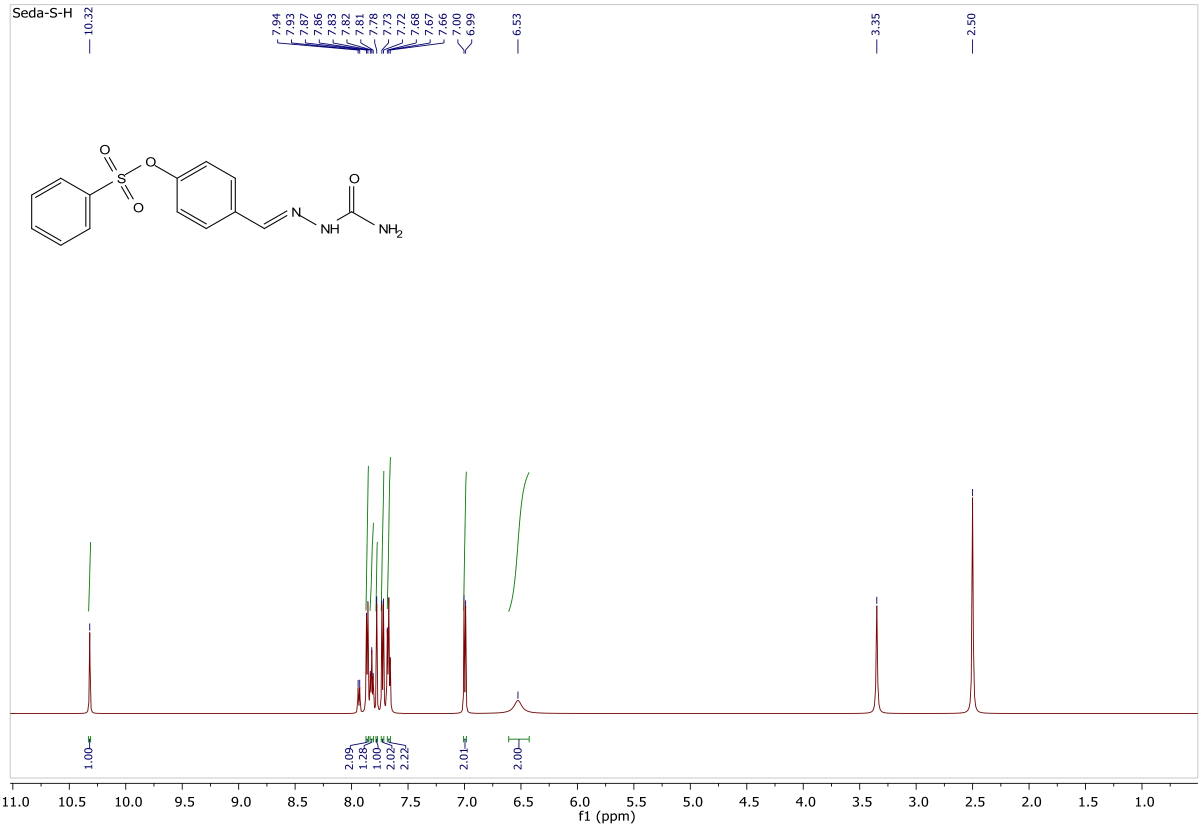

Figure S5. ^{1}H NMR spectrum of 1

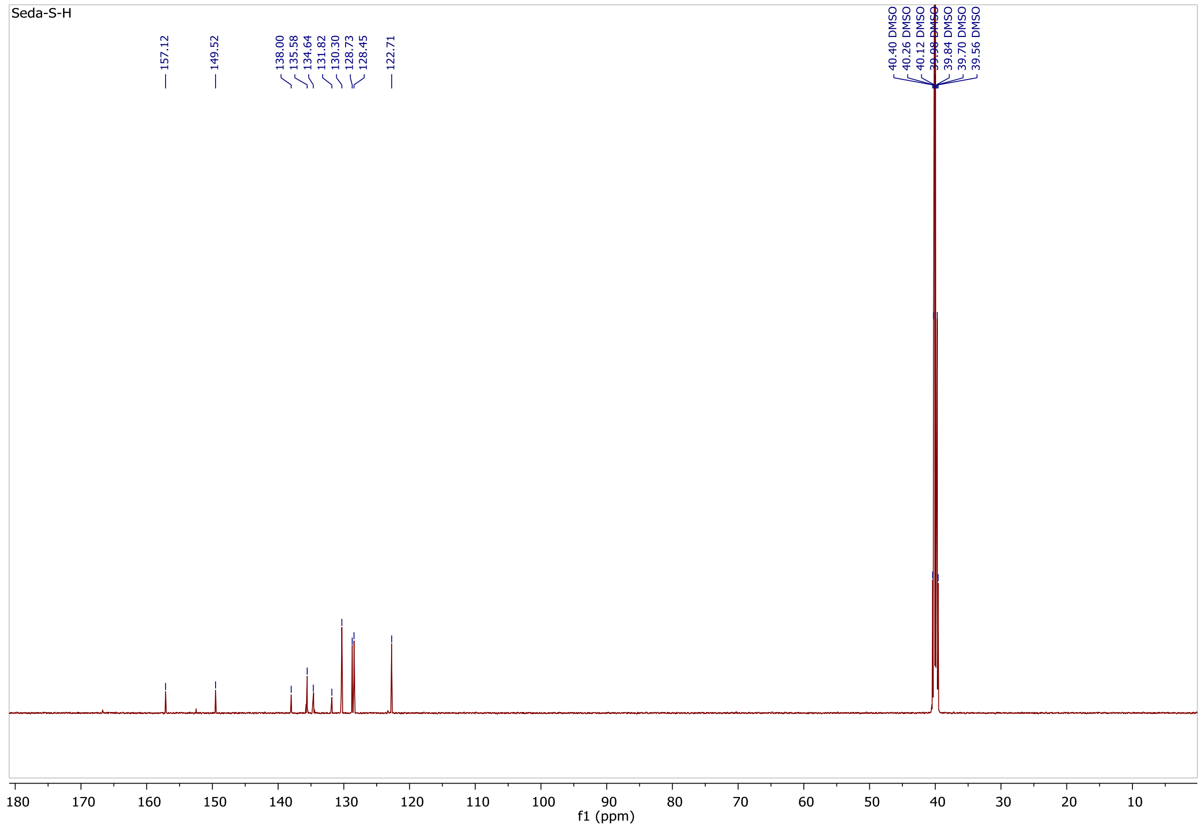

Figure S6. ^{13}C NMR spectrum of 1

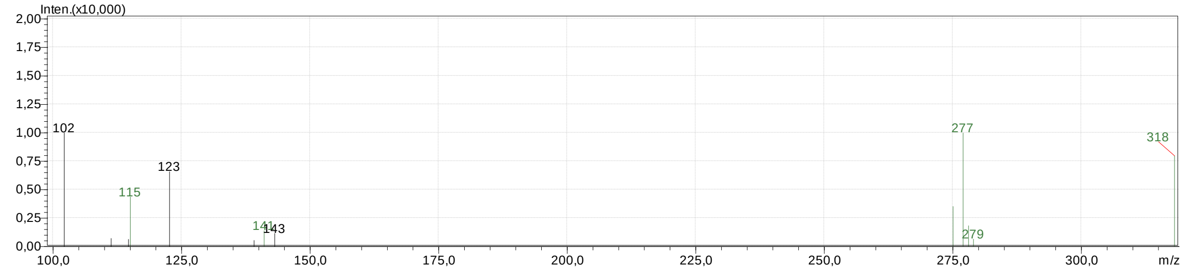

Figure S7. Mass spectrum of 1

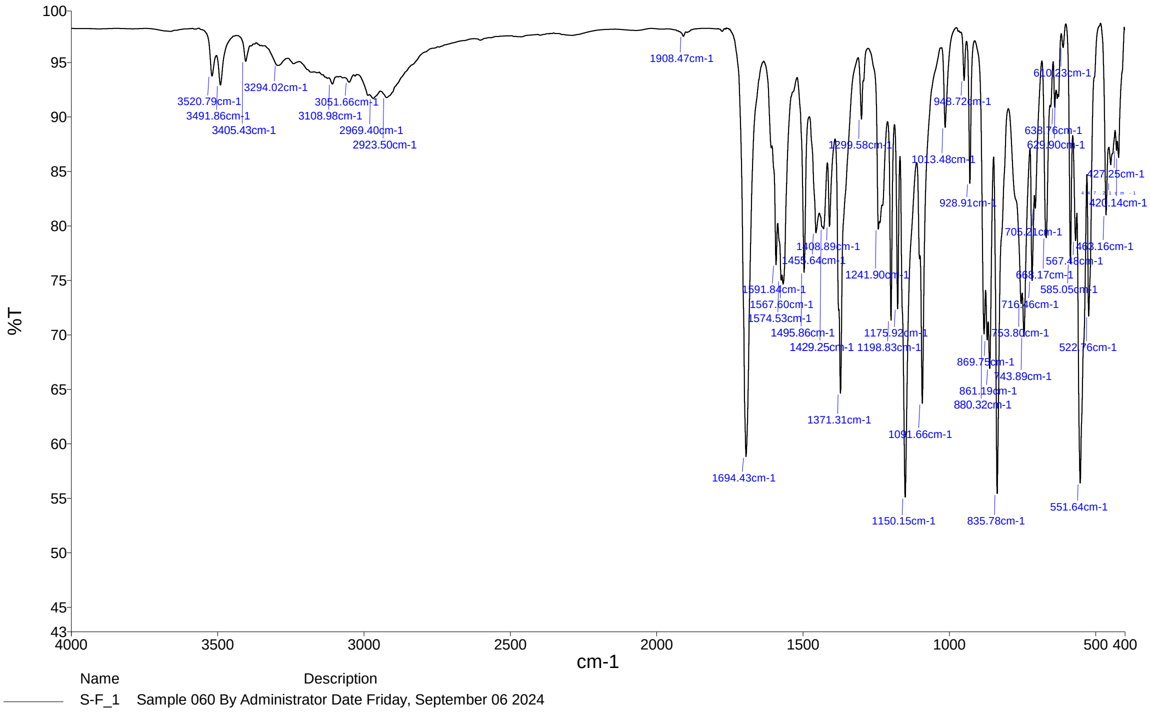

Figure S8. FT-IR spectrum of 2

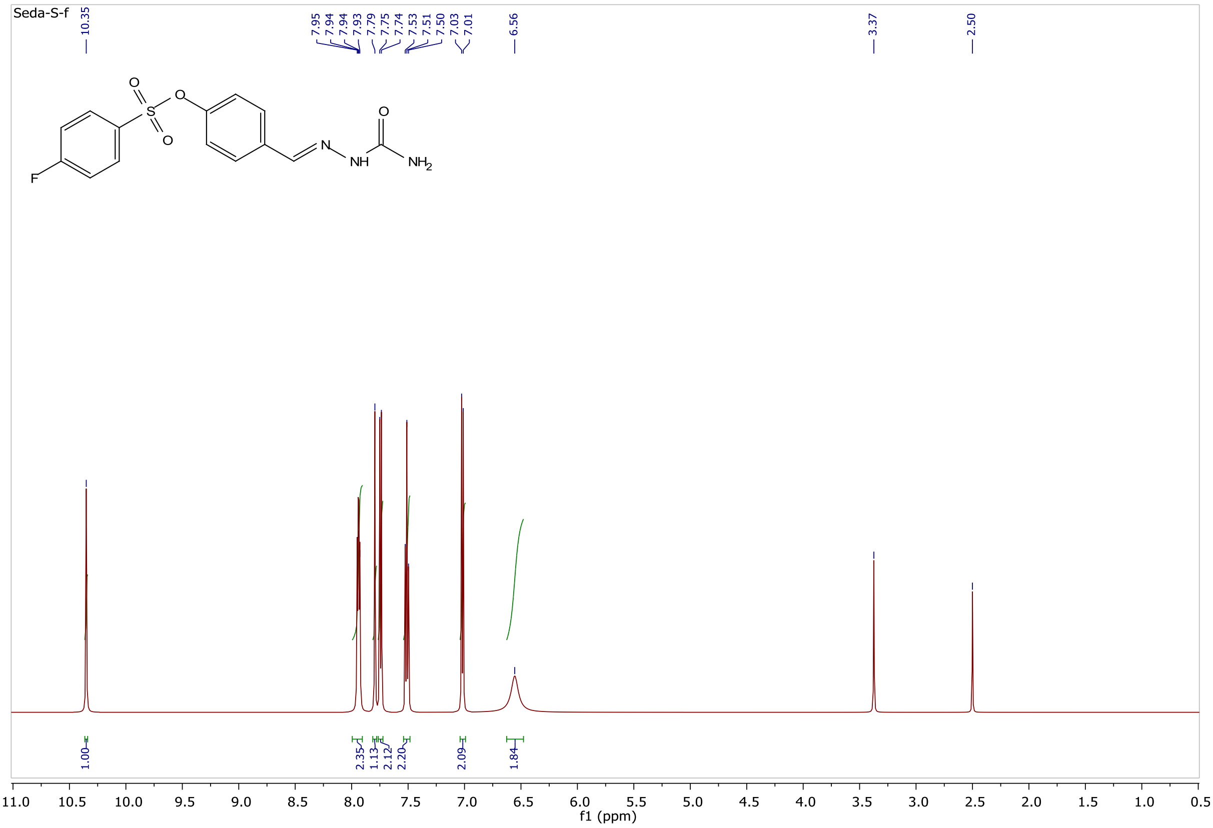

Figure S9. ^{1}H NMR spectrum of 2

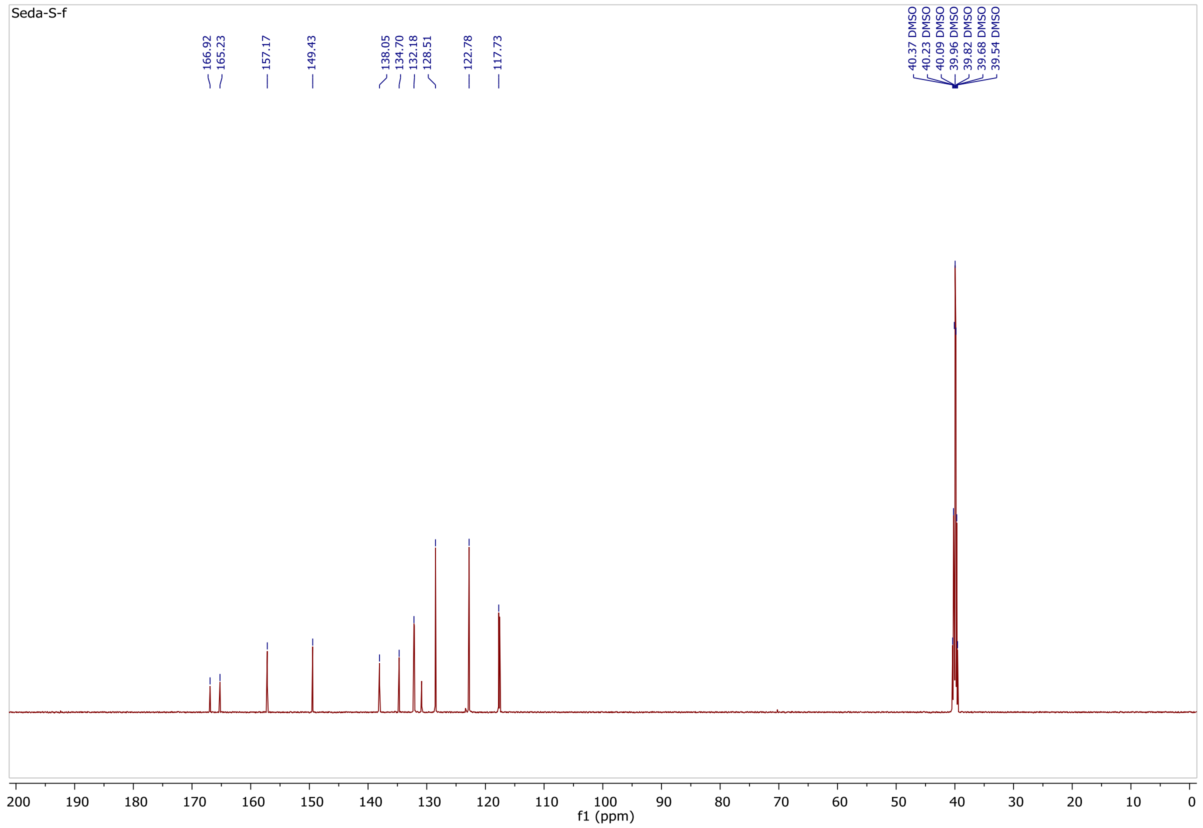

Figure S10. ^{13}C NMR spectrum of 2

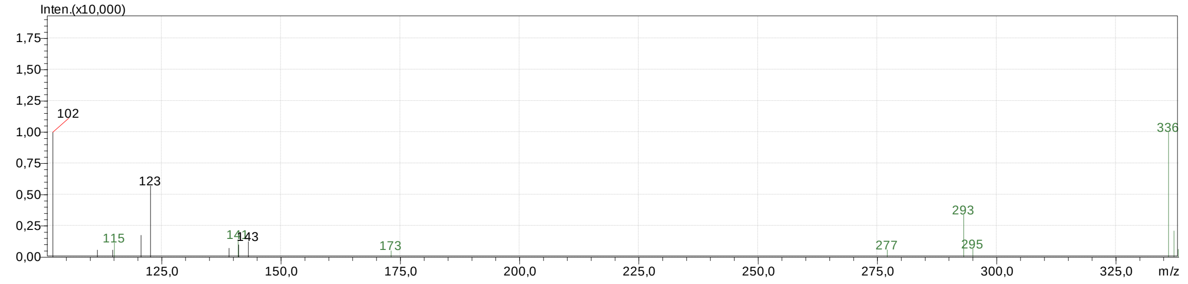

Figure S11. Mass spectrum of 2

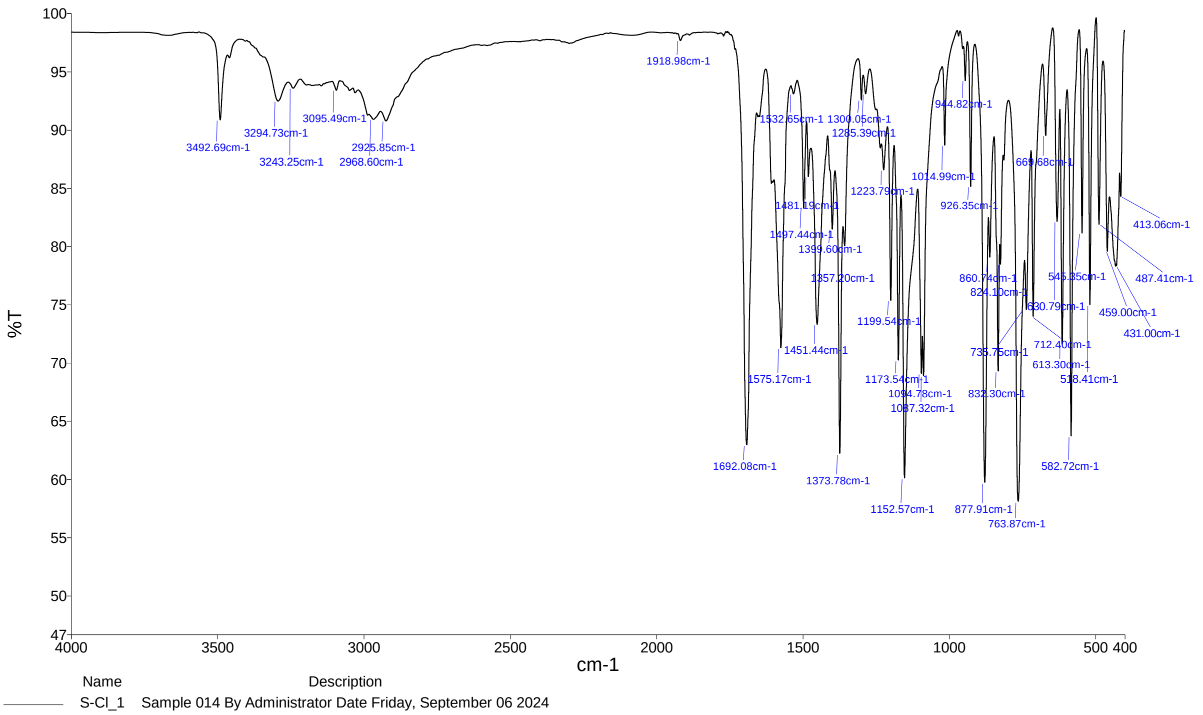

Figure S12. FT-IR spectrum of 3

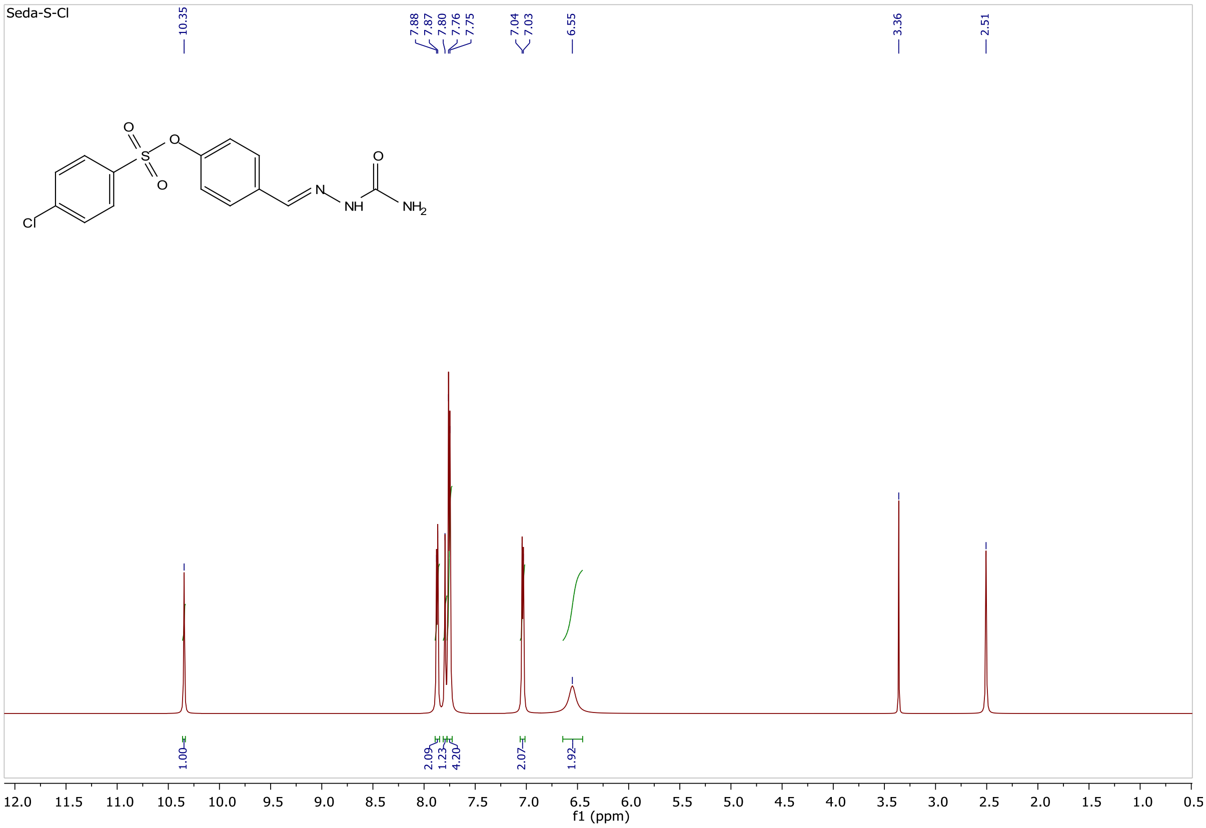

Figure S13. ^{1}H NMR spectrum of 3

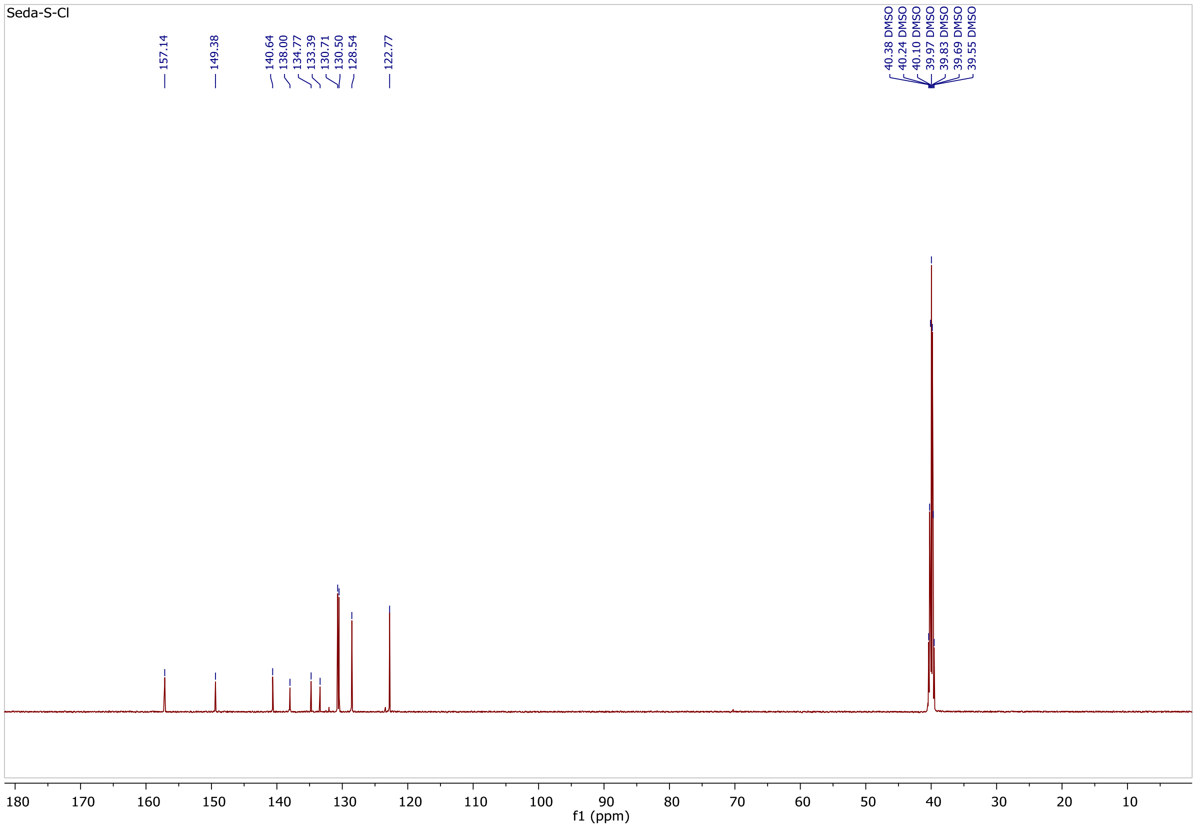

Figure S14. ^{13}C NMR spectrum of 3

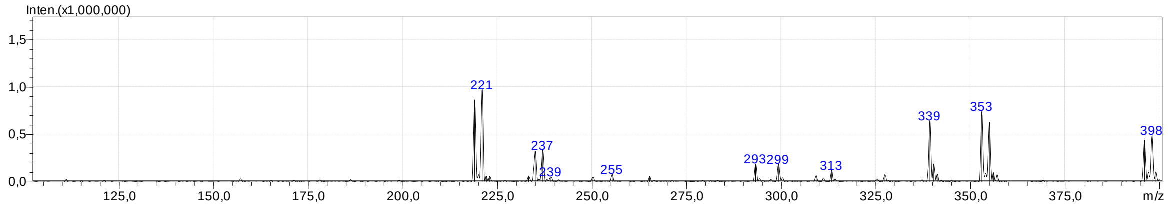

Figure S15. Mass spectrum of 3

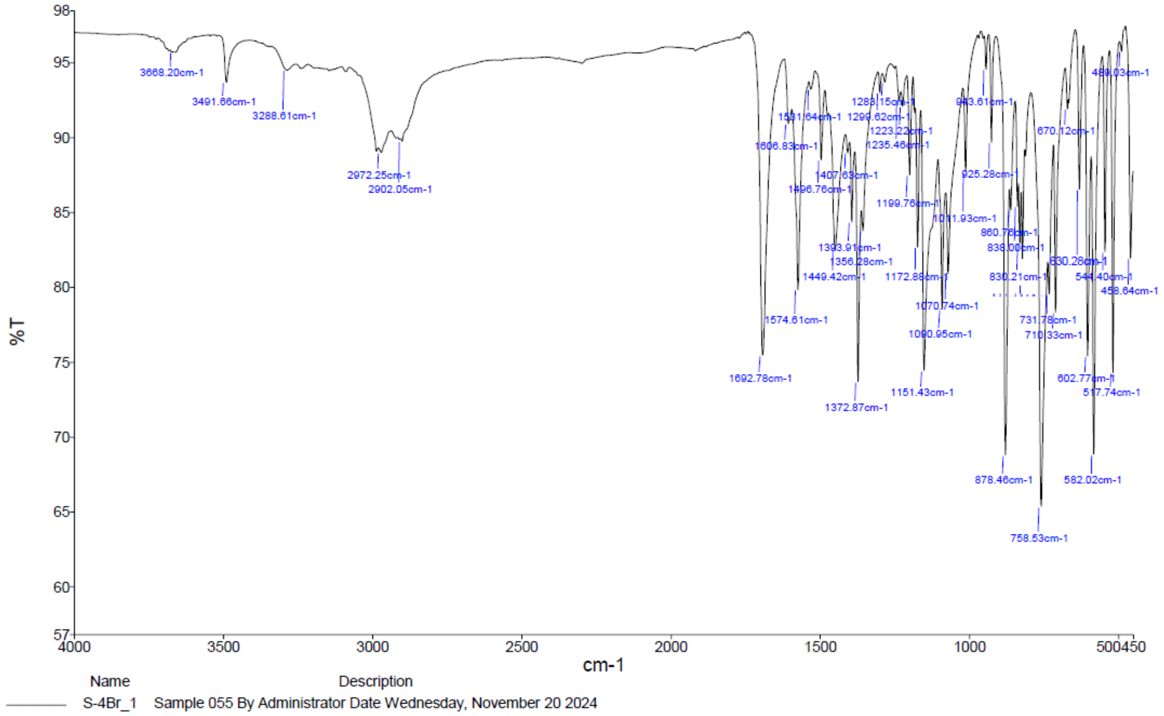

Figure S16. FT-IR spectrum of 4

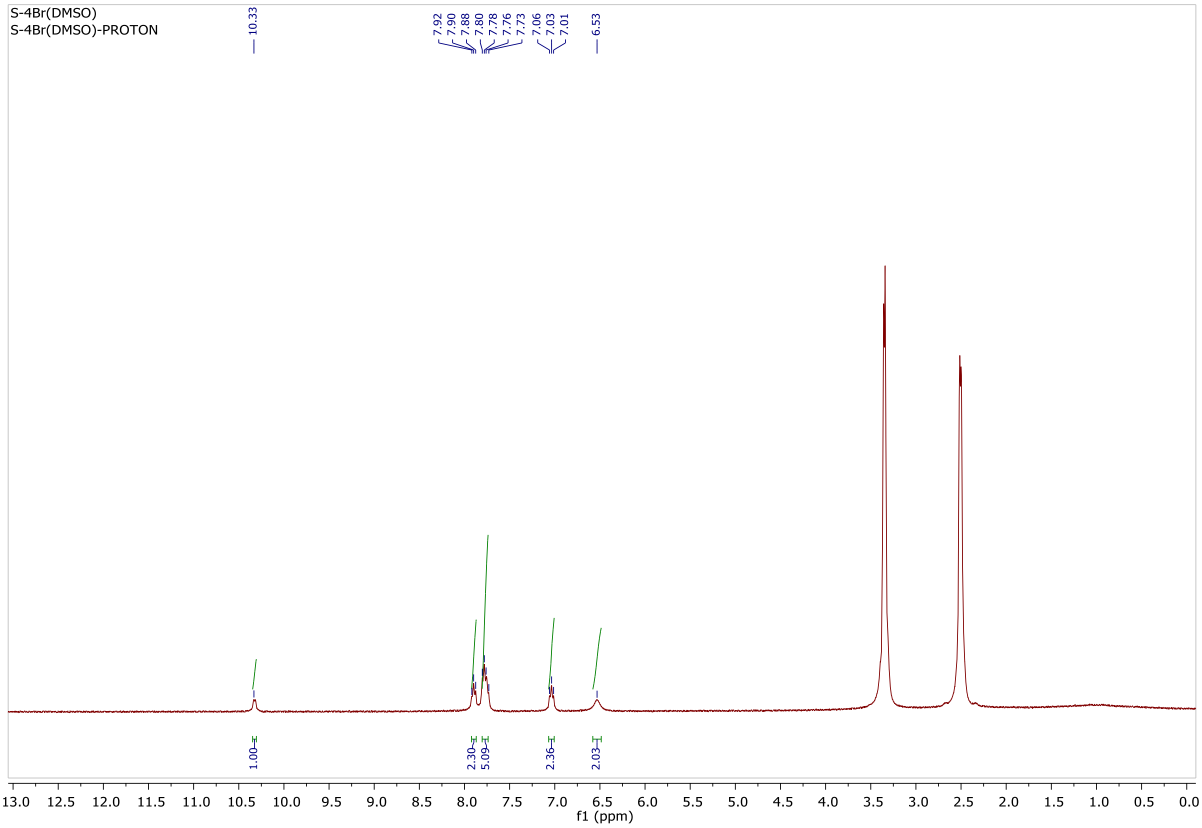

Figure S17. ^{1}H NMR spectrum of 4

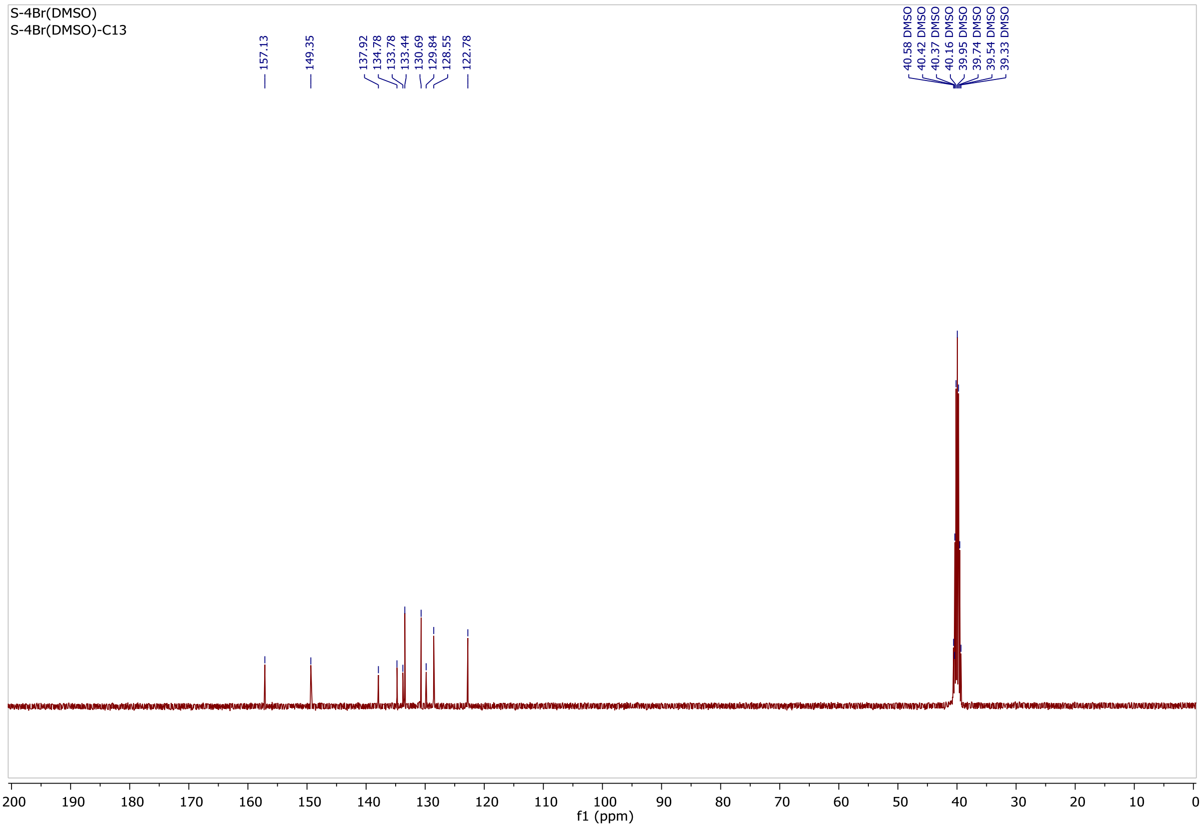

Figure S18. ^{13}C NMR spectrum of 4

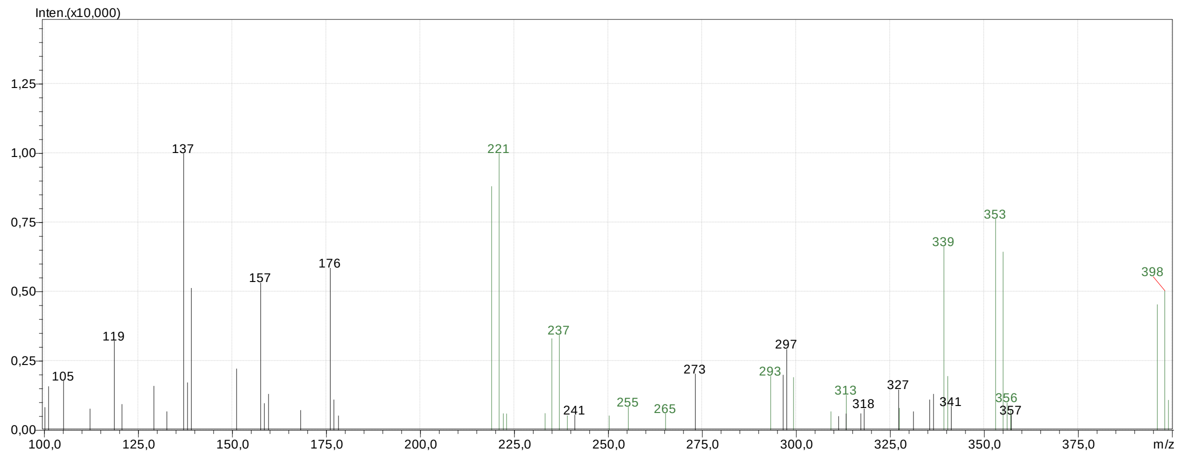

Figure S19. Mass spectrum of 4

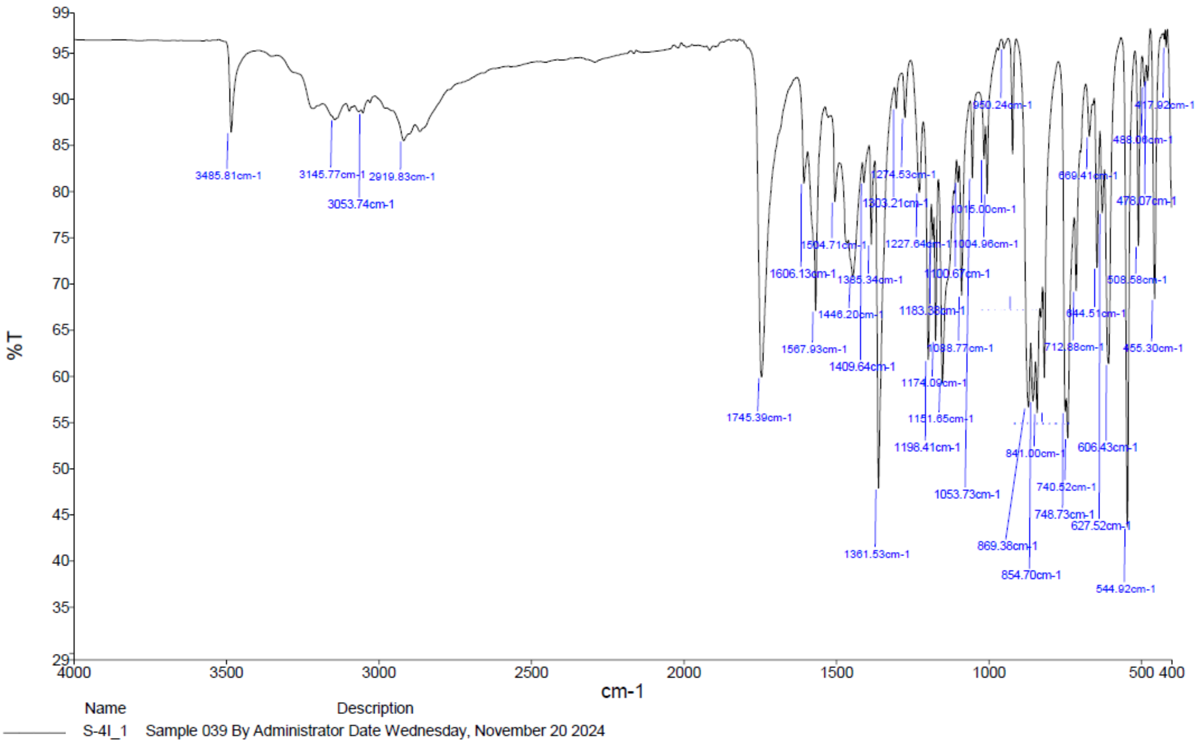

Figure S20. FT-IR spectrum of 5

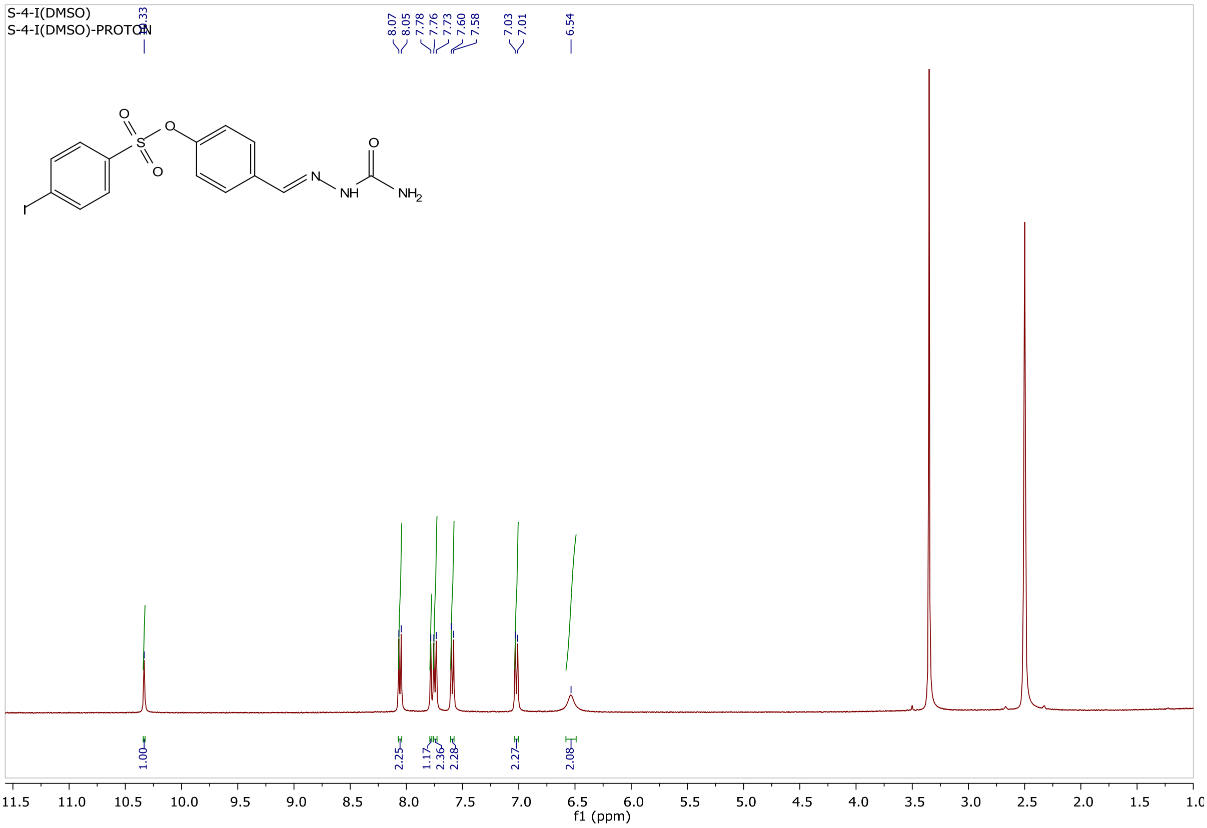

Figure S21. ^{1}**H NMR spectrum of 5**

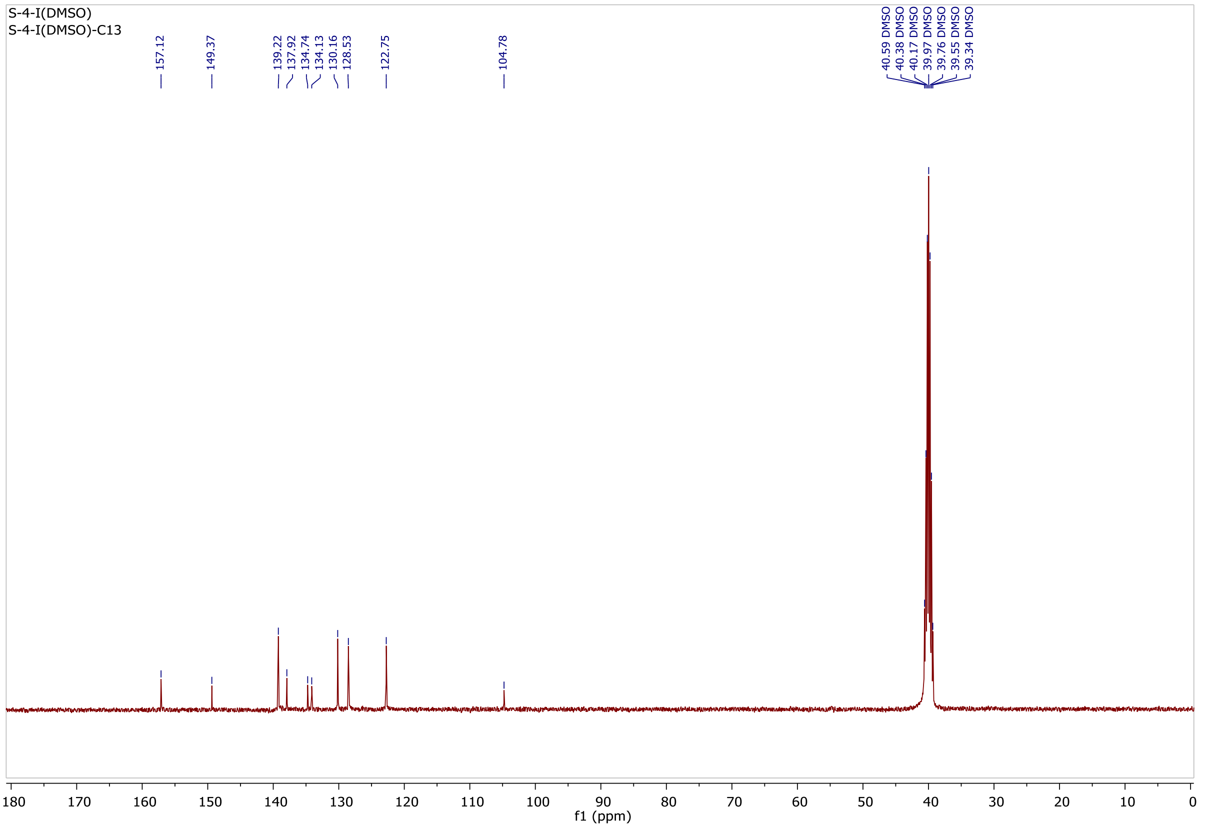

Figure S22. ^{13}**C NMR spectrum of 5**

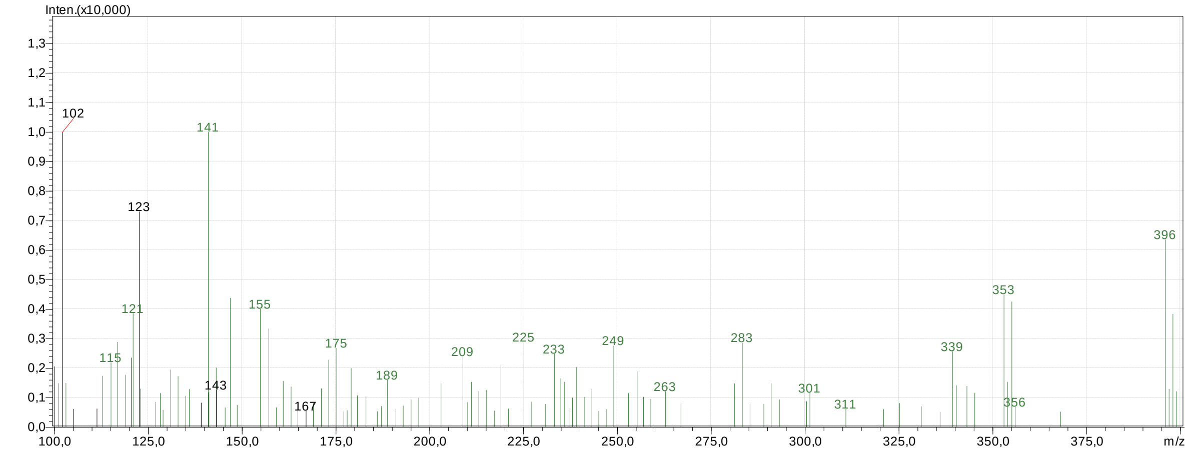

Figure S23. Mass spectrum of 5

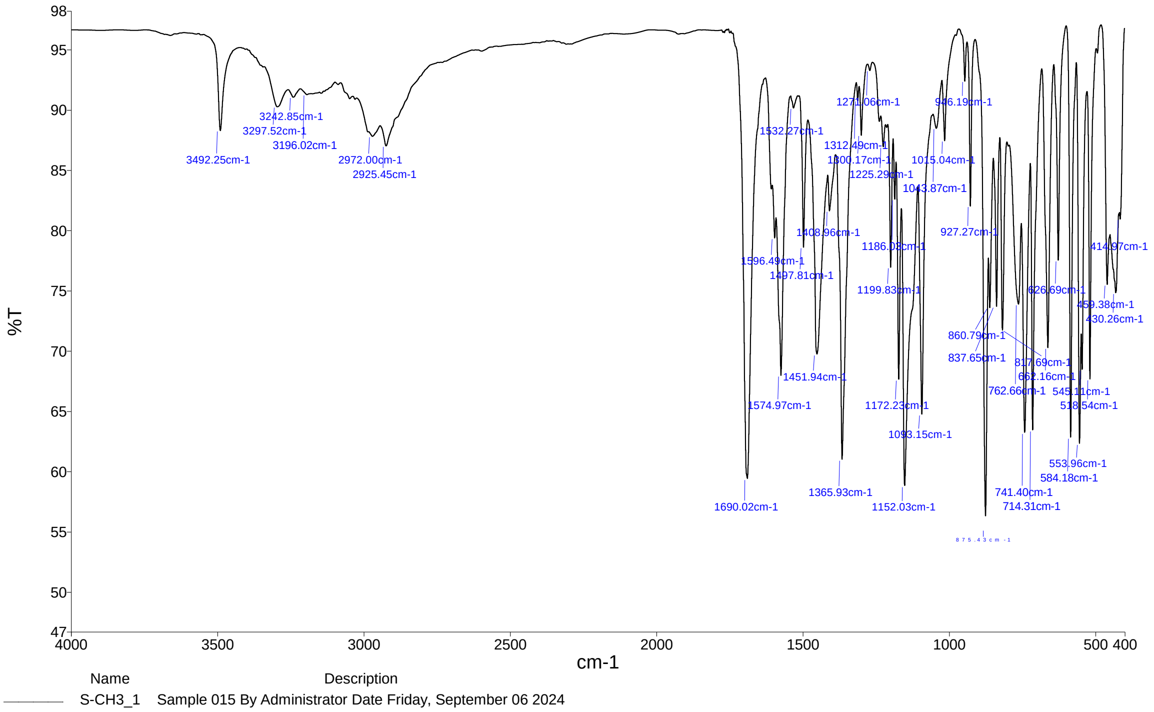

Figure S24. FT-IR spectrum of 6

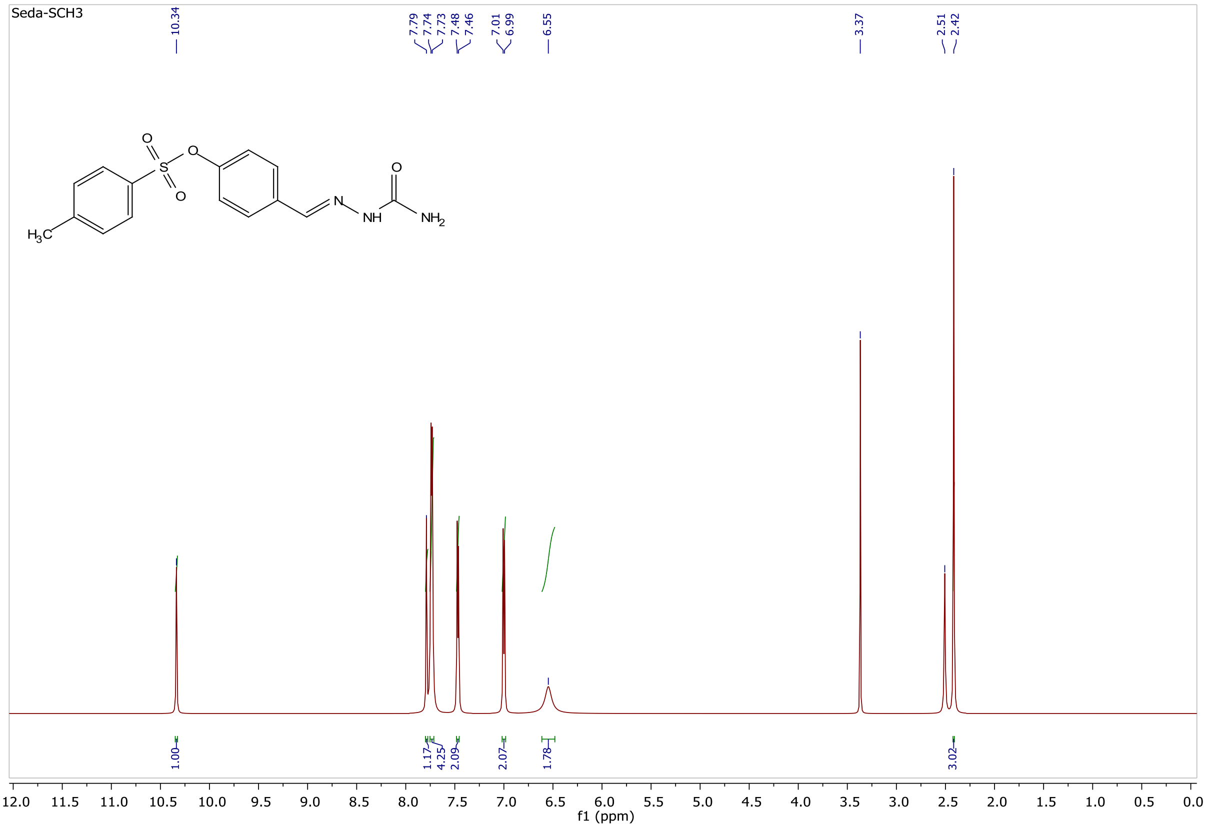

Figure S25. ^{1}H NMR spectrum of 6

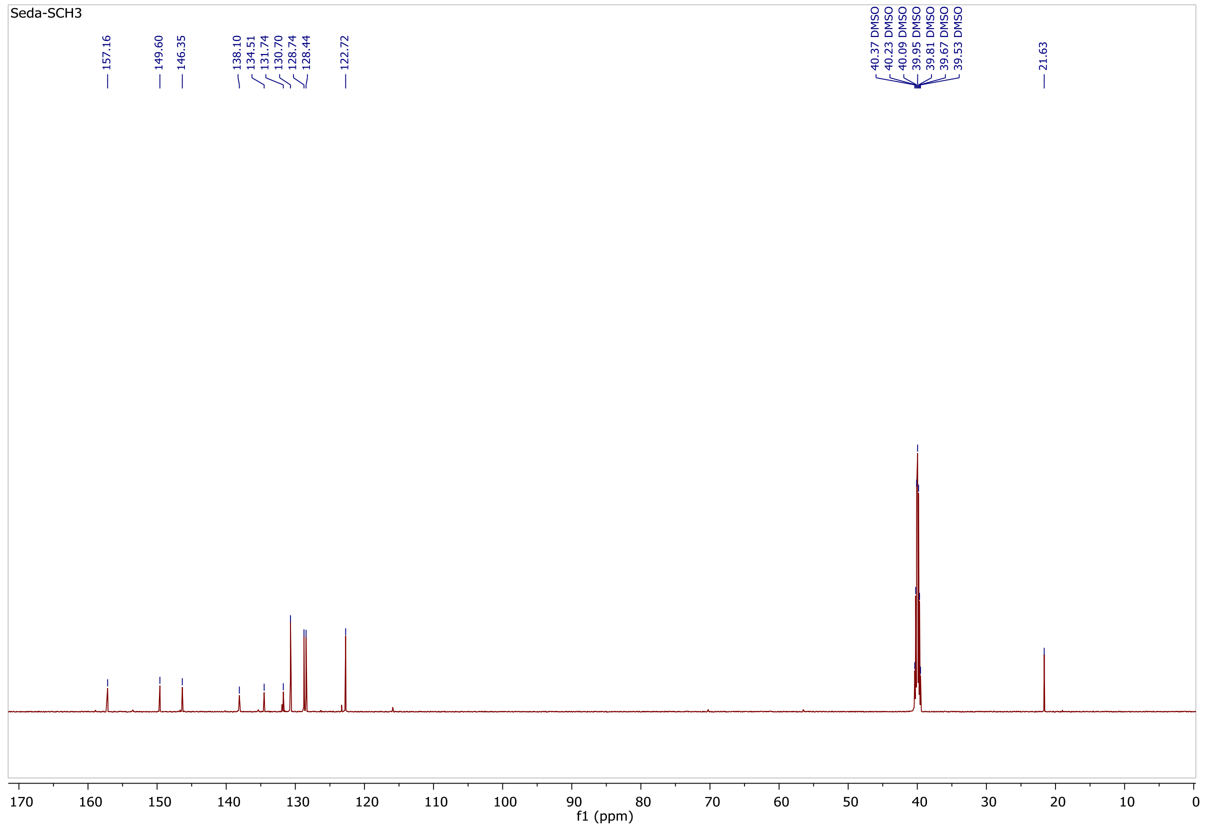

Figure S26. ^{13}C NMR spectrum of 6

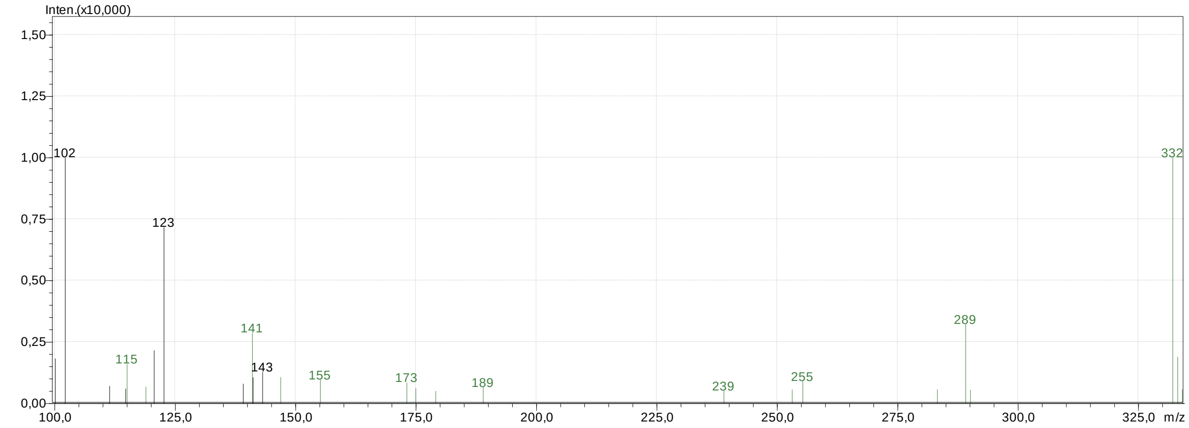

Figure S27. Mass spectrum of 6

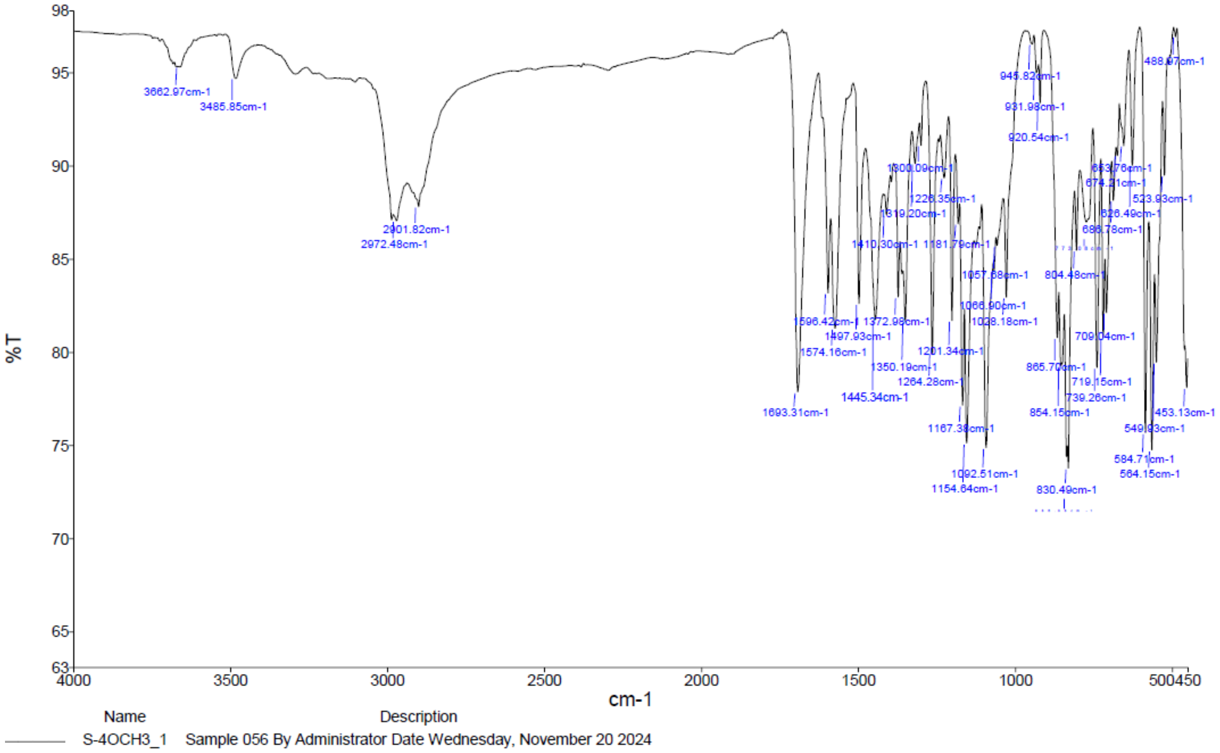

Figure S28. FT-IR spectrum of 7

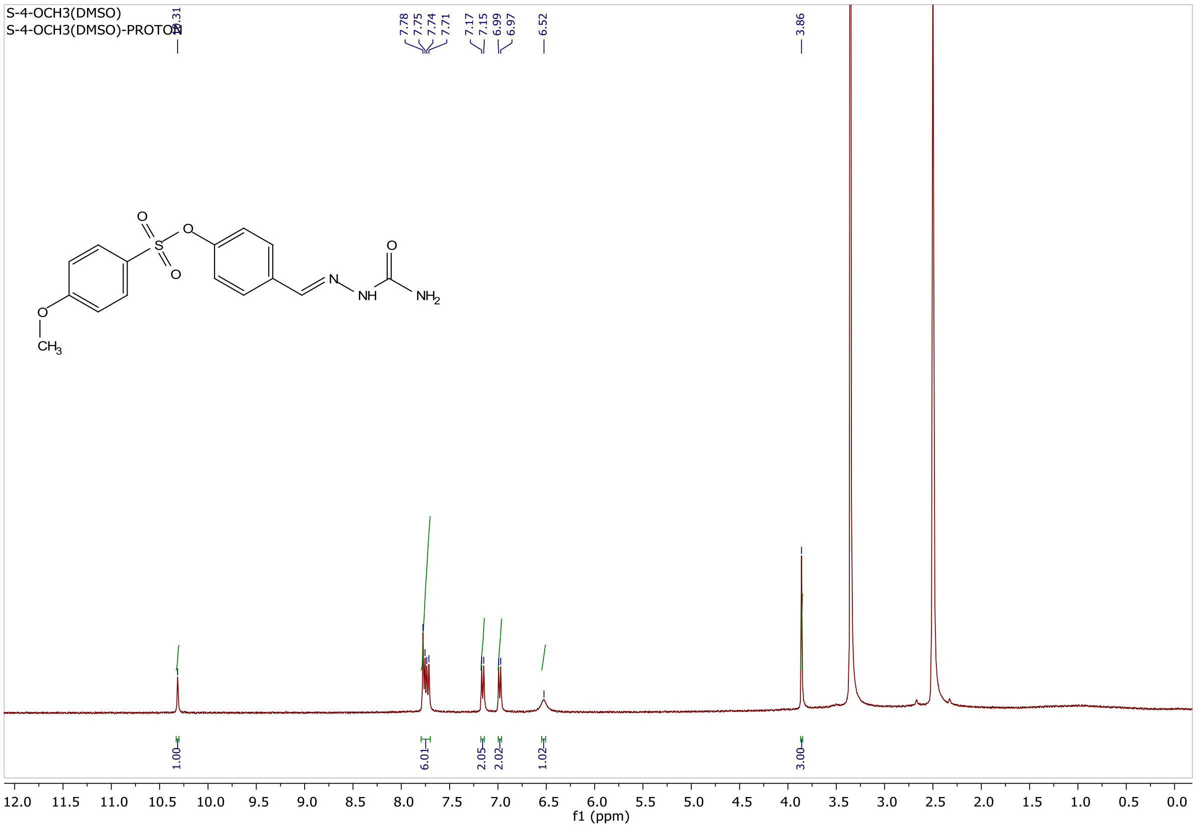

Figure S29. ^{1}H **NMR spectrum of 7**

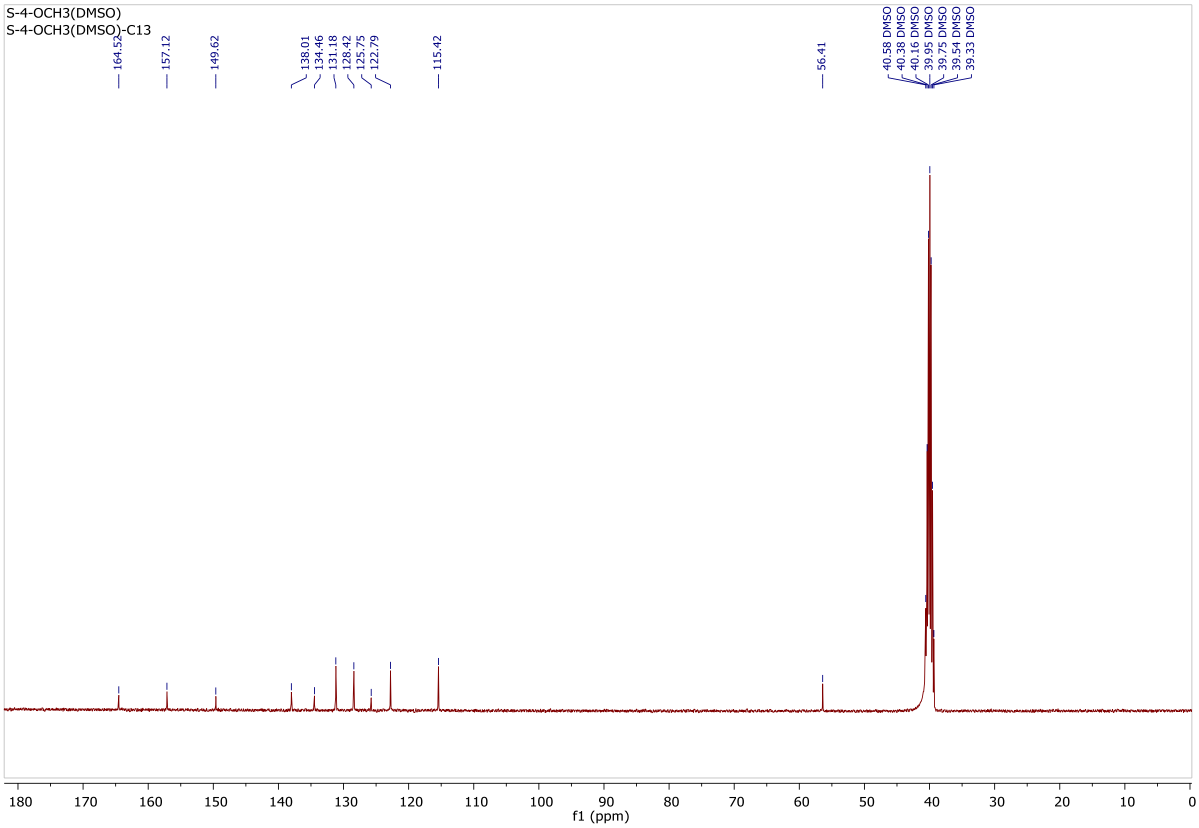

Figure S30. ^{13}C **NMR spectrum of 7**

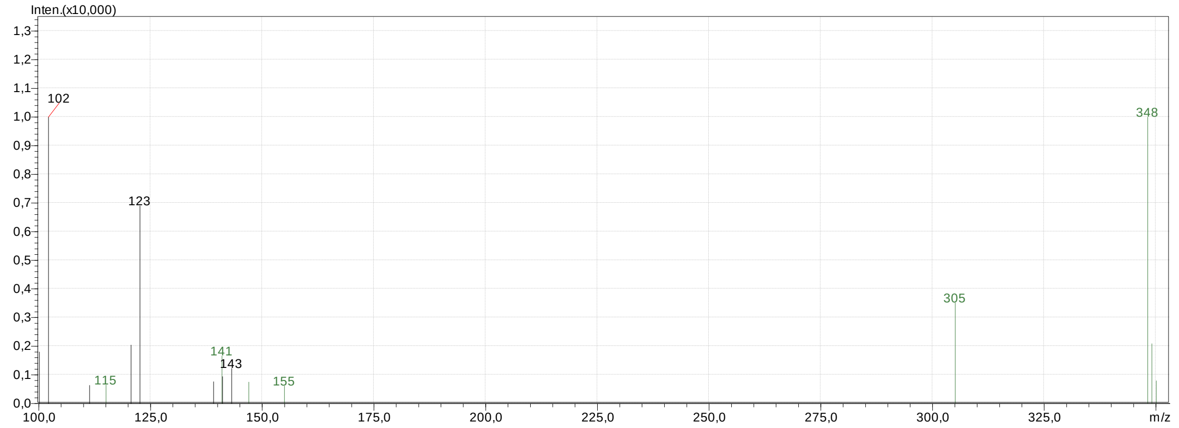

Figure S31. Mass spectrum of 7

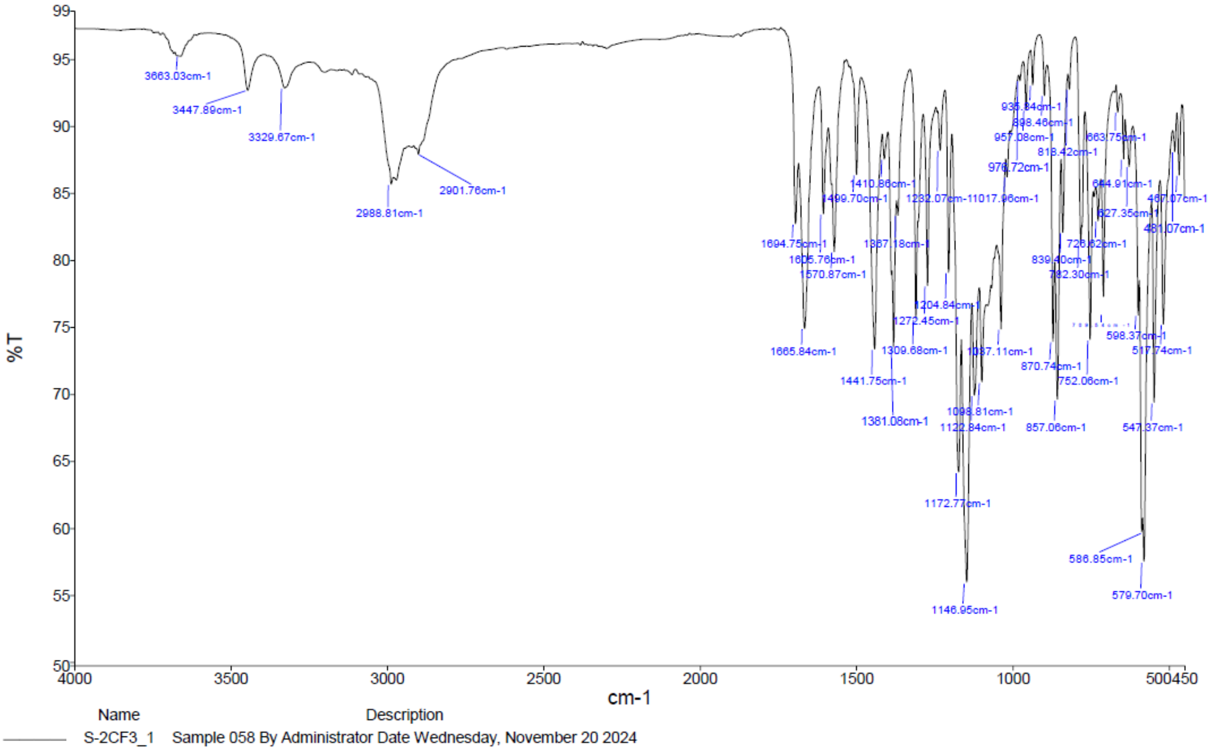

Figure S32. FT-IR spectrum of 8

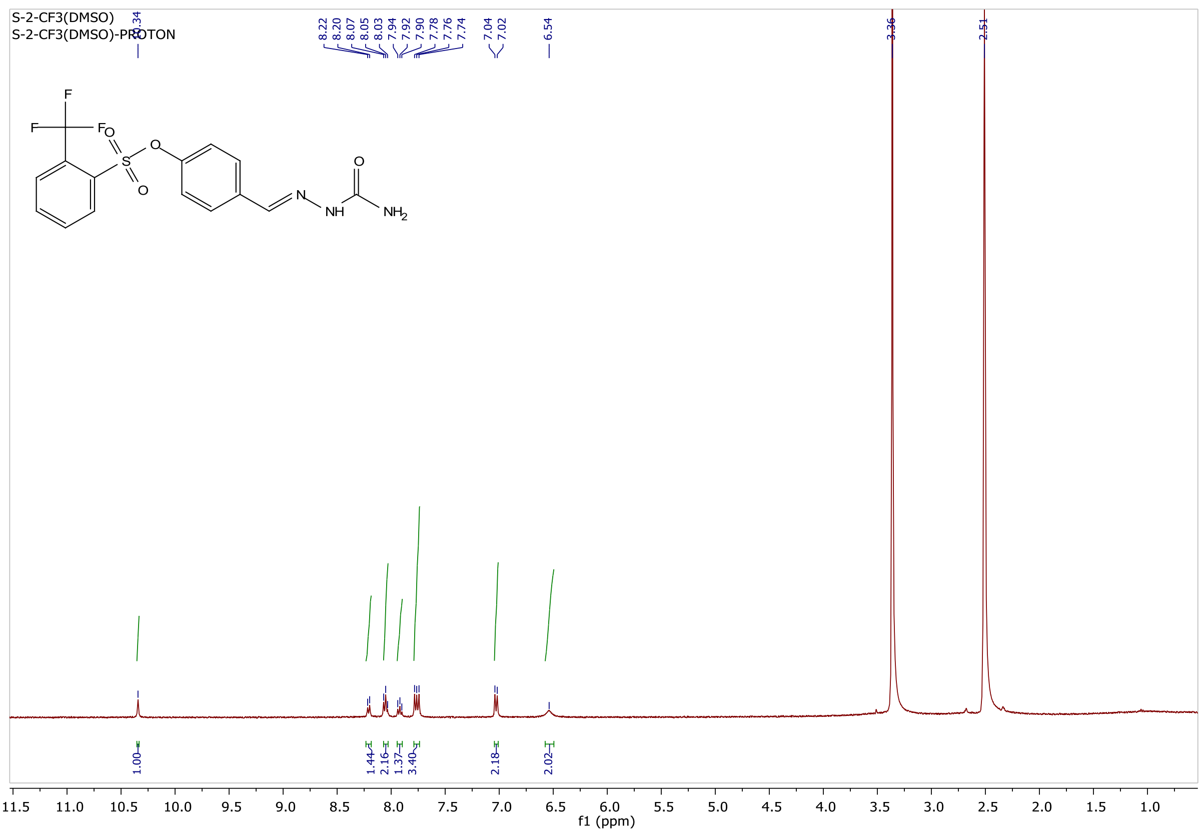

Figure S33. ^{1}H NMR spectrum of 8

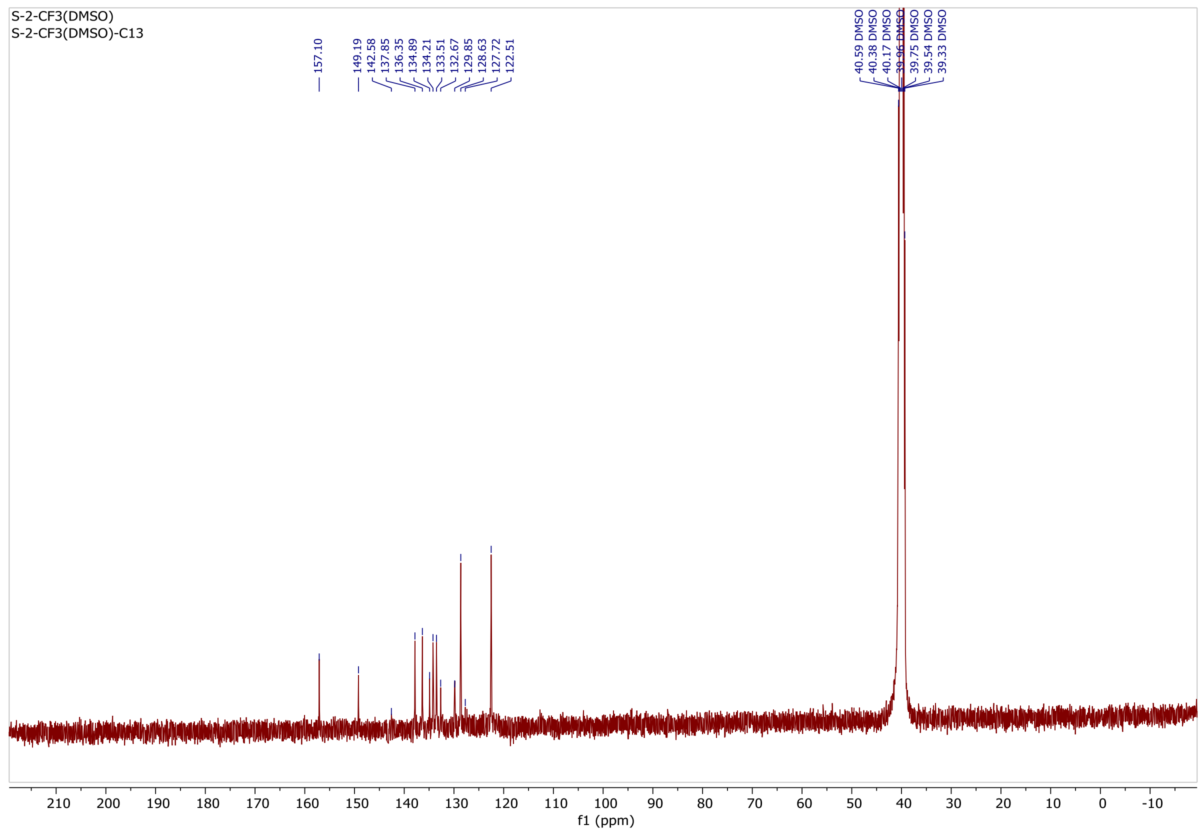

Figure S34. ^{13}C NMR spectrum of 8

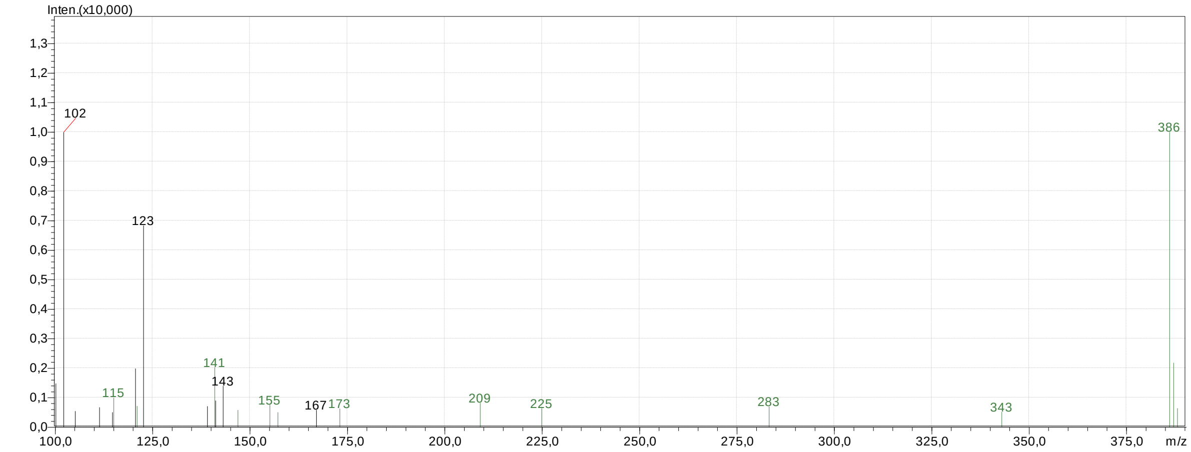

Figure S35. Mass spectrum of 8

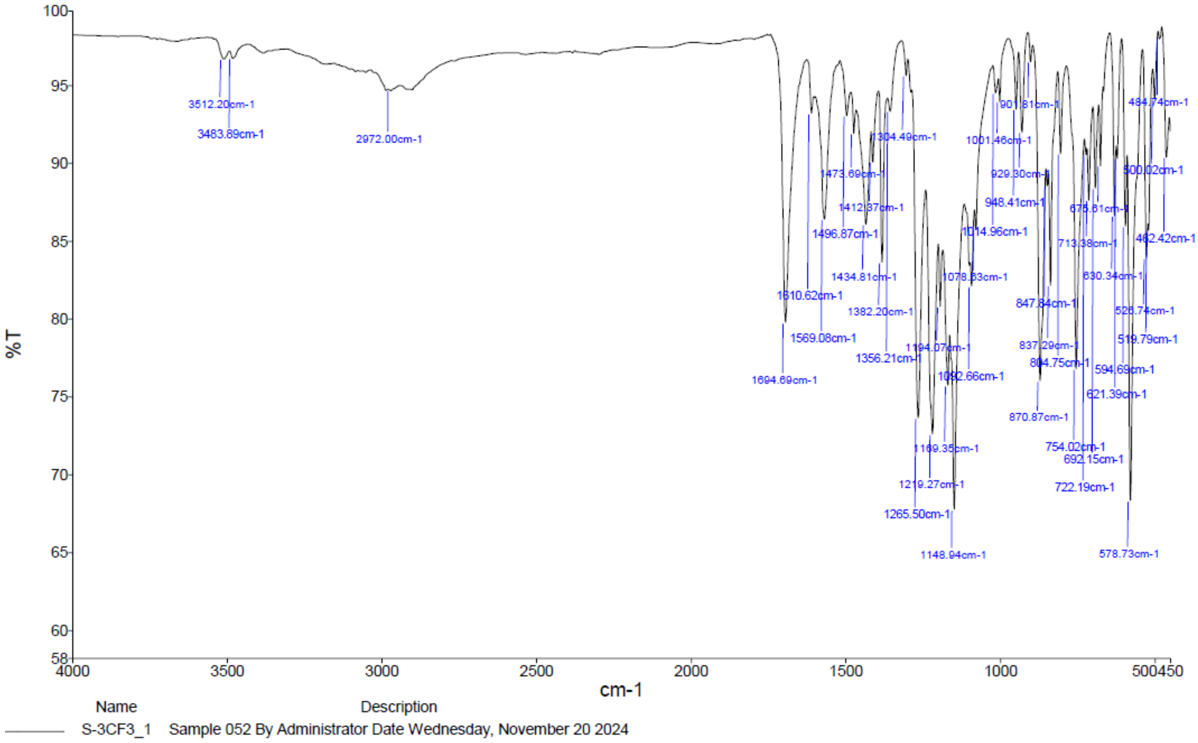

Figure S36. FT-IR spectrum of 9

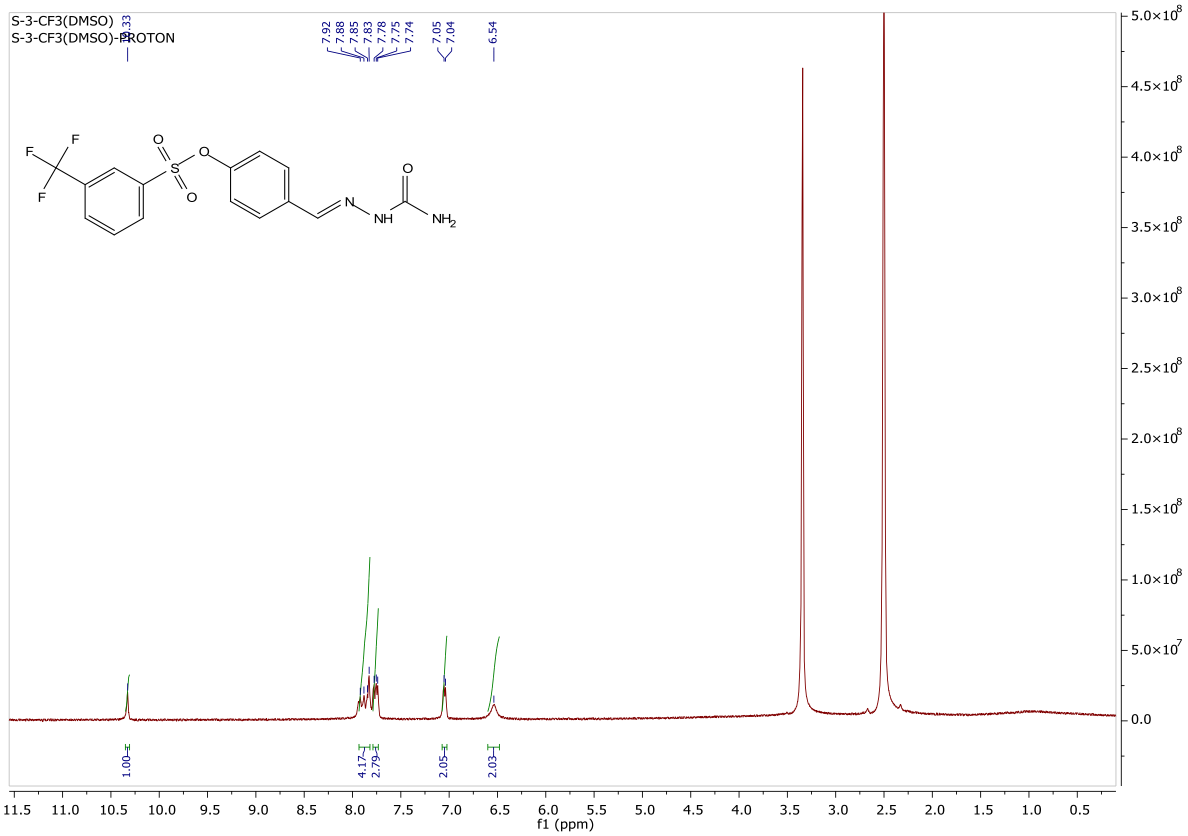

Figure S37. ^{1}H **NMR spectrum of 9**

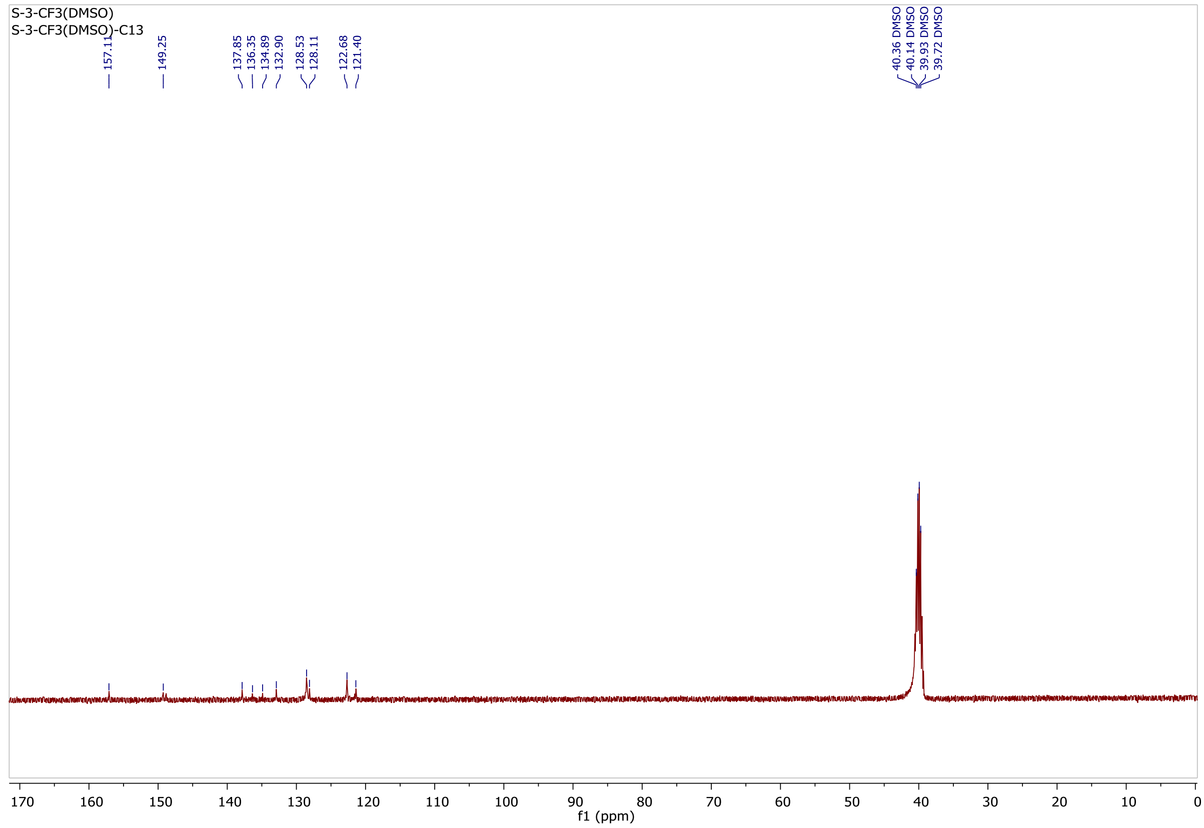

Figure S38. ^{13}C **NMR spectrum of 9**

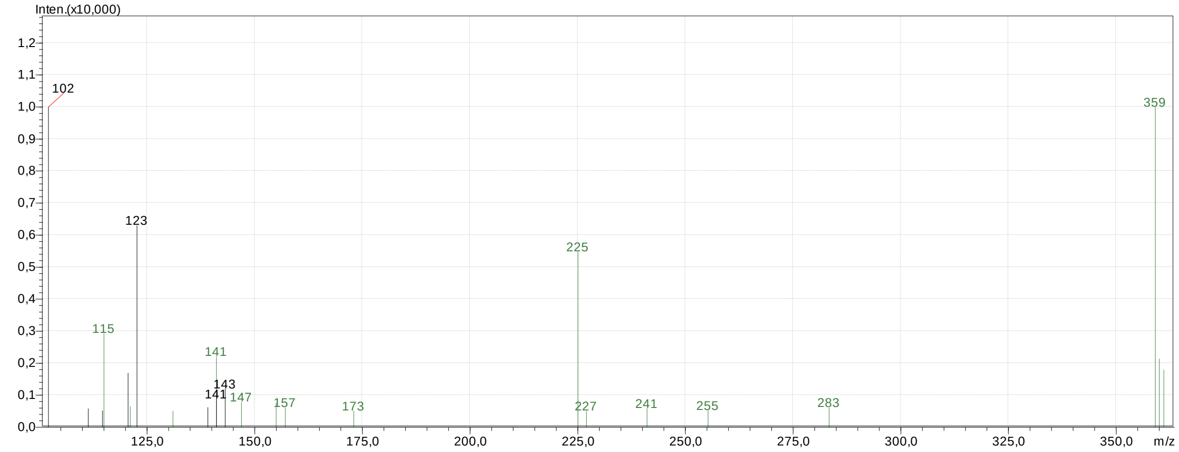

Figure S39. Mass spectrum of 9

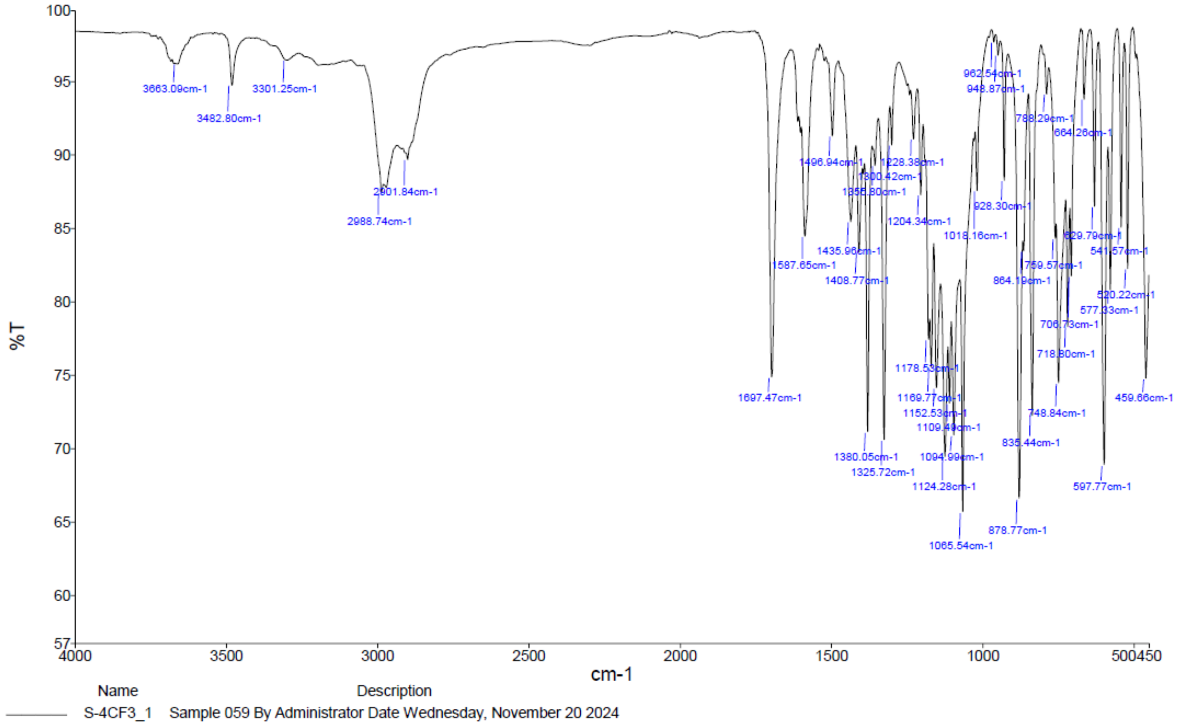

Figure S40. FT-IR spectrum of 10

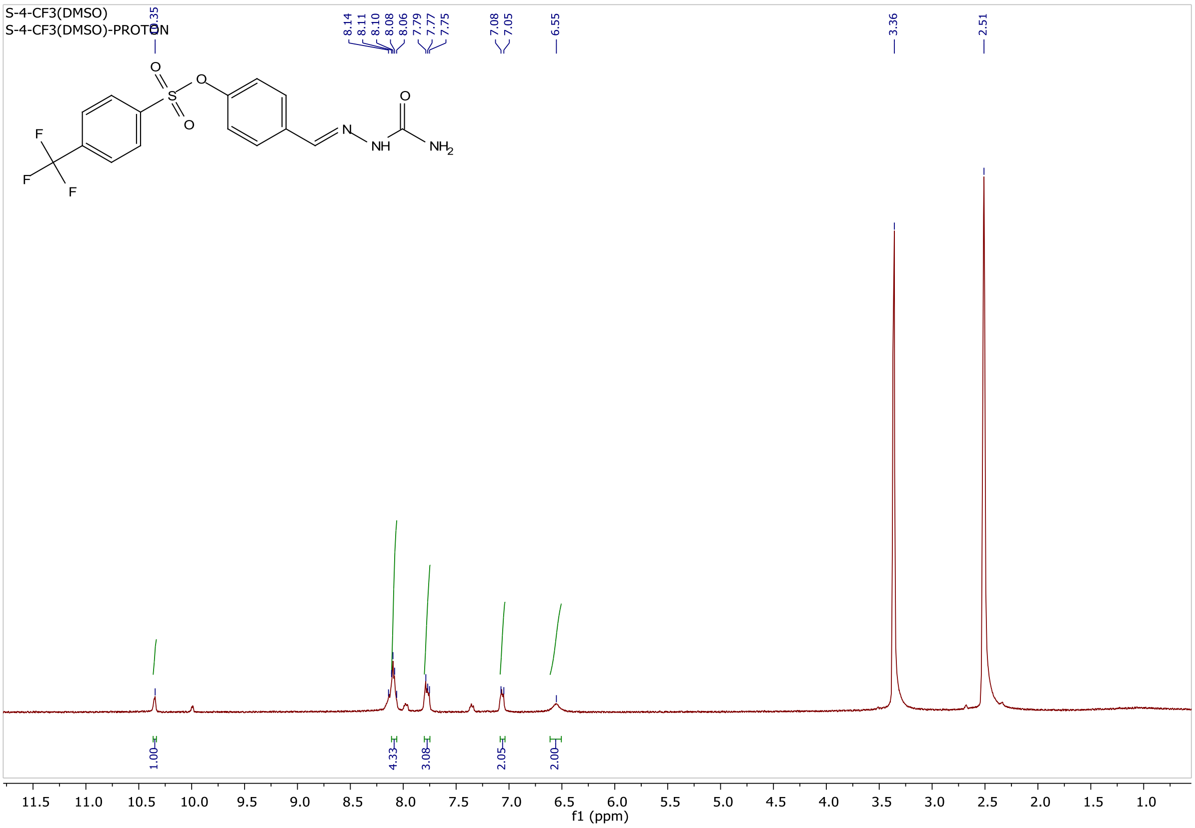

Figure S41. ^{1}**H NMR spectrum of 10**

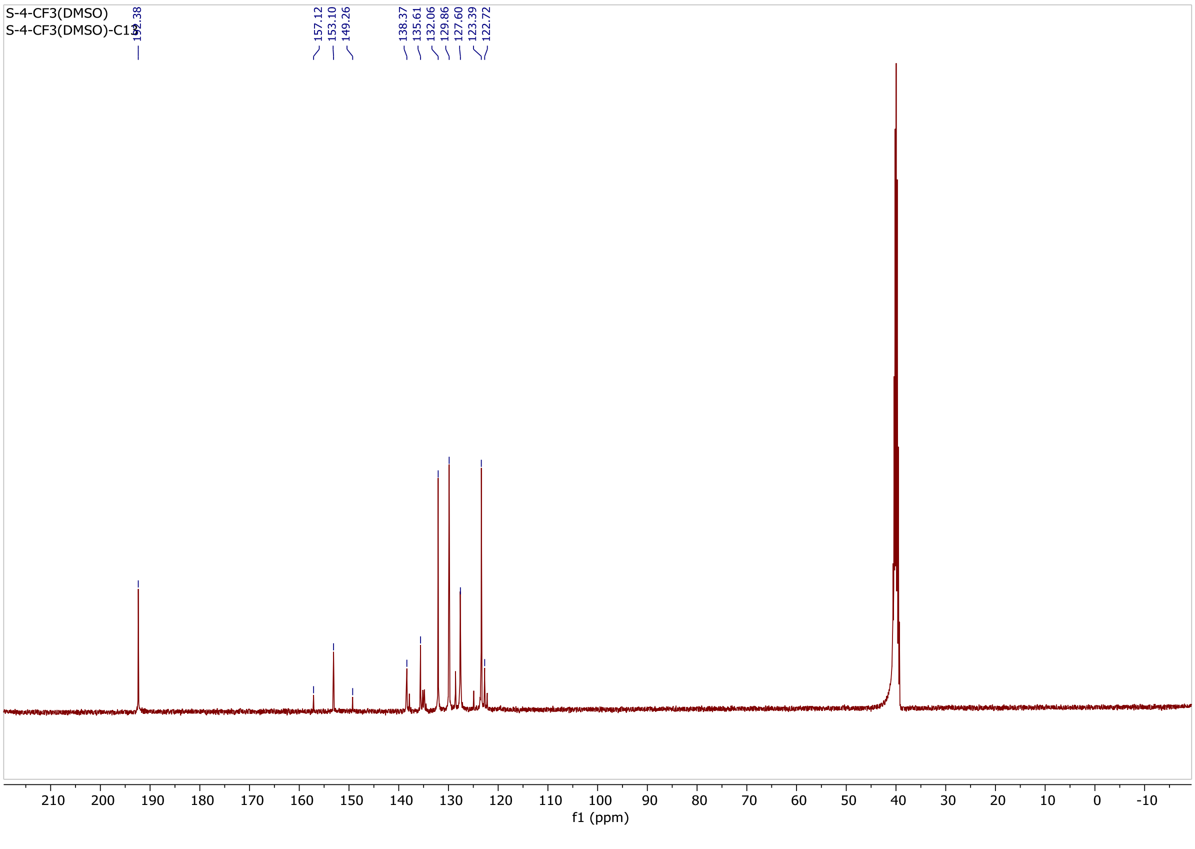

Figure S42. ^{13}**C NMR spectrum of 10**

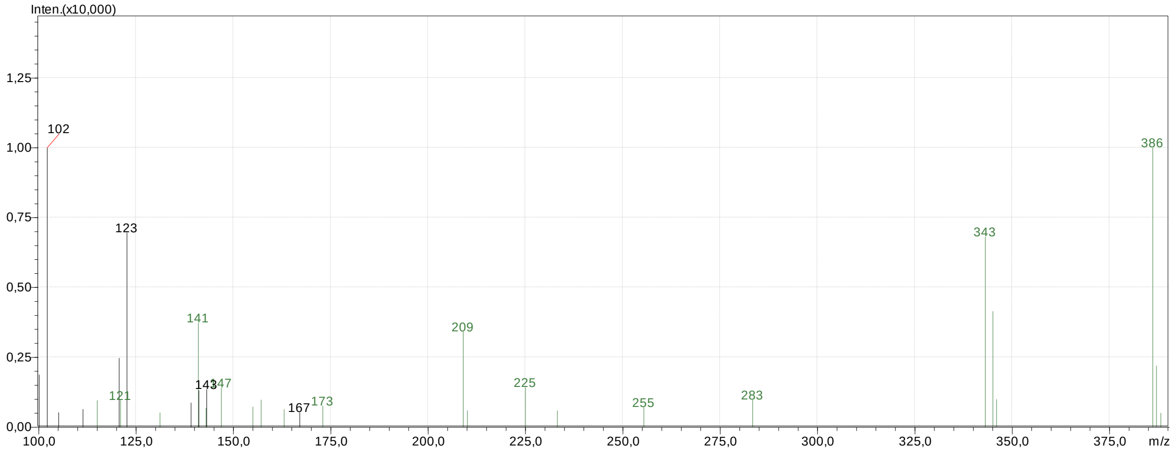

Figure S43. Mass spectrum of 10

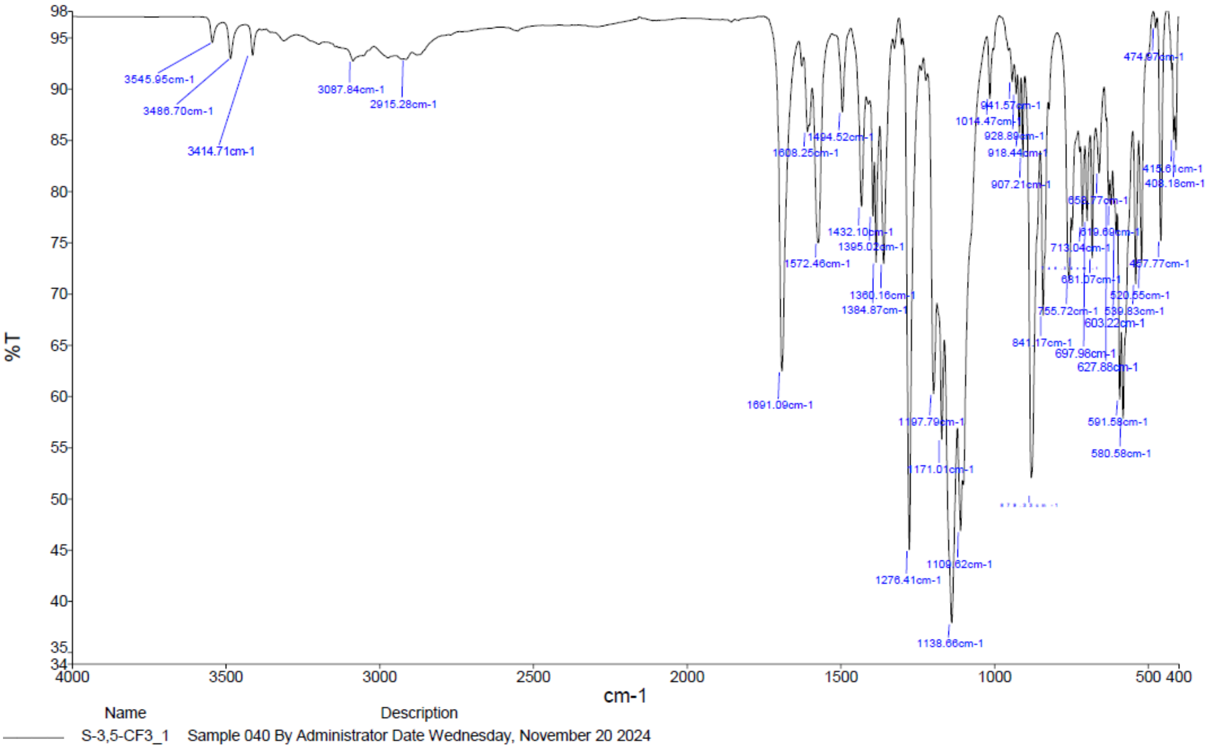

Figure S44. FT-IR spectrum of 11

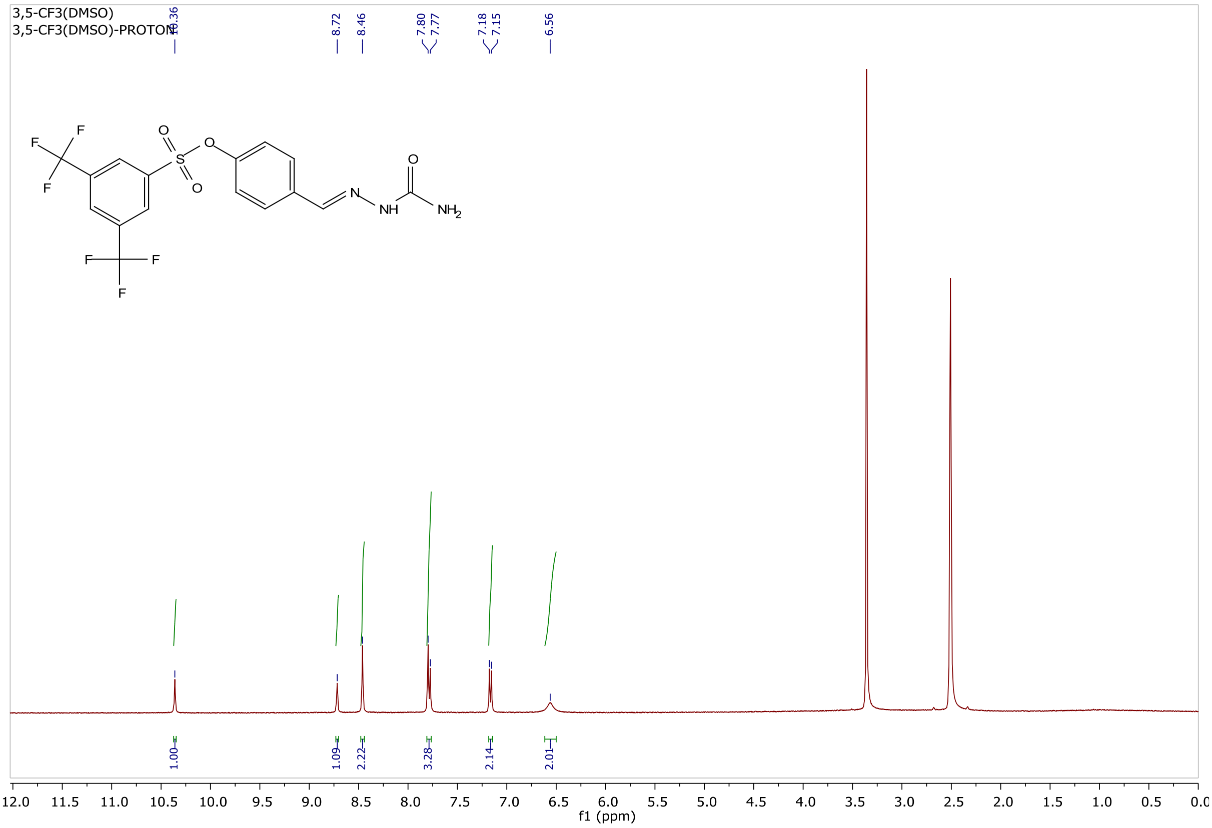

Figure S45. ^{1}**H NMR spectrum of 11**

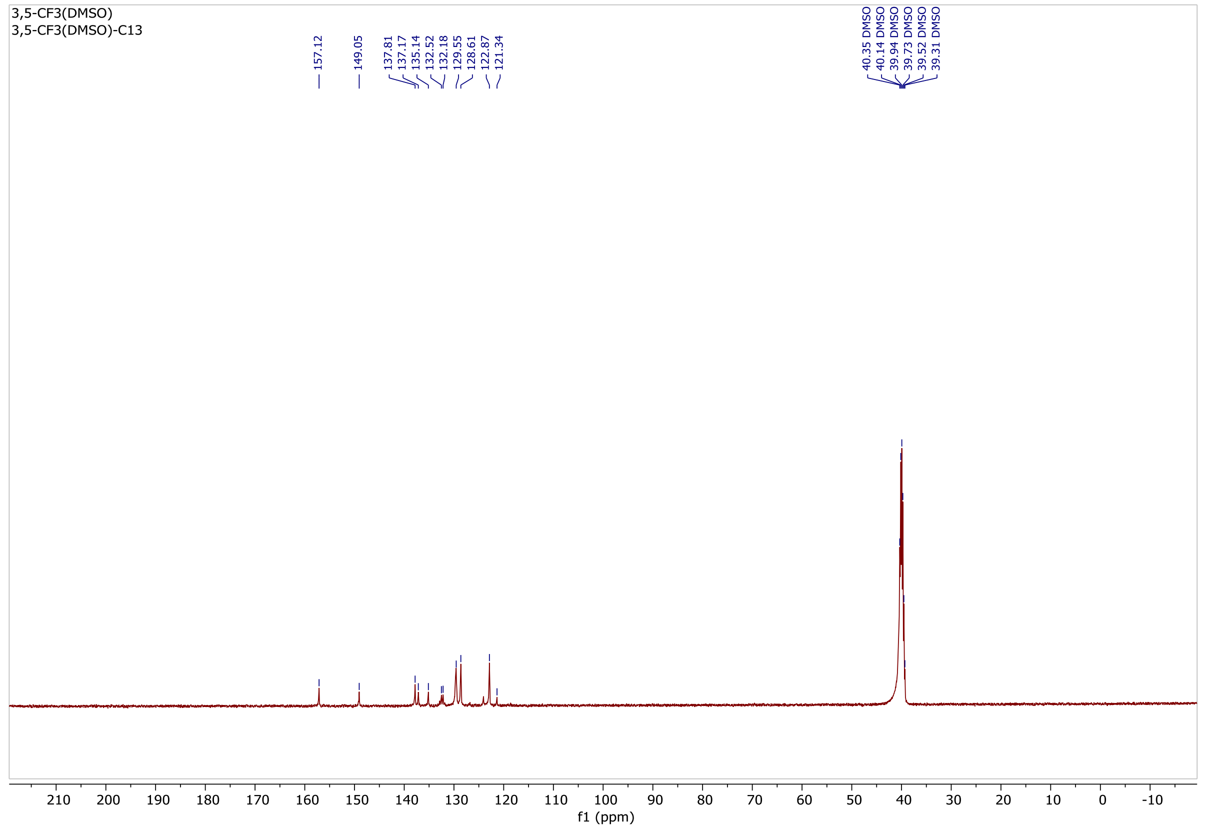

Figure S46. ^{13}**C NMR spectrum of 11**

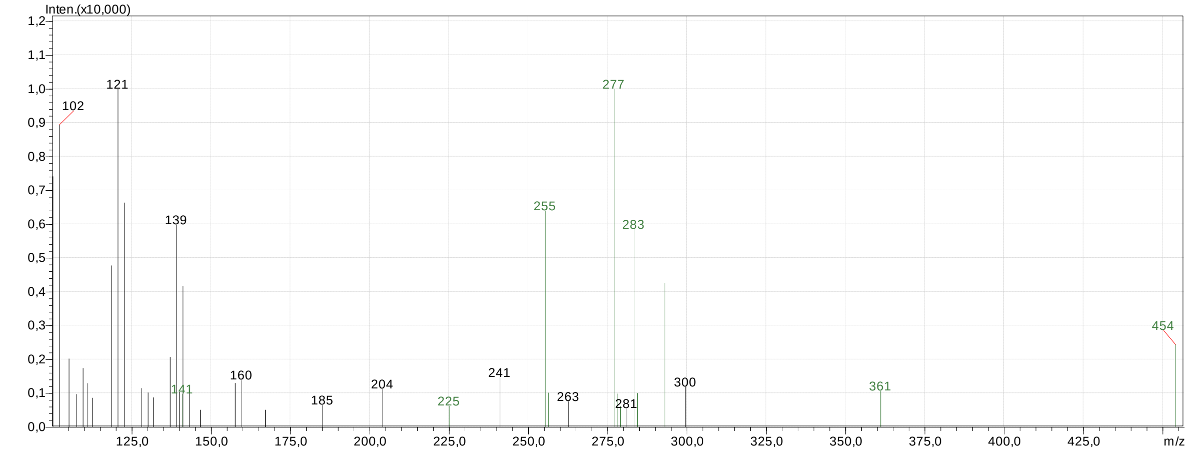

Figure S47. Mass spectrum of 11

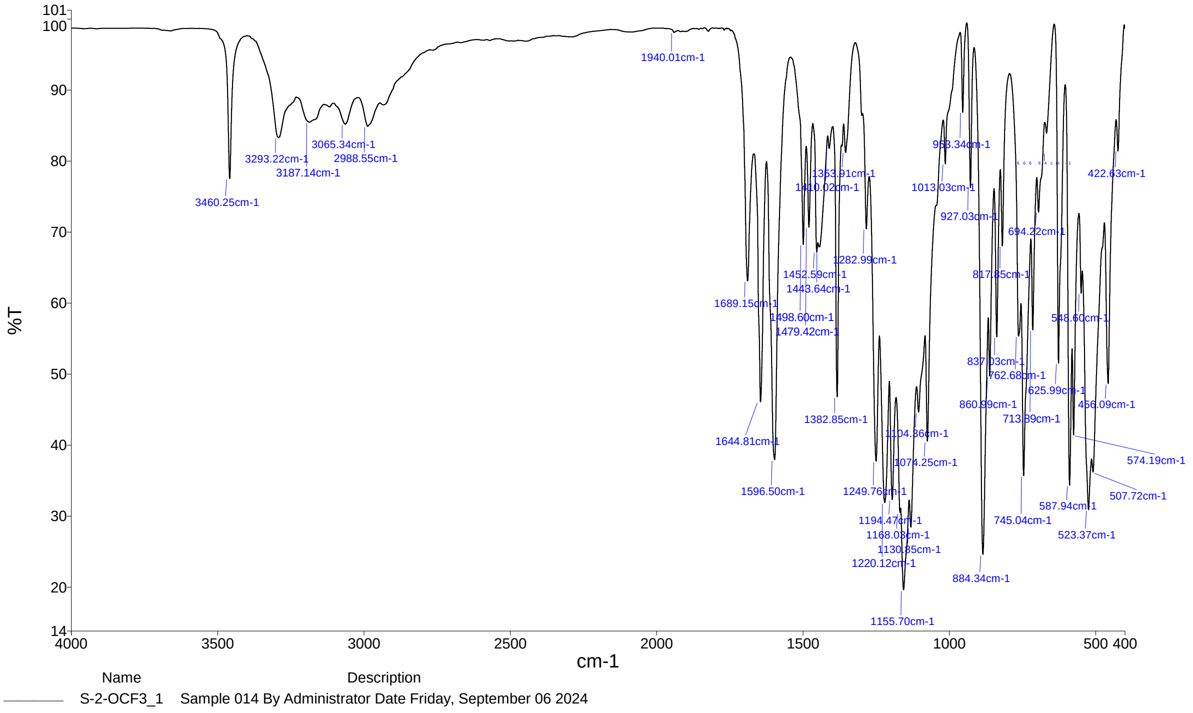

Figure S48. FT-IR spectrum of 12

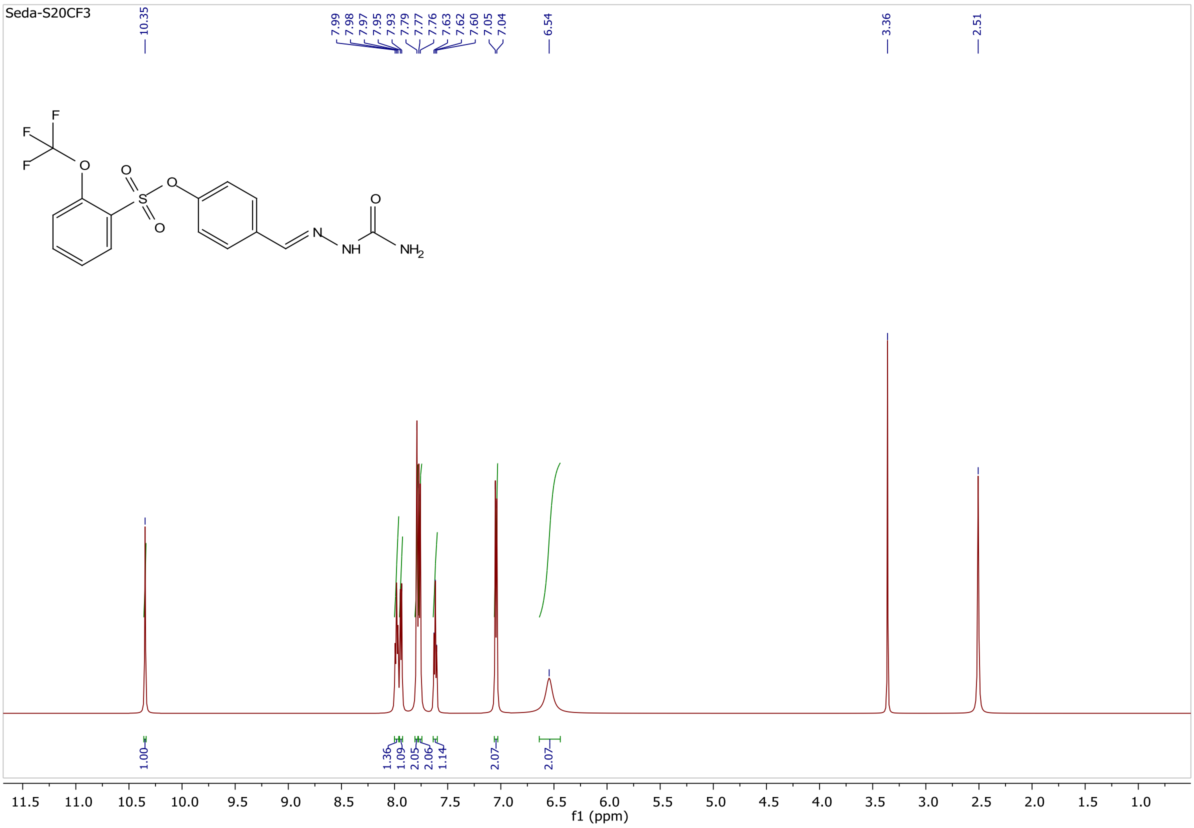

Figure S49. ^{1}**H NMR spectrum of 12**

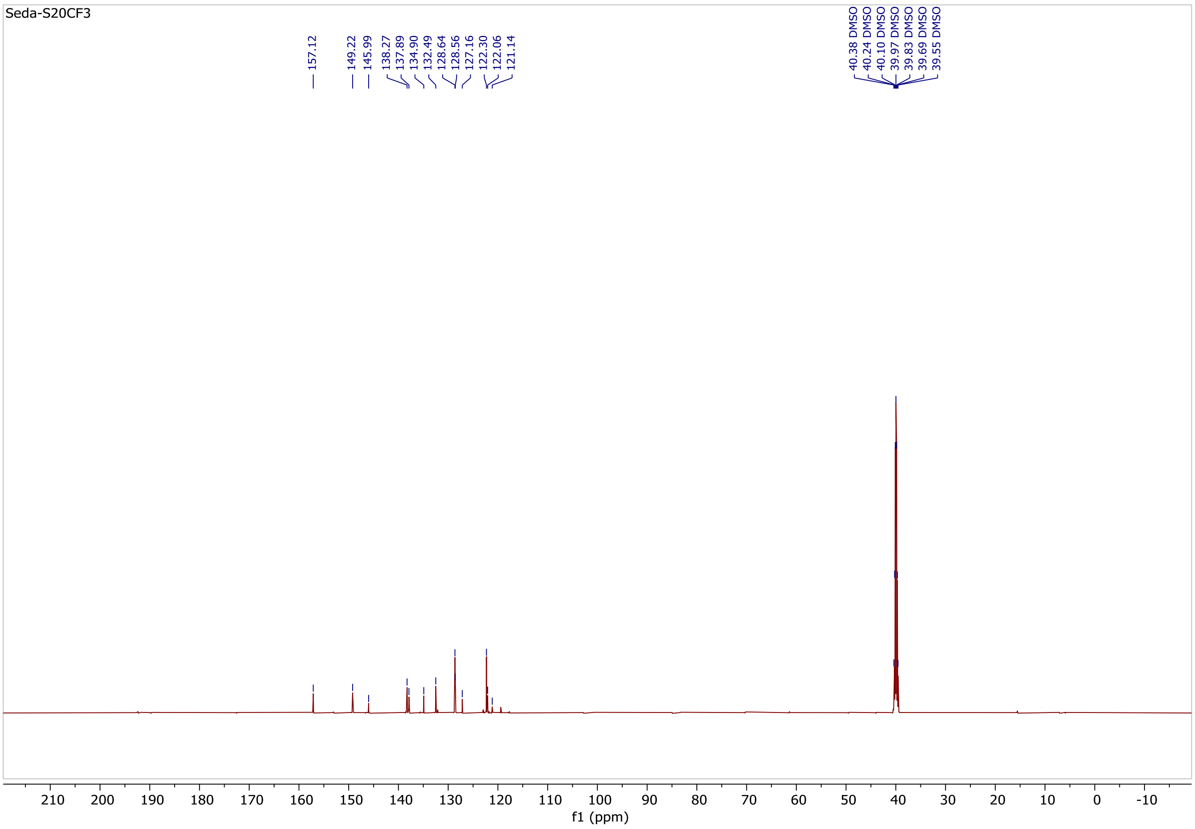

Figure S50. ^{13}**C NMR spectrum of 12**

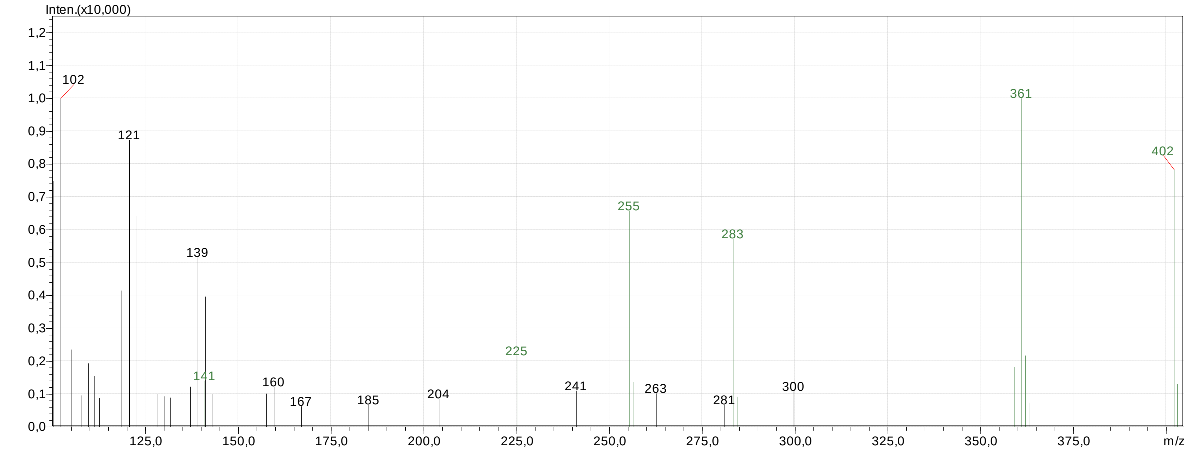

Figure S51. Mass spectrum of 12

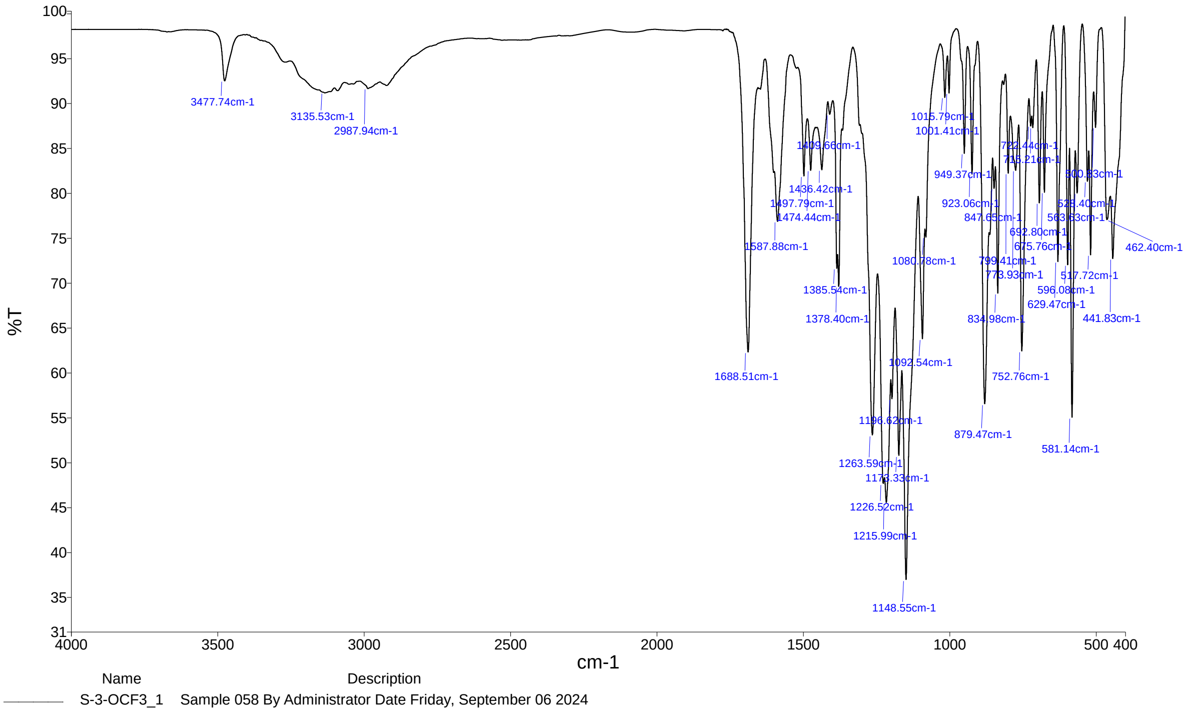

Figure S52. FT-IR spectrum of 13

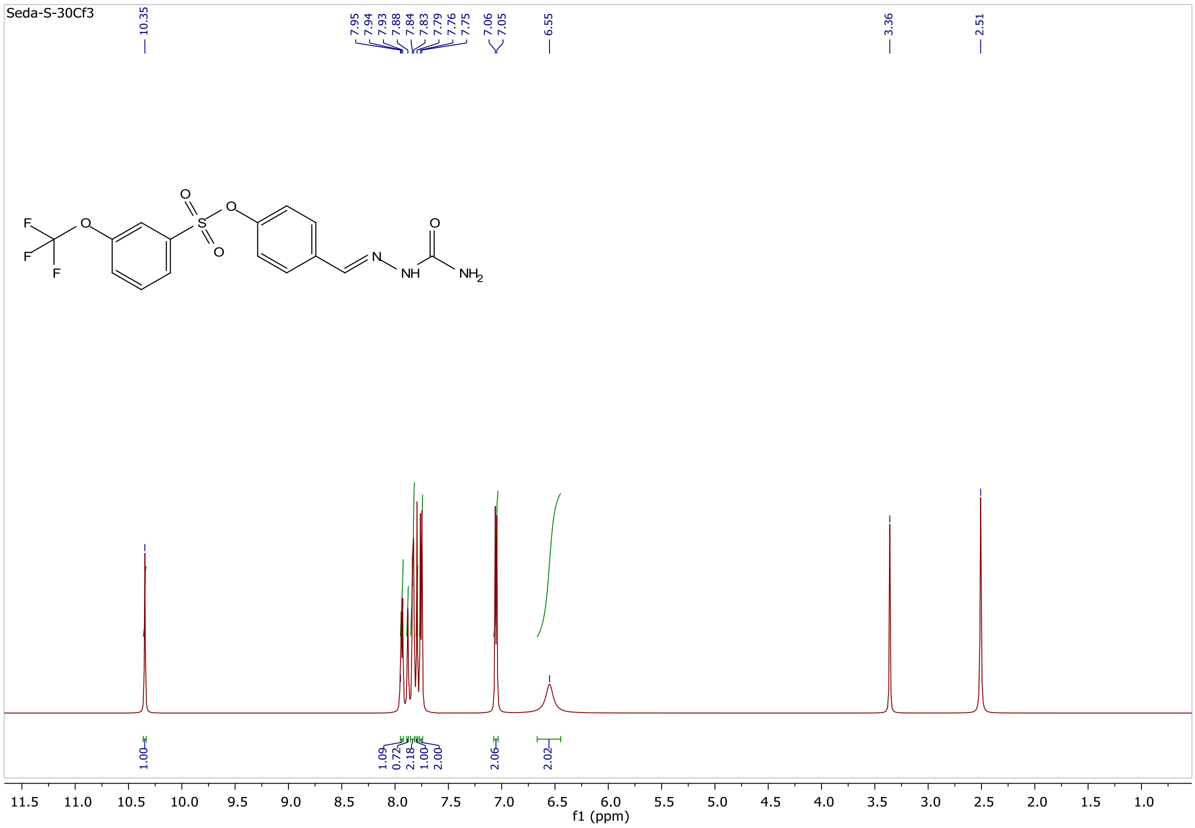

Figure S53. ^{1}H NMR spectrum of 13

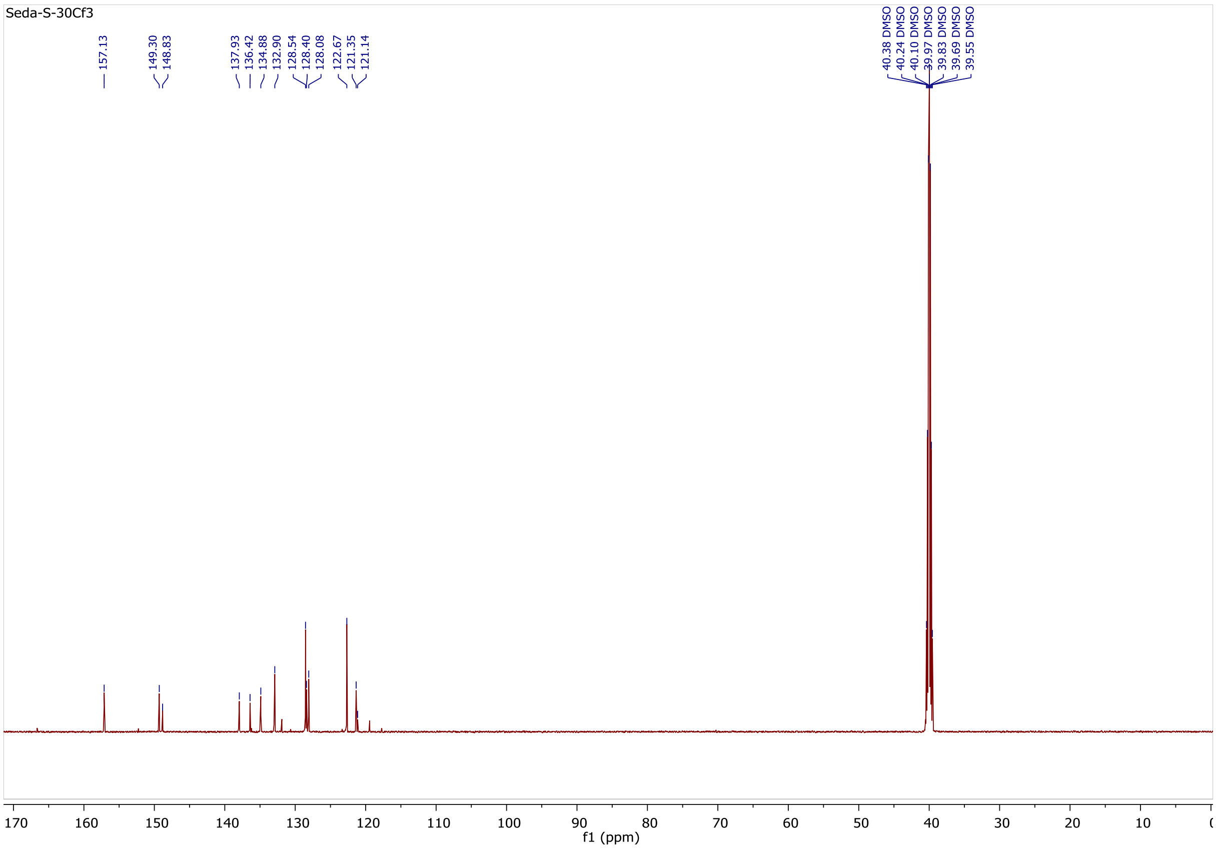

Figure S54. ^{13}C NMR spectrum of 13

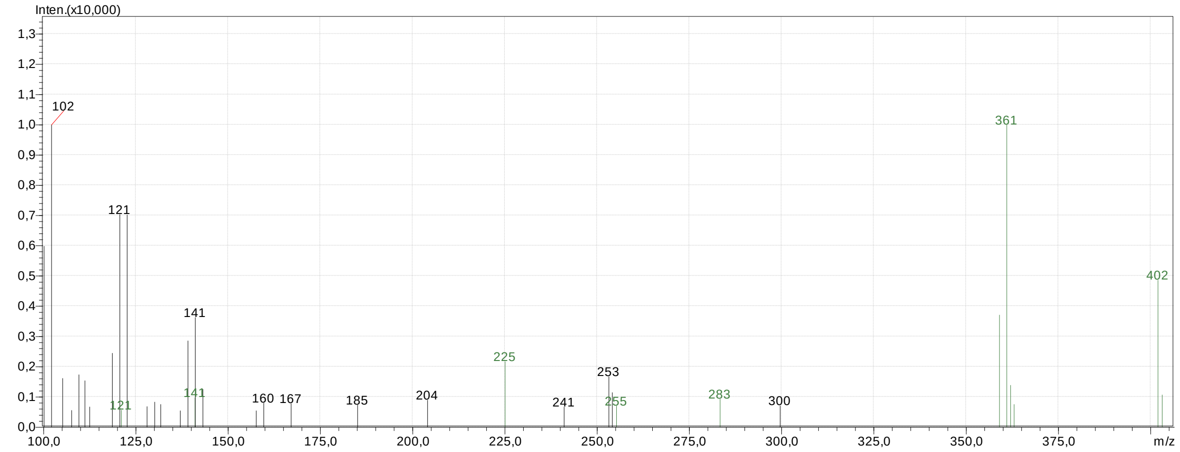

Figure S55. Mass spectrum of 13

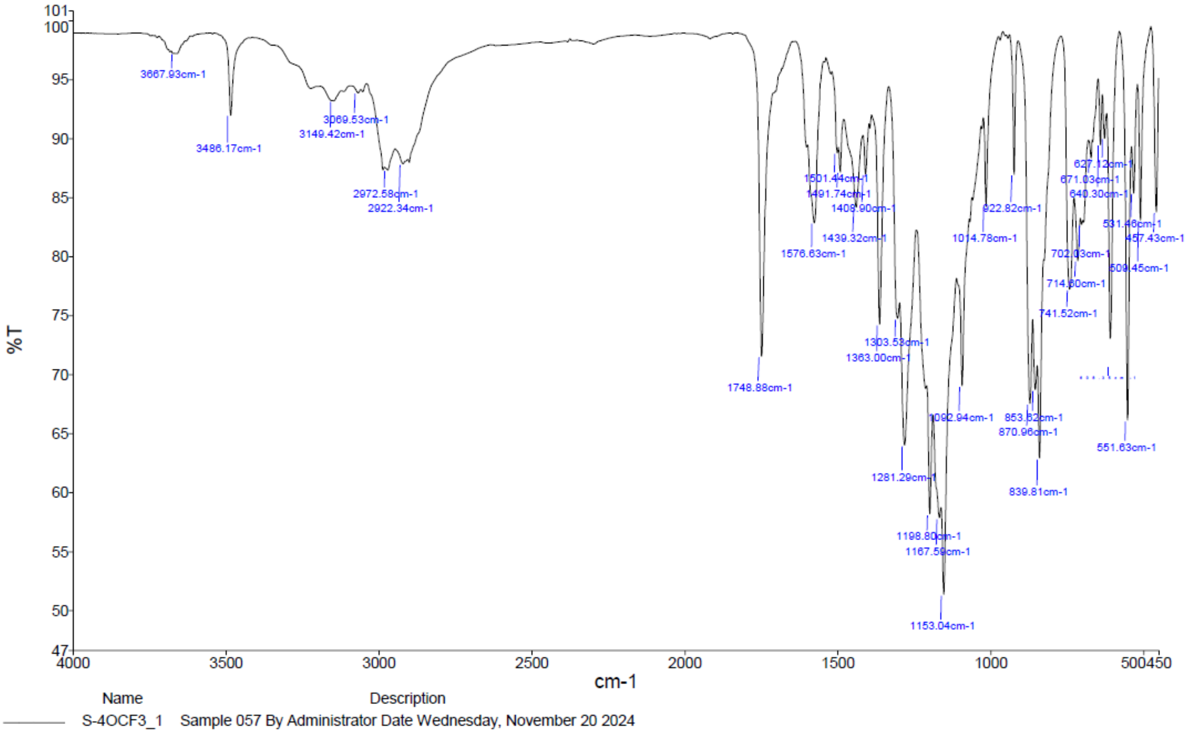

Figure S56. FT-IR spectrum of 14

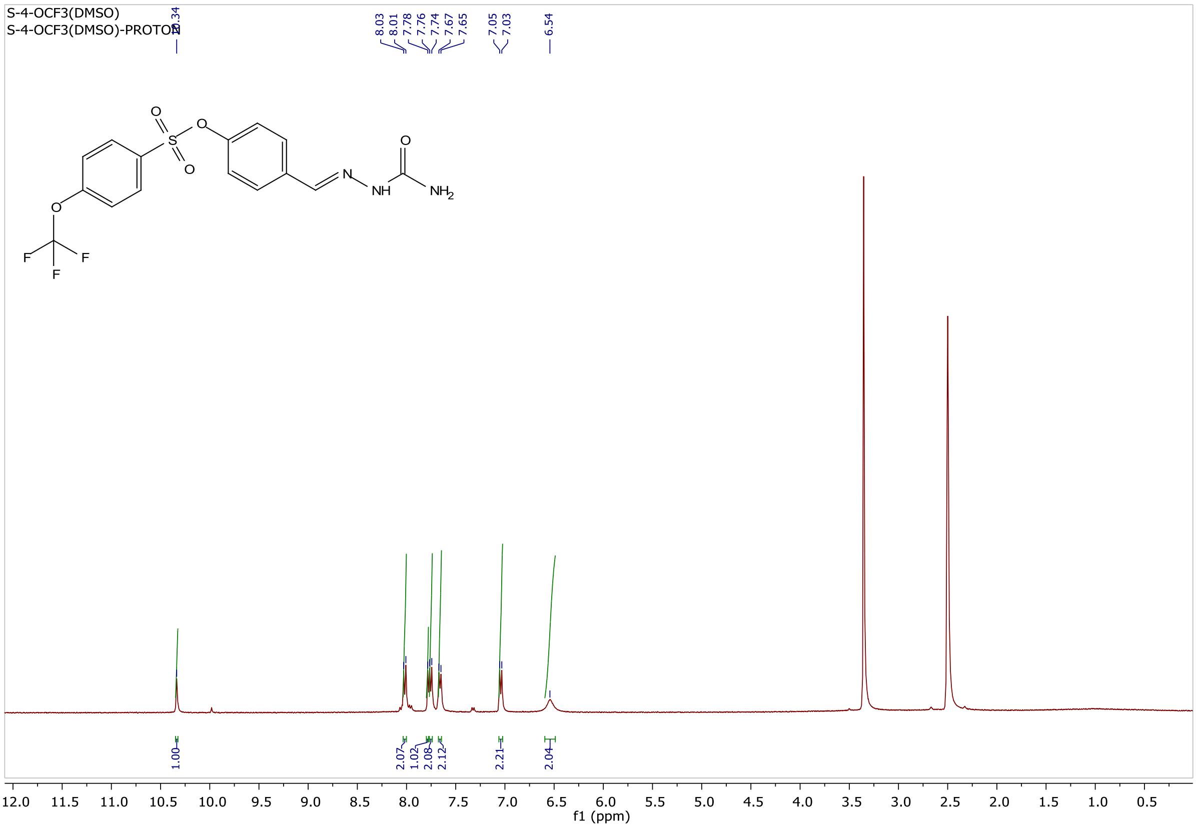

Figure S57. ^{1}H **NMR spectrum of 14**

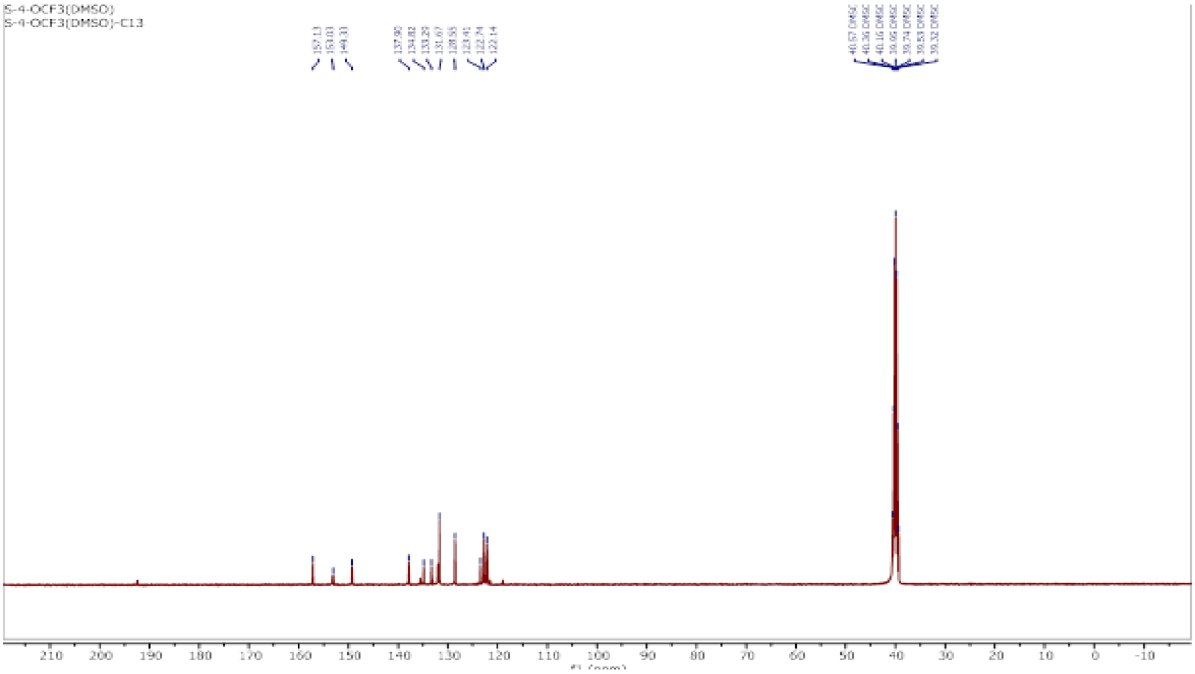

Figure S58. ^{13}C **NMR spectrum of 14**

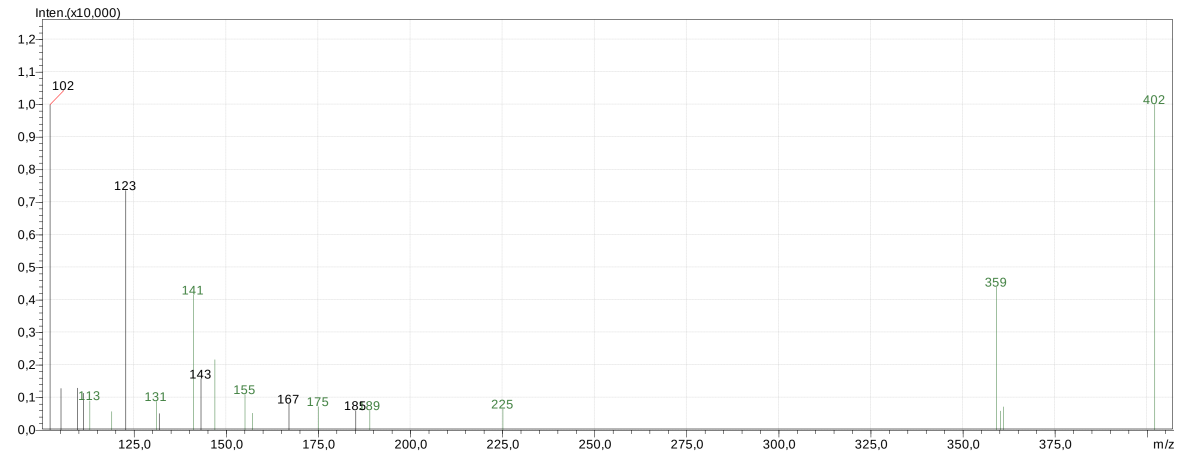

Figure S59. Mass spectrum of 14

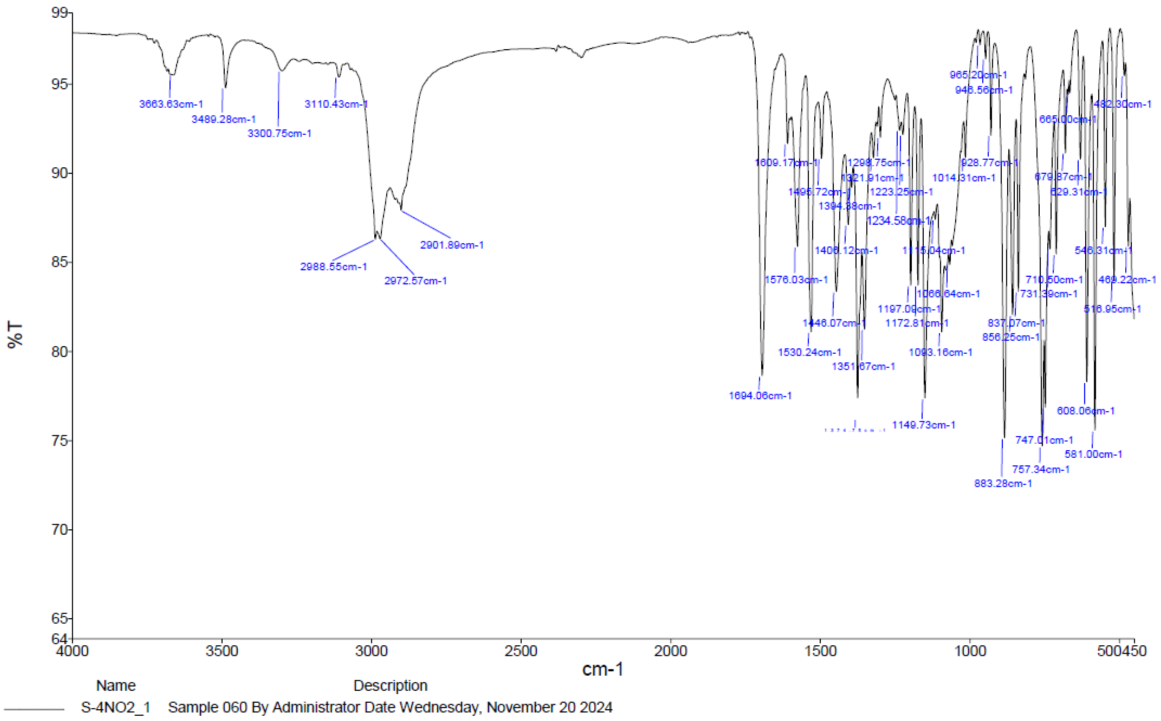

Figure S60. FT-IR spectrum of 15

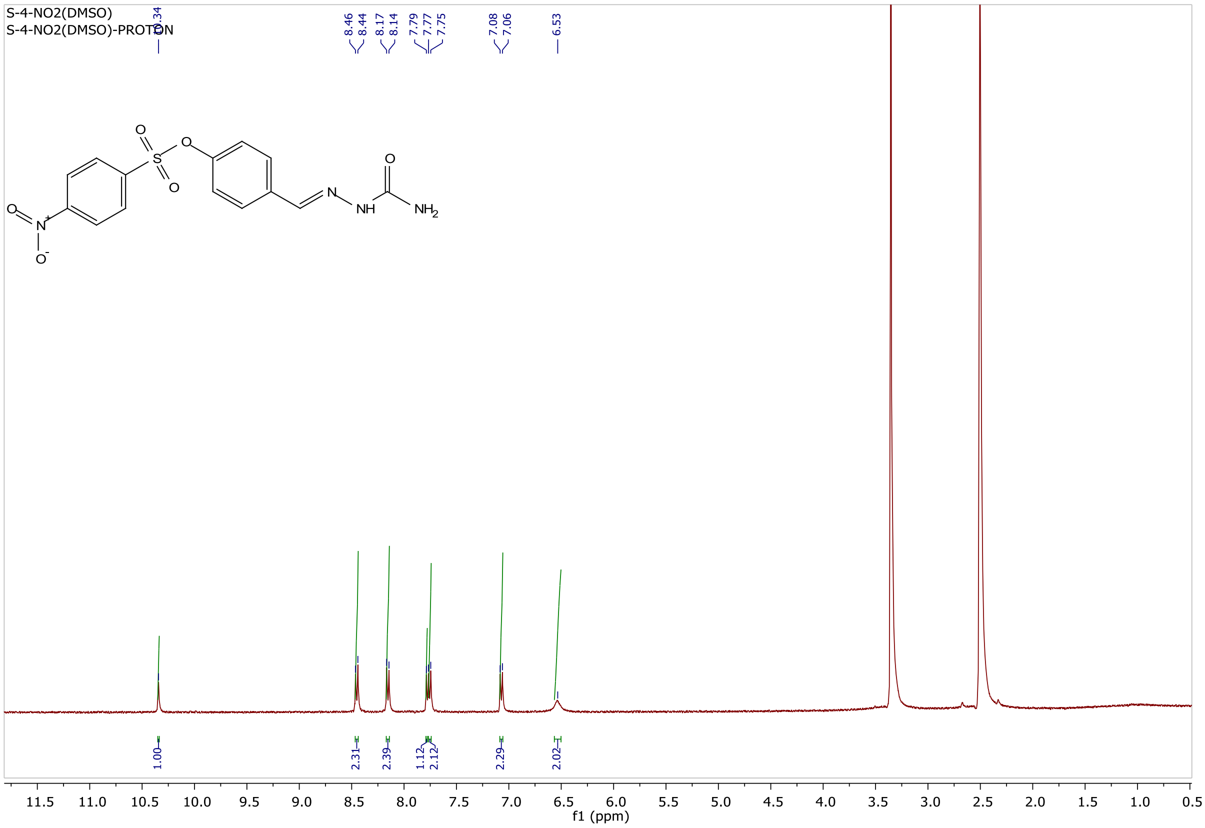

Figure S61. ^{1}**H NMR spectrum of 15**

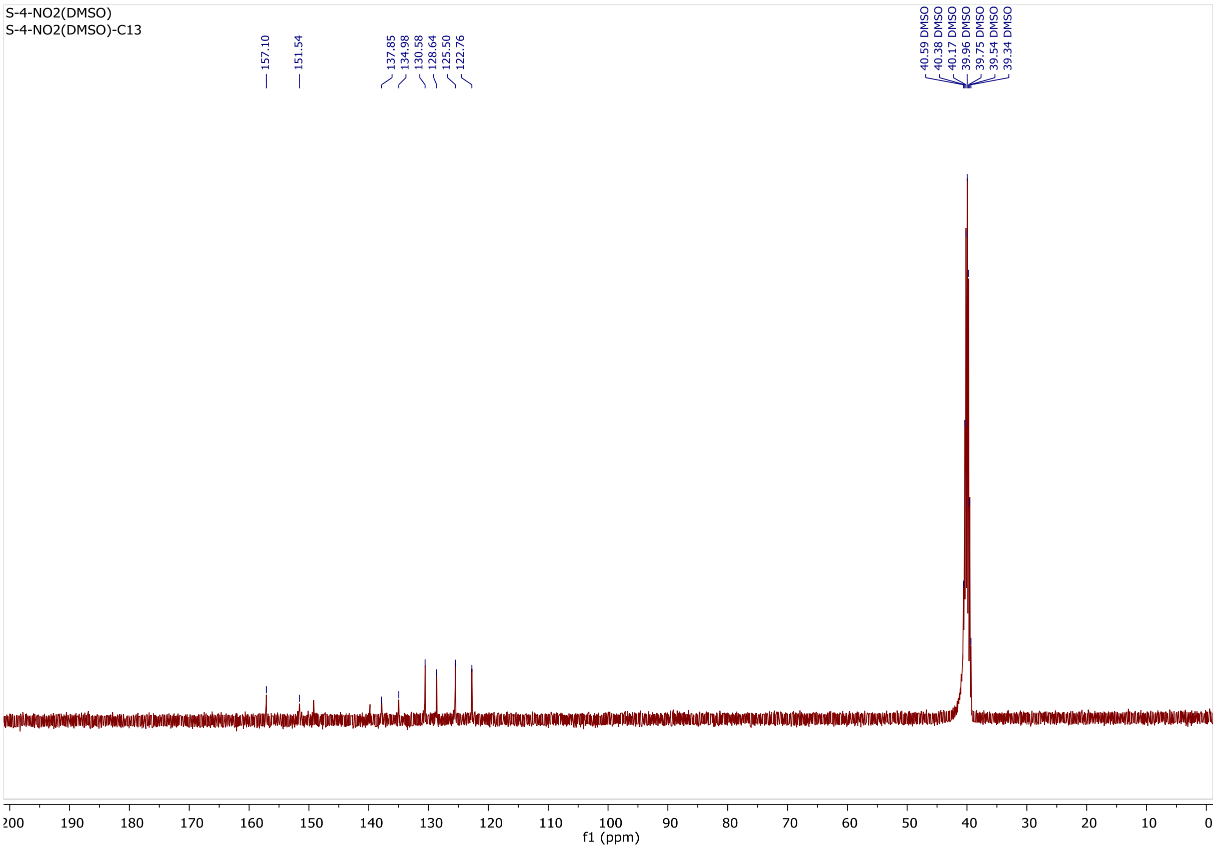

Figure S62. ^{13}**C NMR spectrum of 15**

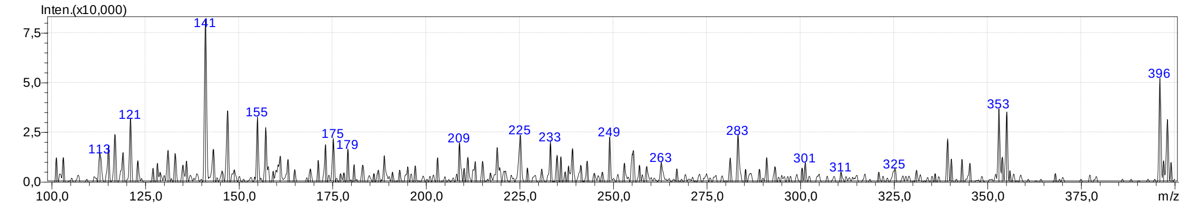

Figure S63. Mass spectrum of 15

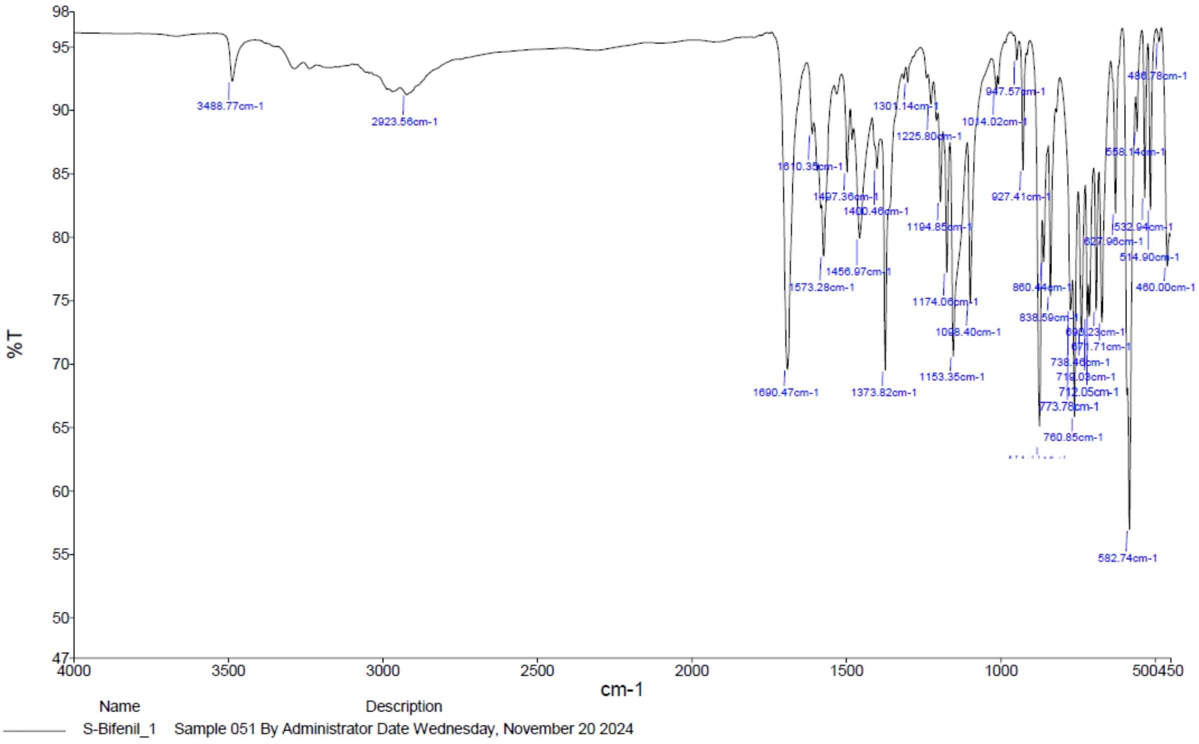

Figure S64. FT-IR spectrum of 16

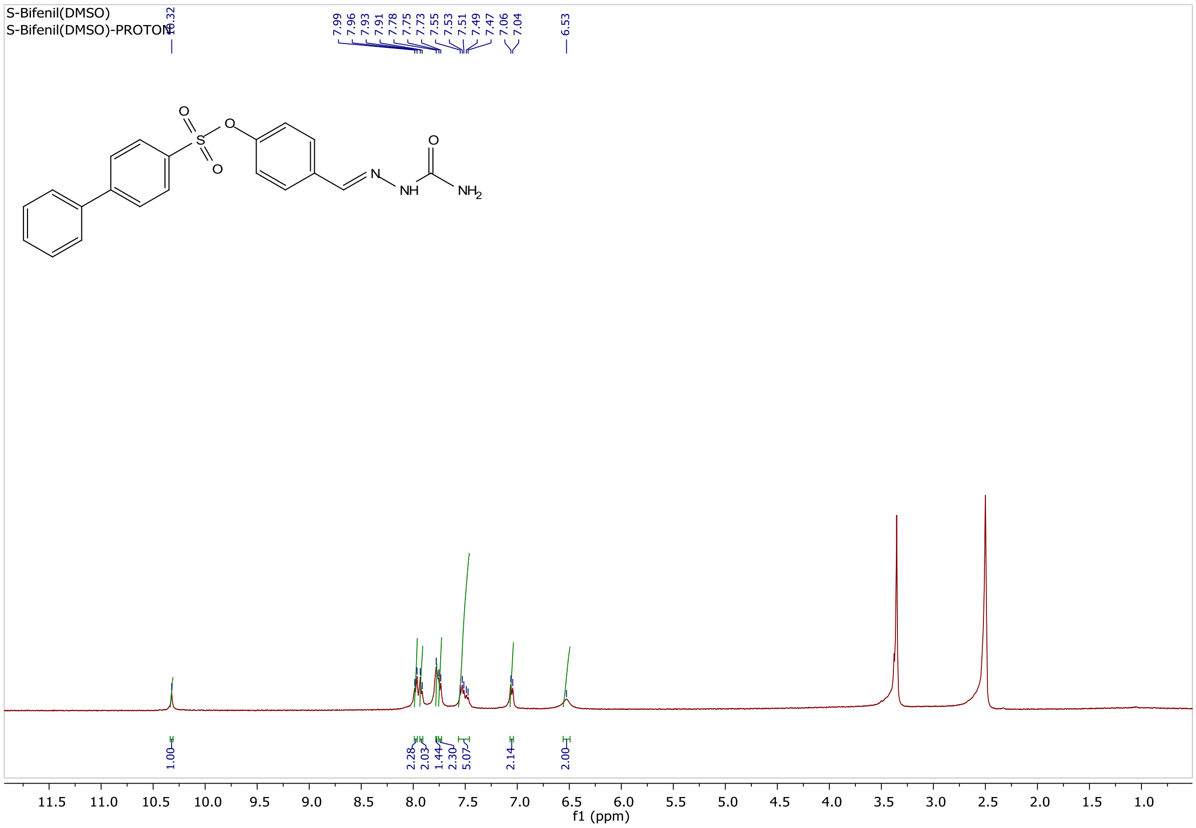

Figure S65. ^{1}H NMR spectrum of 16

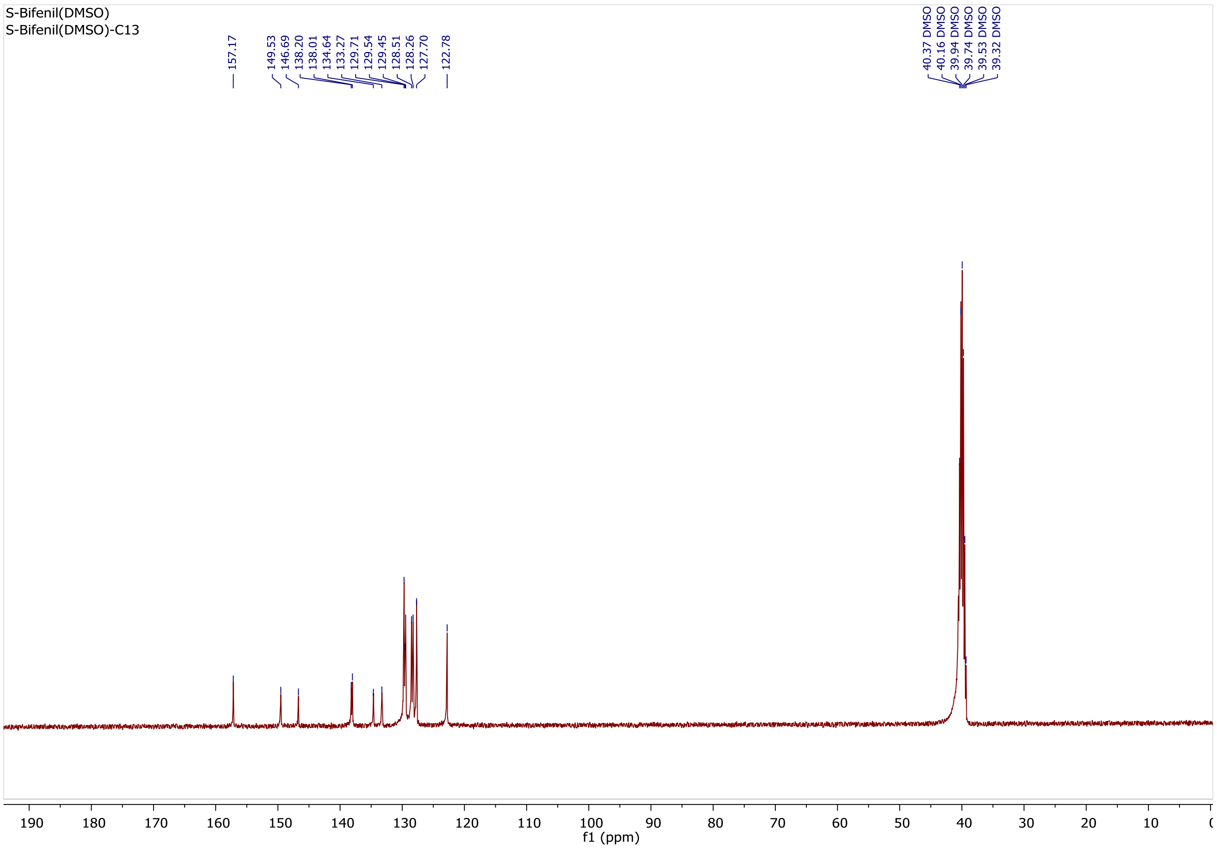

Figure S66. ^{13}C NMR spectrum of 16

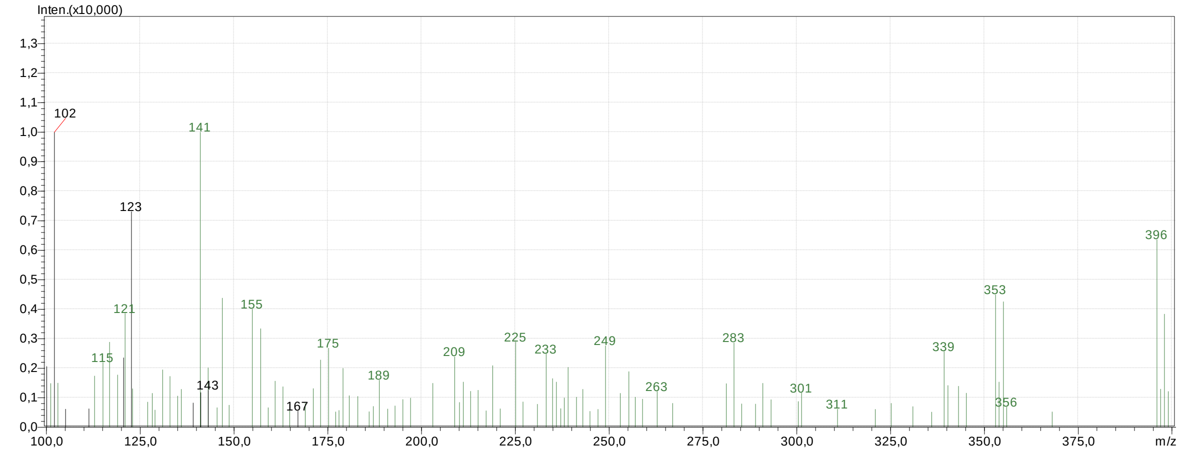

Figure S67. Mass spectrum of 16

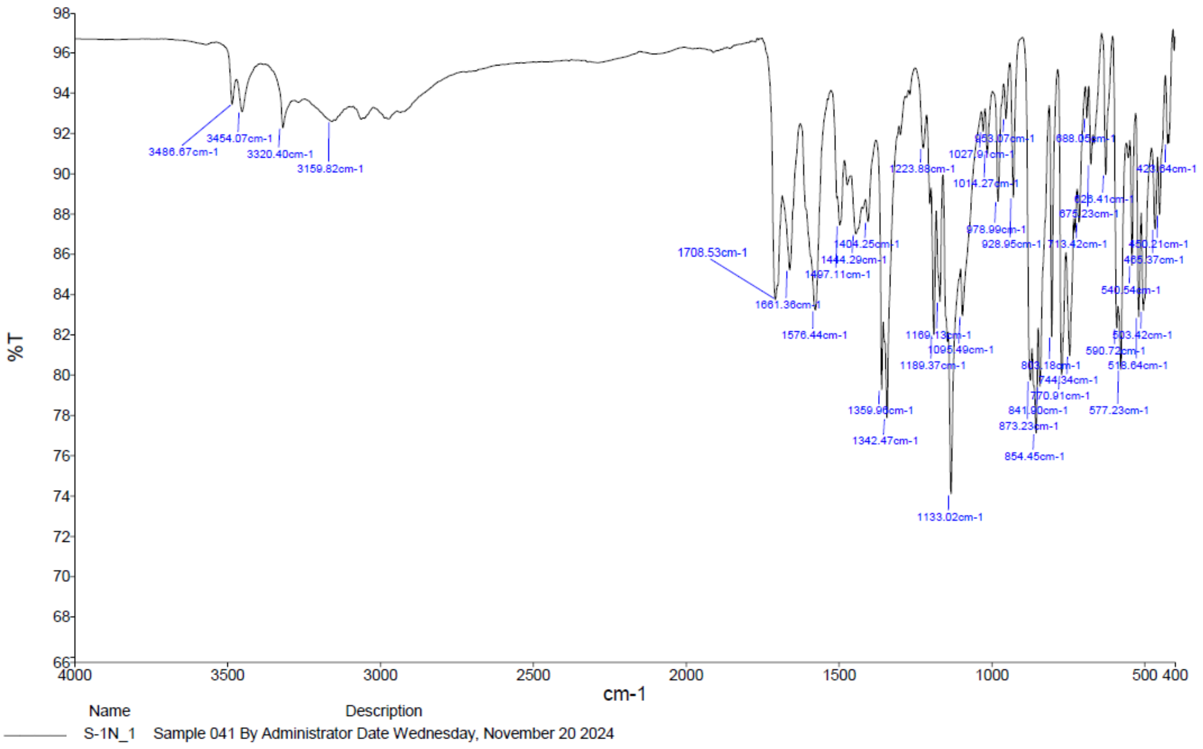

Figure S68. FT-IR spectrum of 17

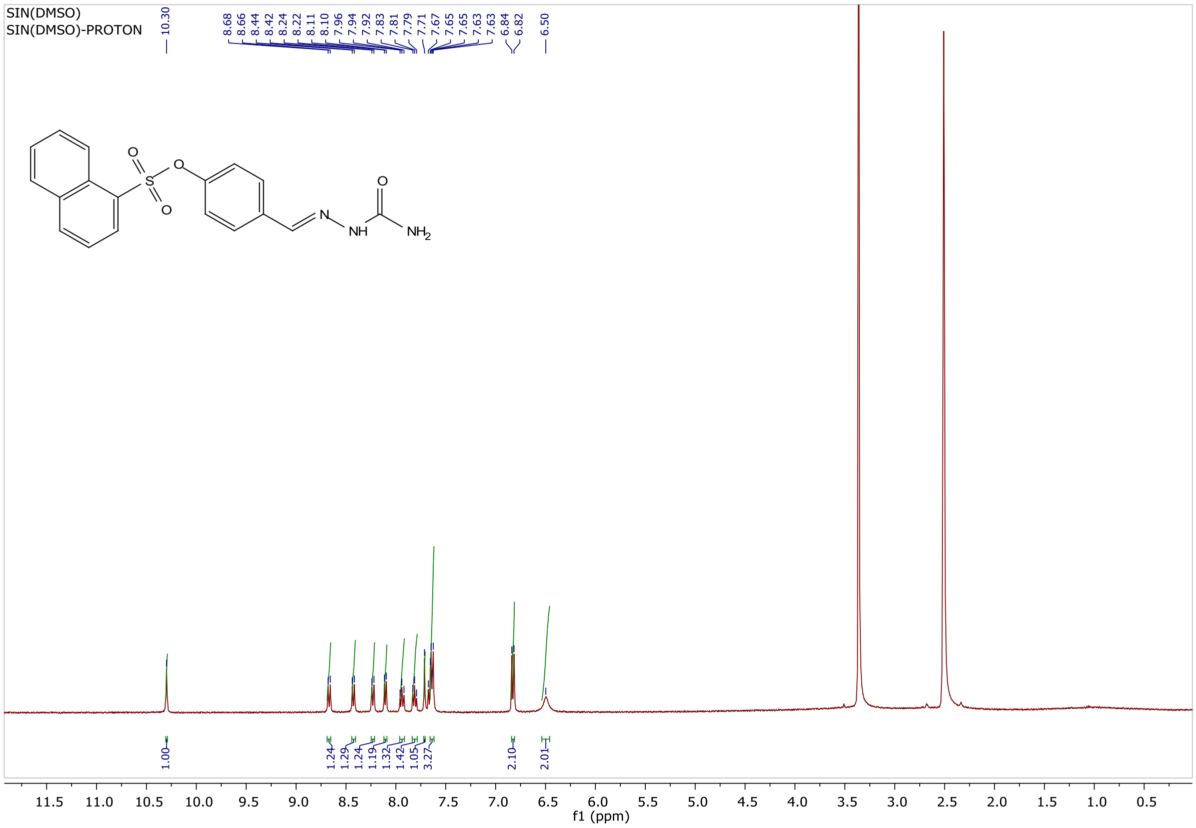

Figure S69. ^{1}H **NMR spectrum of 17**

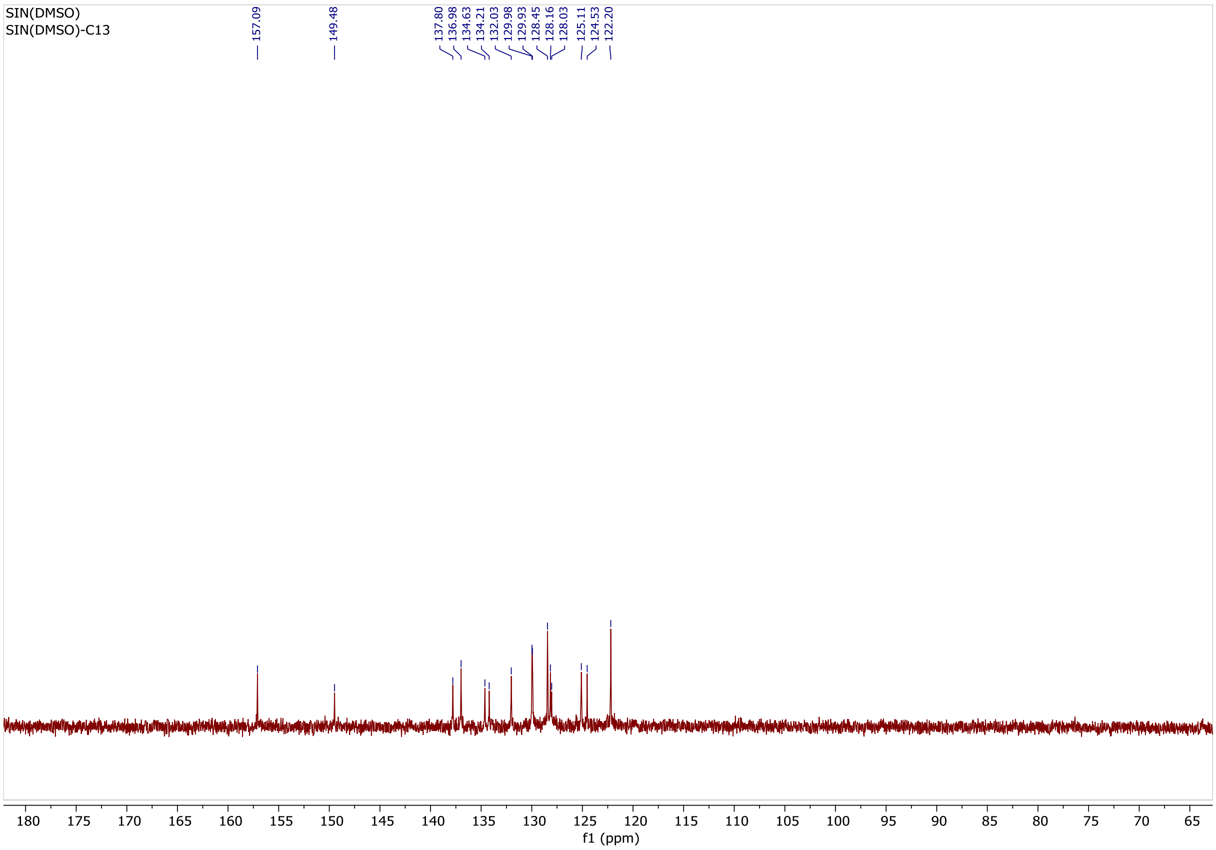

Figure S70. ^{13}C **NMR spectrum of 17**

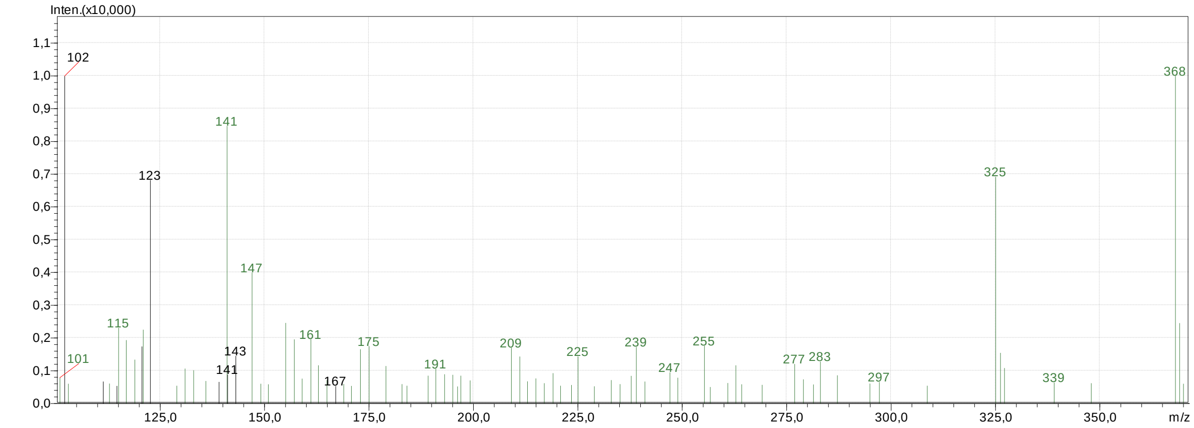

Figure S71. Mass spectrum of 17

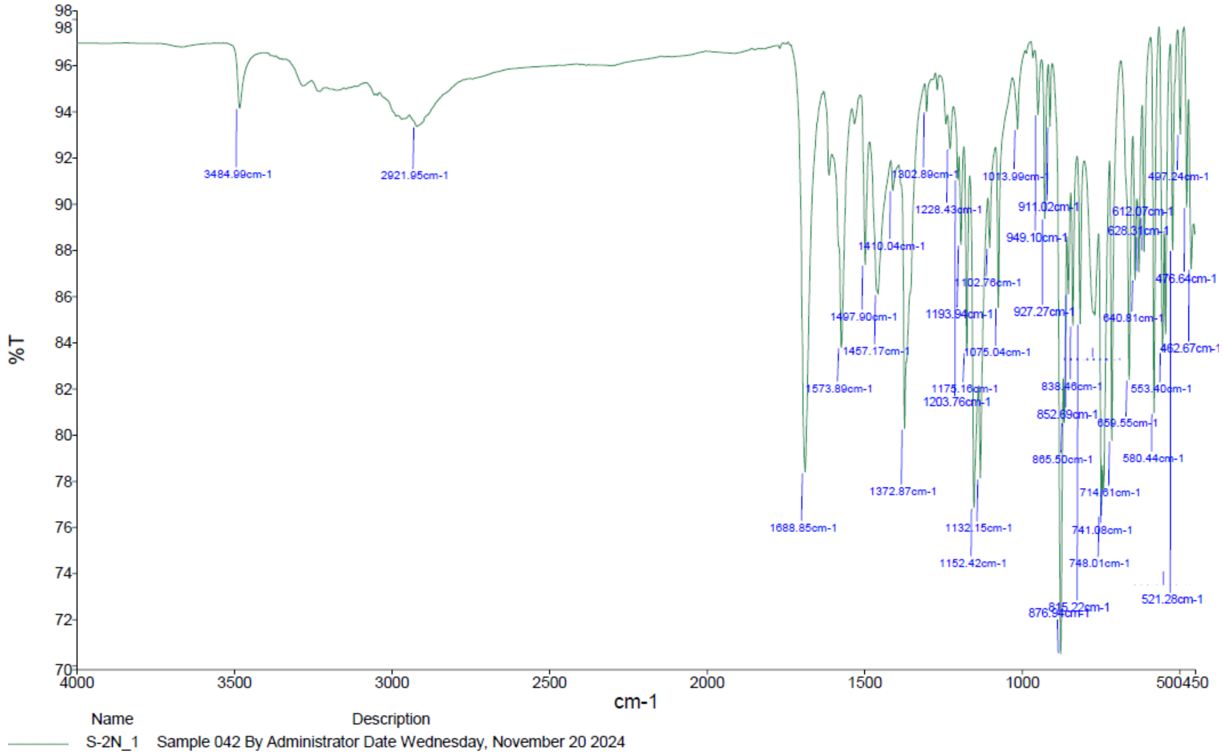

Figure S72. FT-IR spectrum of 18

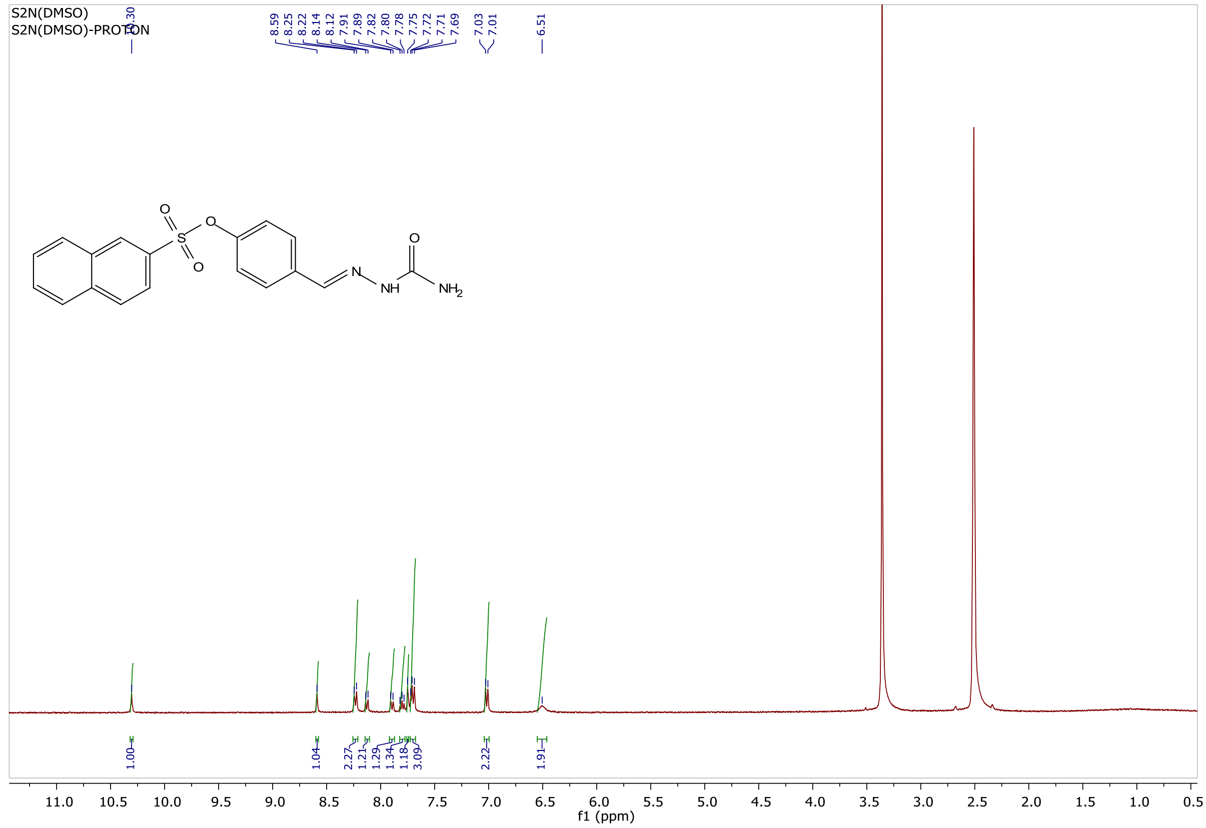

Figure S73. ^{1}H NMR spectrum of 18

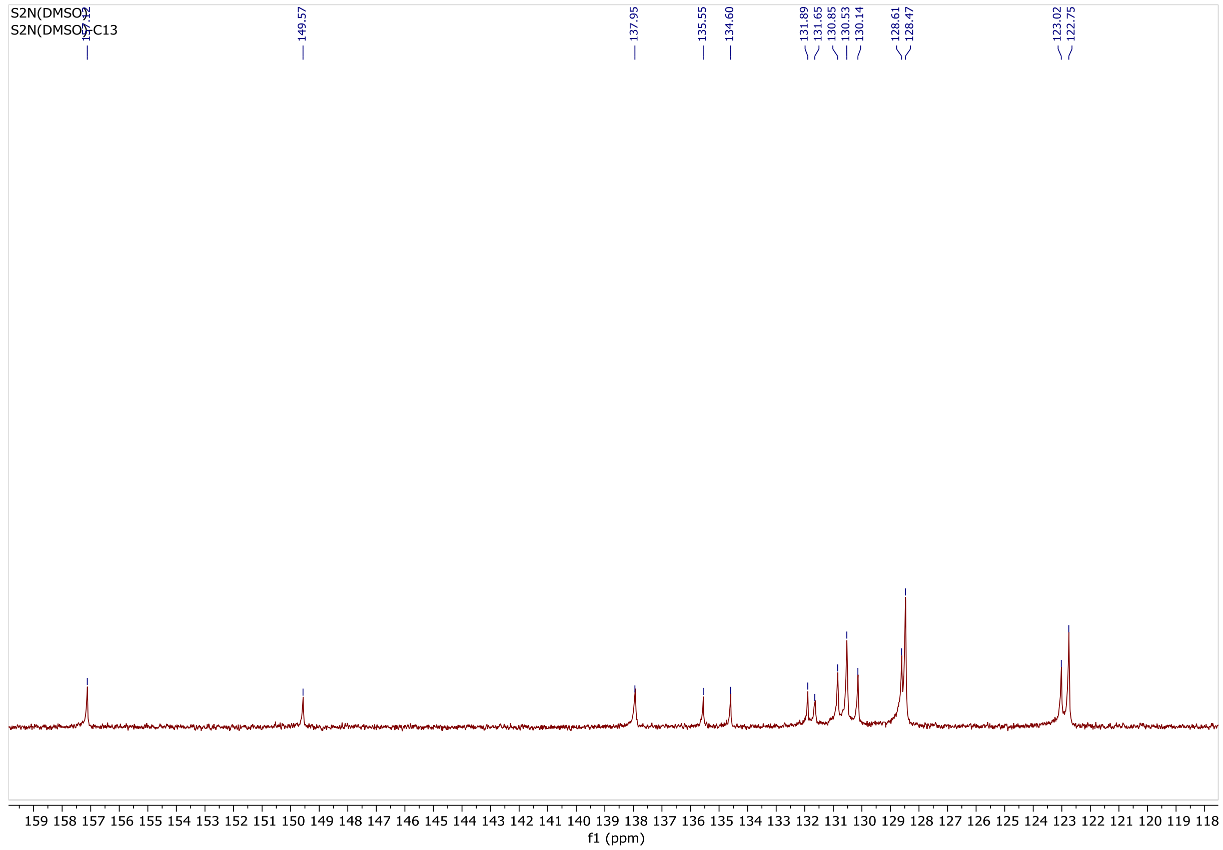

Figure S74. ^{13}C NMR spectrum of 18

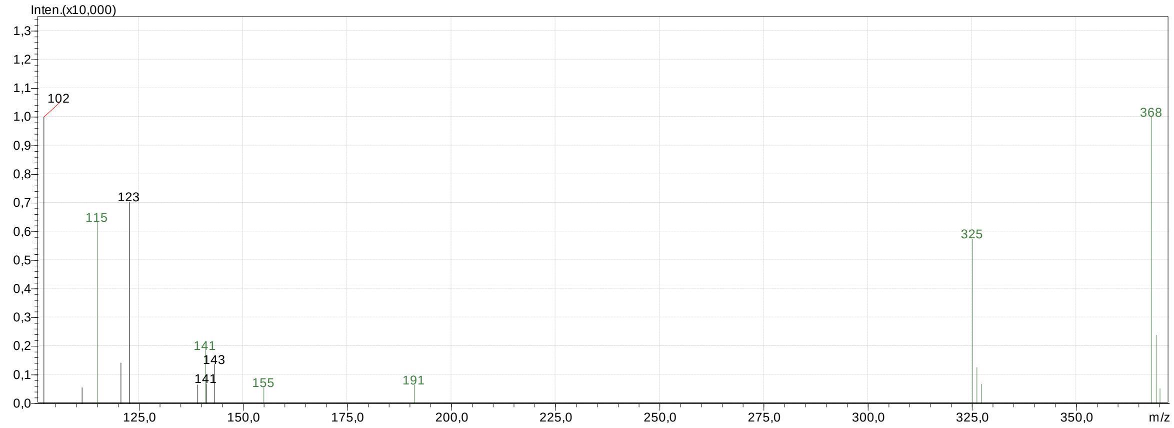

Figure S75. Mass spectrum of 18

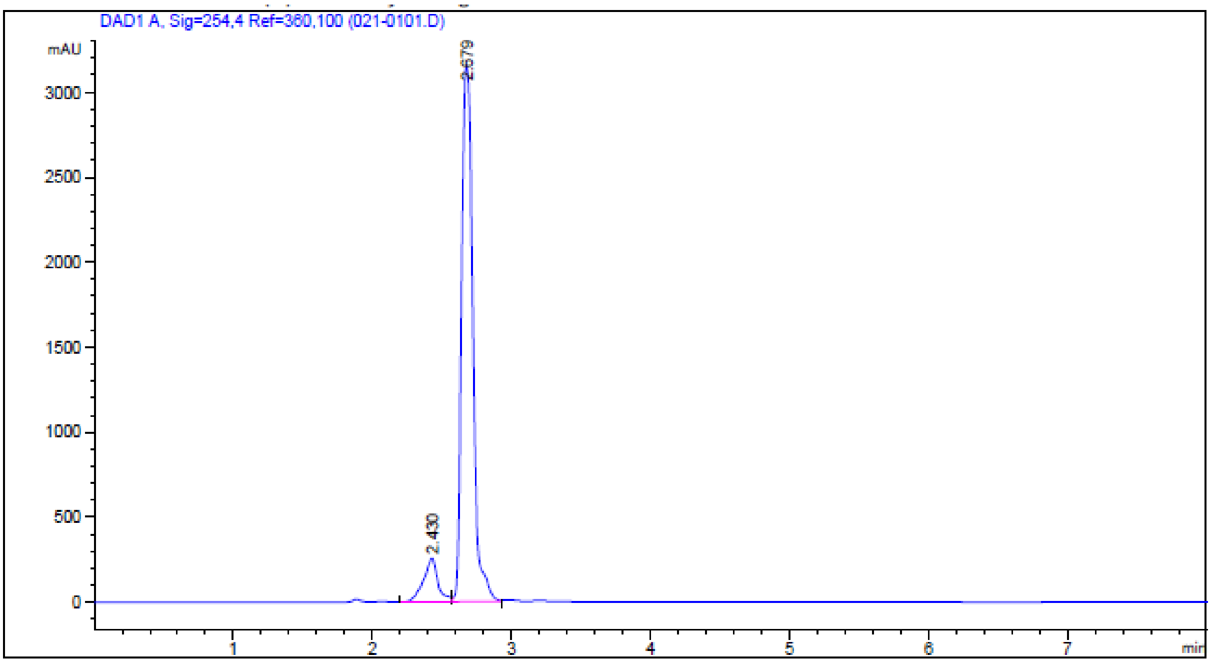

===

Area Percent Report

===

Sorted By : Signal
Multiplier : 1.0000
Dilution : 1.0000
Use Multiplier & Dilution Factor with ISTDs

Signal 1: DAD1 A, Sig=254,4 Ref=360,100

Peak #	RetTime [min]	Type	Width [min]	Area [mAU*s]	Height [mAU]	Area %
1	2.430	BV	0.1017	1827.63806	254.64804	9.3508
2	2.679	VV	0.0917	1.77176e4	3147.70605	90.6492

Figure S76. HPLC chromatogram of 1

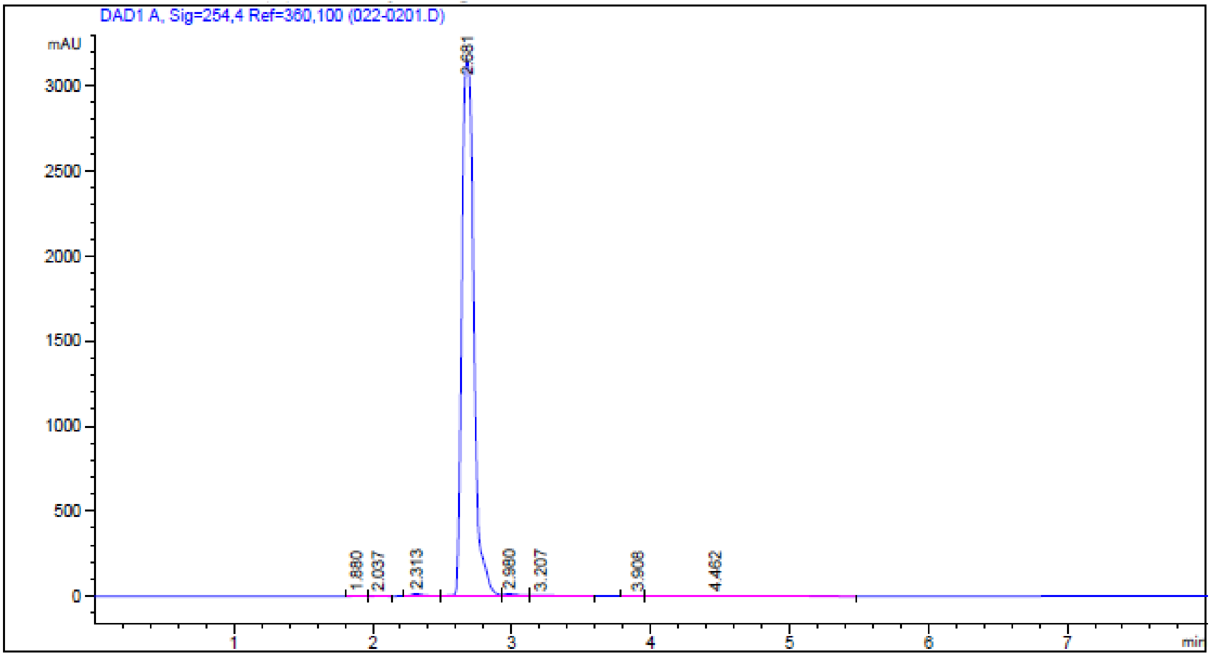

==

Area Percent Report

==

Sorted By : Signal
Multiplier : 1.0000
Dilution : 1.0000
Use Multiplier & Dilution Factor with ISTDs

Signal 1: DAD1 A, Sig=254,4 Ref=360,100

Peak #	RetTime [min]	Type	Width [min]	Area [mAU*s]	Height [mAU]	Area %
1	1.880	BV	0.0685	8.30190	1.84352	0.0435
2	2.037	VB	0.0792	4.29432	7.93329e-1	0.0225
3	2.313	BB	0.0815	58.59925	10.76149	0.3071
4	2.681	BV	0.0959	1.88506e4	3144.25122	98.7885
5	2.980	VV	0.0959	62.48814	9.12621	0.3275
6	3.207	VB	0.1416	46.83678	4.56835	0.2455
7	3.908	BV	0.0845	1.33908	2.28180e-1	7.018e-3
8	4.462	VB	0.5259	49.30828	1.15529	0.2584

Figure S77. HPLC chromatogram of 2

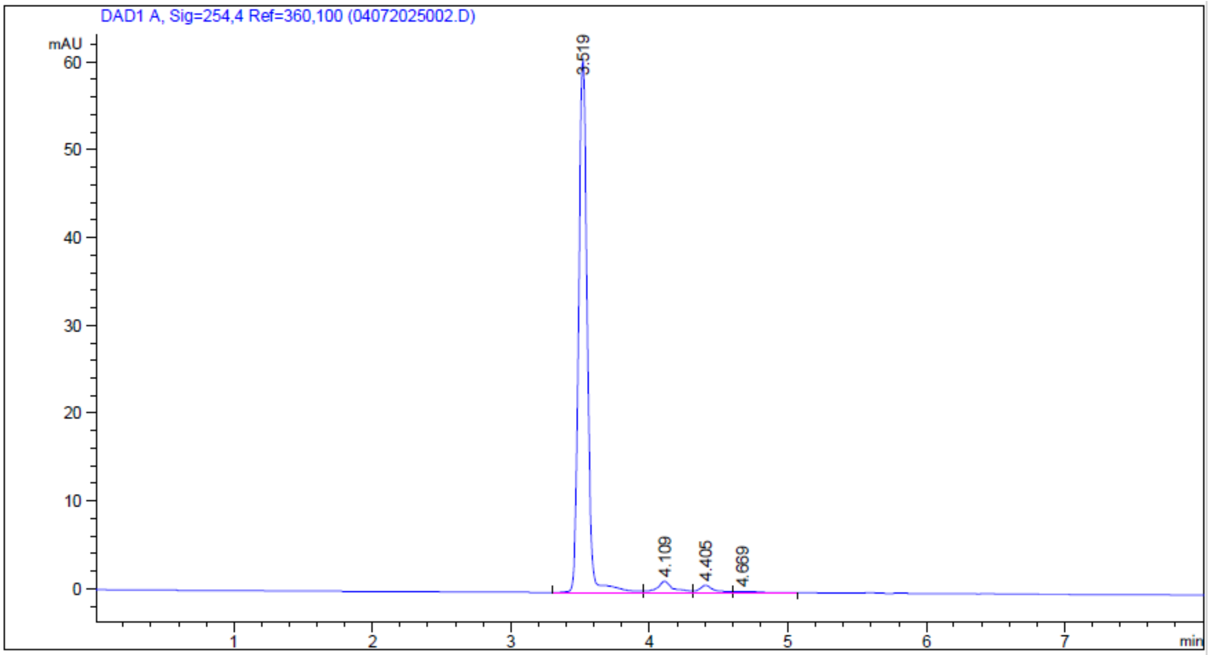


```
=====================================================================
                       Area Percent Report
=====================================================================

Sorted By             :      Signal
Multiplier            :      1.0000
Dilution              :      1.0000
Use Multiplier & Dilution Factor with ISTDs


Signal 1: DAD1 A, Sig=254,4 Ref=360,100

Peak RetTime Type  Width     Area      Height     Area
  #   [min]        [min]   [mAU*s]     [mAU]        %
----|-------|----|-------|----------|----------|--------|
   1   3.519 BV    0.0668  264.16531   60.69899  93.4783
   2   4.109 VV    0.1132   10.42288    1.27736   3.6883
   3   4.405 VV    0.1053    6.00653 8.21833e-1   2.1255
   4   4.669 VB    0.1680    2.00053 1.55618e-1   0.7079
```

Figure S78. HPLC chromatogram of 3

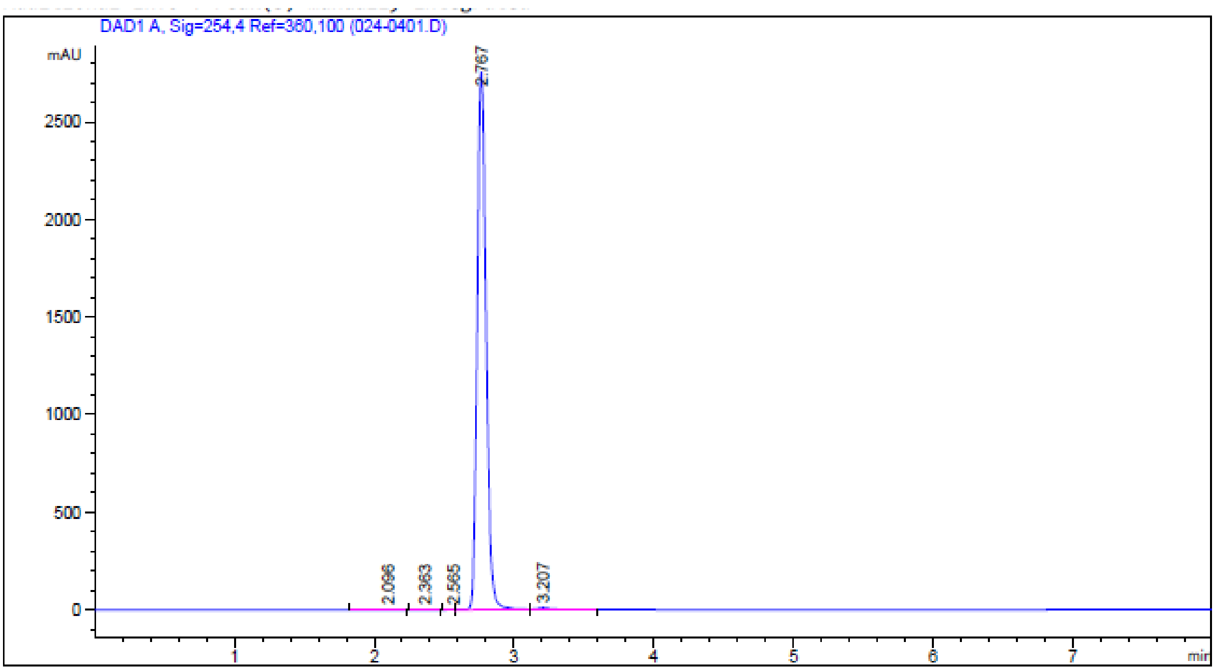


```
========================================================================
                          Area Percent Report
========================================================================

Sorted By             :      Signal
Multiplier            :      1.0000
Dilution              :      1.0000
Use Multiplier & Dilution Factor with ISTDs


Signal 1: DAD1 A, Sig=254,4 Ref=360,100
```

Peak #	RetTime [min]	Type	Width [min]	Area [mAU*s]	Height [mAU]	Area %
1	2.096	BB	0.1924	2.37800	1.70840e-1	0.0193
2	2.363	BB	0.0842	1.30326	2.29693e-1	0.0106
3	2.565	BV	0.0502	4.74903	1.44919	0.0385
4	2.767	VV	0.0677	1.22758e4	2768.62891	99.3949
5	3.207	VB	0.1186	66.30643	7.84347	0.5369

Figure S79. HPLC chromatogram of 4

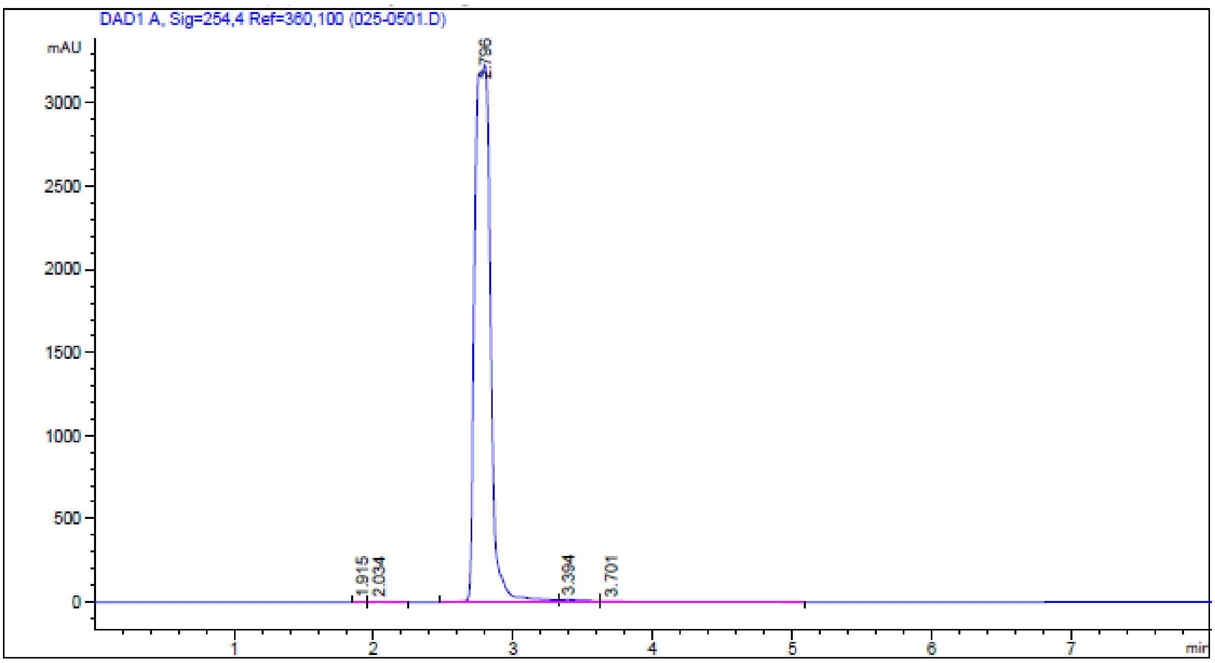

===

Area Percent Report

===

Sorted By : Signal
Multiplier : 1.0000
Dilution : 1.0000
Use Multiplier & Dilution Factor with ISTDs

Signal 1: DAD1 A, Sig=254,4 Ref=360,100

Peak #	RetTime [min]	Type	Width [min]	Area [mAU*s]	Height [mAU]	Area %
1	1.915	BV	0.0587	2.70797	6.76141e-1	0.0106
2	2.034	VB	0.1471	5.17130	4.67383e-1	0.0202
3	2.796	BV	0.1075	2.53324e4	3229.08740	98.8684
4	3.394	VV	0.1614	153.43217	12.65575	0.5988
5	3.701	VB	0.2816	128.62648	5.77986	0.5020

Figure S80. HPLC chromatogram of 5

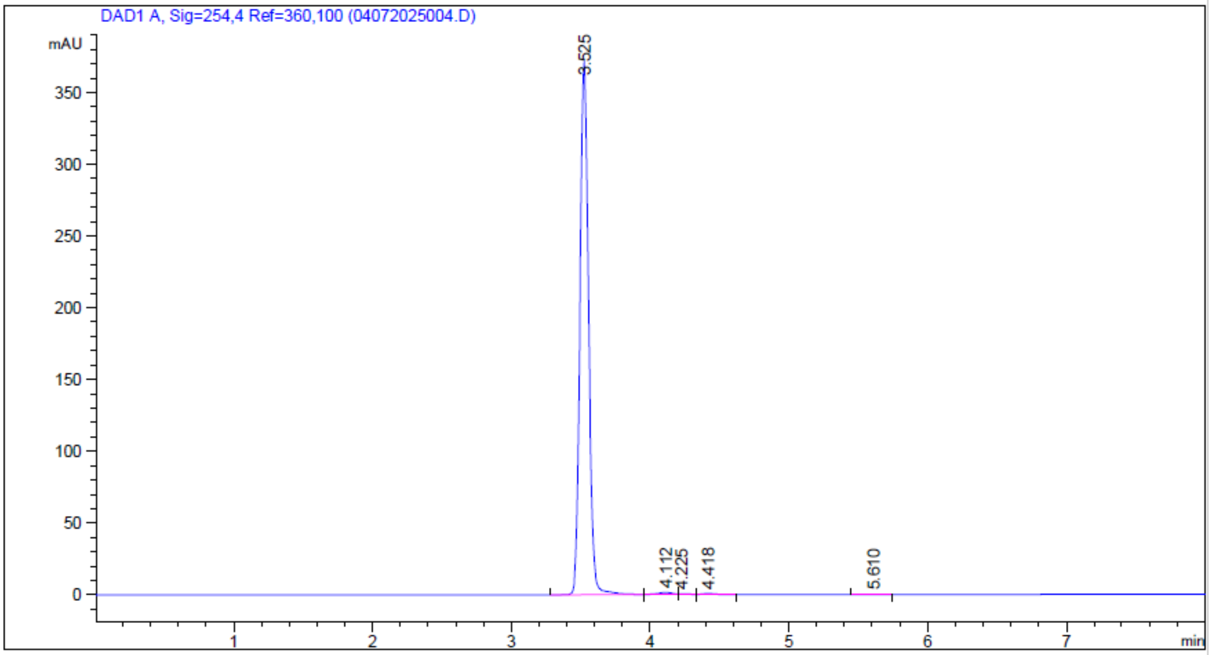


```
=====================================================================
                         Area Percent Report
=====================================================================

Sorted By             :      Signal
Multiplier            :      1.0000
Dilution              :      1.0000
Use Multiplier & Dilution Factor with ISTDs


Signal 1: DAD1 A, Sig=254,4 Ref=360,100
```

Peak #	RetTime [min]	Type	Width [min]	Area [mAU*s]	Height [mAU]	Area %
1	3.525	BV	0.0687	1615.51733	372.02390	98.7651
2	4.112	VV	0.1041	11.72179	1.62604	0.7166
3	4.225	VV	0.0828	2.77141	4.98804e-1	0.1694
4	4.418	VB	0.0978	4.69420	7.22409e-1	0.2870
5	5.610	BV	0.1029	1.01176	1.49605e-1	0.0619

Figure S81. HPLC chromatogram of 6

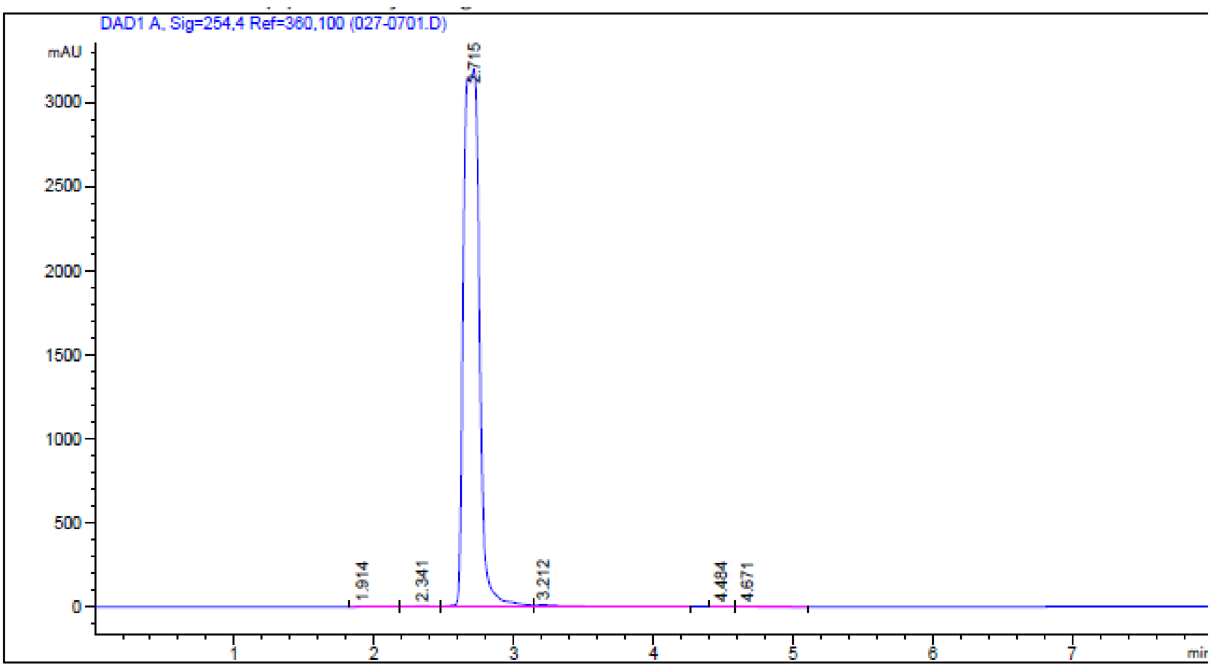


```
=====================================================================
                         Area Percent Report
=====================================================================

Sorted By             :      Signal
Multiplier            :      1.0000
Dilution              :      1.0000
Use Multiplier & Dilution Factor with ISTDs


Signal 1: DAD1 A, Sig=254,4 Ref=360,100
```

Peak #	RetTime [min]	Type	Width [min]	Area [mAU*s]	Height [mAU]	Area %
1	1.914	BB	0.1235	11.52816	1.25131	0.0455
2	2.341	BV	0.0924	35.20828	5.52274	0.1391
3	2.715	VV	0.1077	2.50908e4	3191.27881	99.1294
4	3.212	VB	0.2247	170.77834	9.73434	0.6747
5	4.484	BV	0.0998	8.40627e-1	1.26019e-1	3.321e-3
6	4.671	VB	0.1643	2.00547	1.66772e-1	7.923e-3

Figure S82. HPLC chromatogram of 7

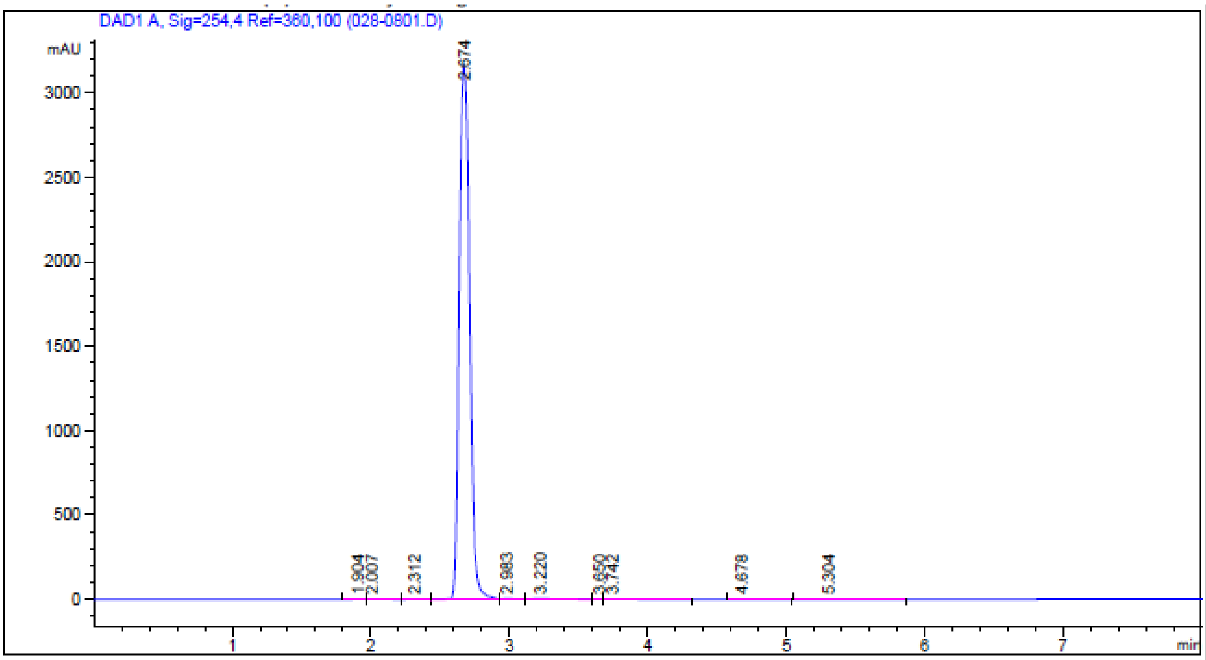

===

Area Percent Report

===

Sorted By : Signal
Multiplier : 1.0000
Dilution : 1.0000
Use Multiplier & Dilution Factor with ISTDs

Signal 1: DAD1 A, Sig=254,4 Ref=360,100

Peak #	RetTime [min]	Type	Width [min]	Area [mAU*s]	Height [mAU]	Area %
1	1.904	BV	0.0881	3.41256	5.84902e-1	0.0205
2	2.007	VV	0.1478	5.73045	4.99701e-1	0.0344
3	2.312	VV	0.1036	4.77019	6.65437e-1	0.0286
4	2.674	VB	0.0850	1.65409e4	3162.10034	99.2872
5	2.983	BV	0.0886	28.54747	4.58533	0.1714
6	3.220	VV	0.1703	59.69963	4.75928	0.3583
7	3.650	VV	0.0625	2.91719	6.73351e-1	0.0175
8	3.742	VB	0.0989	9.30611	1.34189	0.0559

Figure S83. HPLC chromatogram of 8

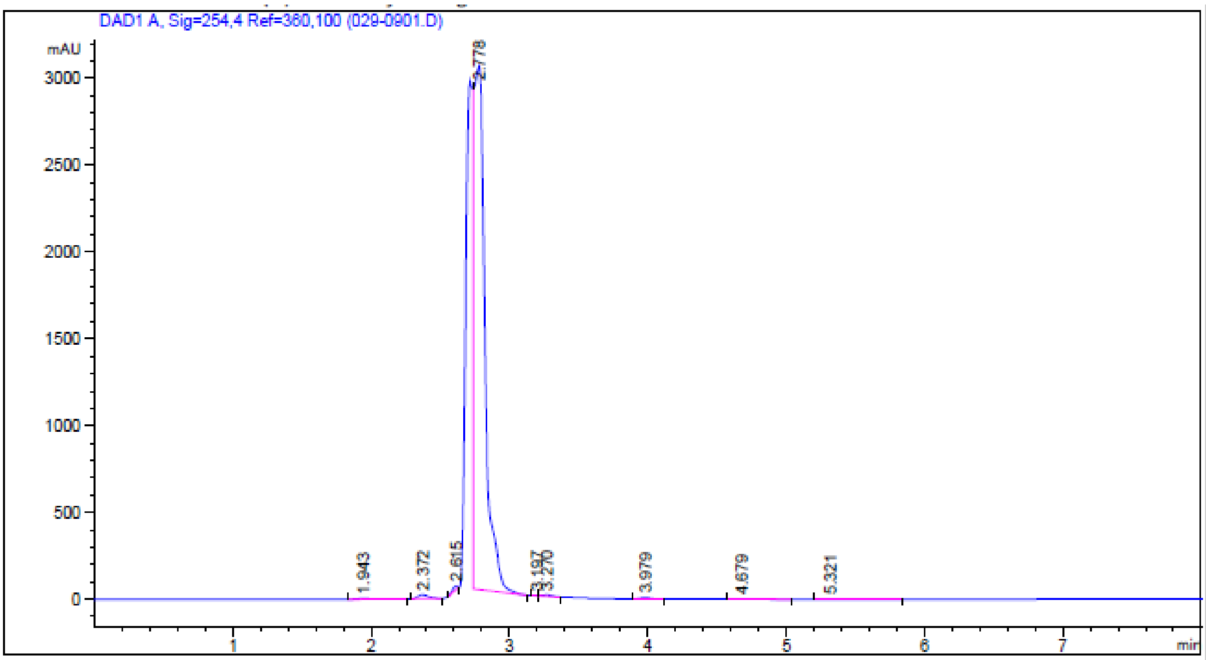

===

Area Percent Report

===

Sorted By : Signal
Multiplier : 1.0000
Dilution : 1.0000
Use Multiplier & Dilution Factor with ISTDs

Signal 1: DAD1 A, Sig=254,4 Ref=360,100

Peak #	RetTime [min]	Type	Width [min]	Area [mAU*s]	Height [mAU]	Area %
1	1.943	BB	0.1097	33.63194	4.18951	0.1921
2	2.372	BB	0.0729	112.72567	23.12867	0.6438
3	2.615	BB	0.0462	58.71506	20.02141	0.3353
4	2.778	VB	0.0826	1.72254e4	3017.23975	98.3735
5	3.197	BV	0.0369	5.51244	2.22217	0.0315
6	3.270	VB	0.0718	38.36035	8.02321	0.2191
7	3.979	BB	0.0734	32.15339	6.77605	0.1836
8	4.679	BB	0.1239	1.90846	2.27424e-1	0.0109

Figure S84. HPLC chromatogram of 9

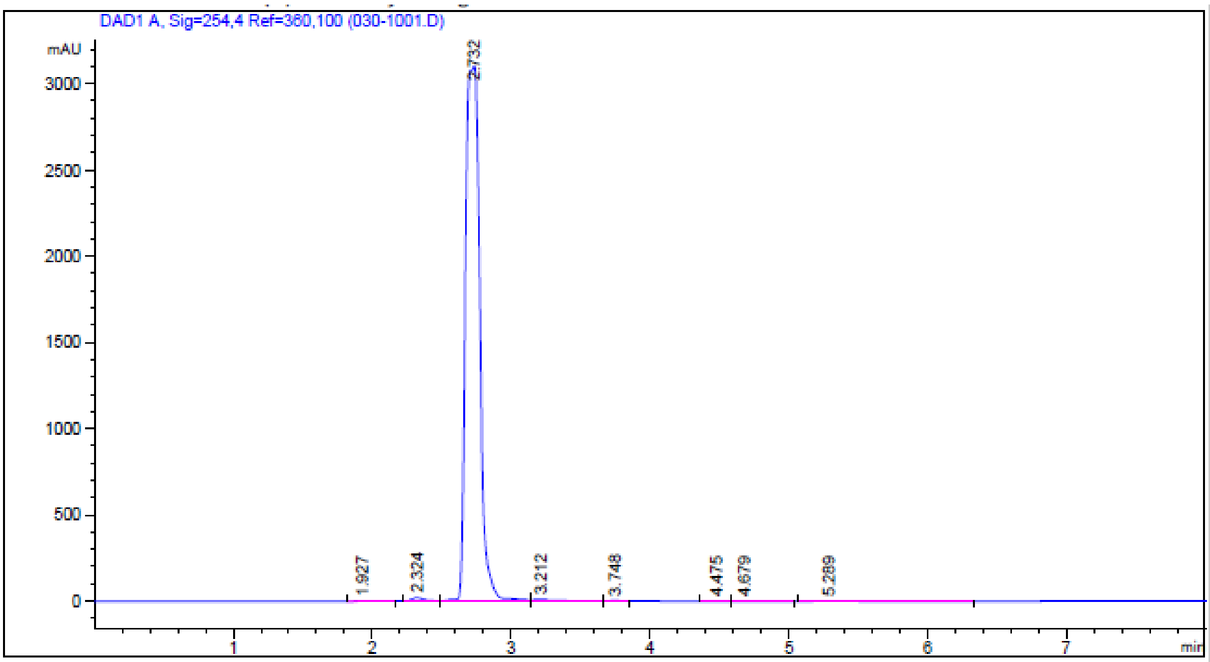

===

Area Percent Report

===

Sorted By : Signal
Multiplier : 1.0000
Dilution : 1.0000
Use Multiplier & Dilution Factor with ISTDs

Signal 1: DAD1 A, Sig=254,4 Ref=360,100

Peak #	RetTime [min]	Type	Width [min]	Area [mAU*s]	Height [mAU]	Area %
1	1.927	BB	0.1375	8.81901	9.06749e-1	0.0396
2	2.324	BV	0.0831	108.46436	18.87231	0.4875
3	2.732	VV	0.1149	2.20158e4	3097.13013	98.9477
4	3.212	VB	0.1652	78.75550	6.50703	0.3540
5	3.748	BB	0.0686	13.31149	3.06903	0.0598
6	4.475	BV	0.1125	7.14992e-1	9.42447e-2	3.213e-3
7	4.679	VB	0.1632	1.88745	1.62892e-1	8.483e-3
8	5.289	BB	0.3034	22.17375	1.06294	0.0997

Figure S85. HPLC chromatogram of 10

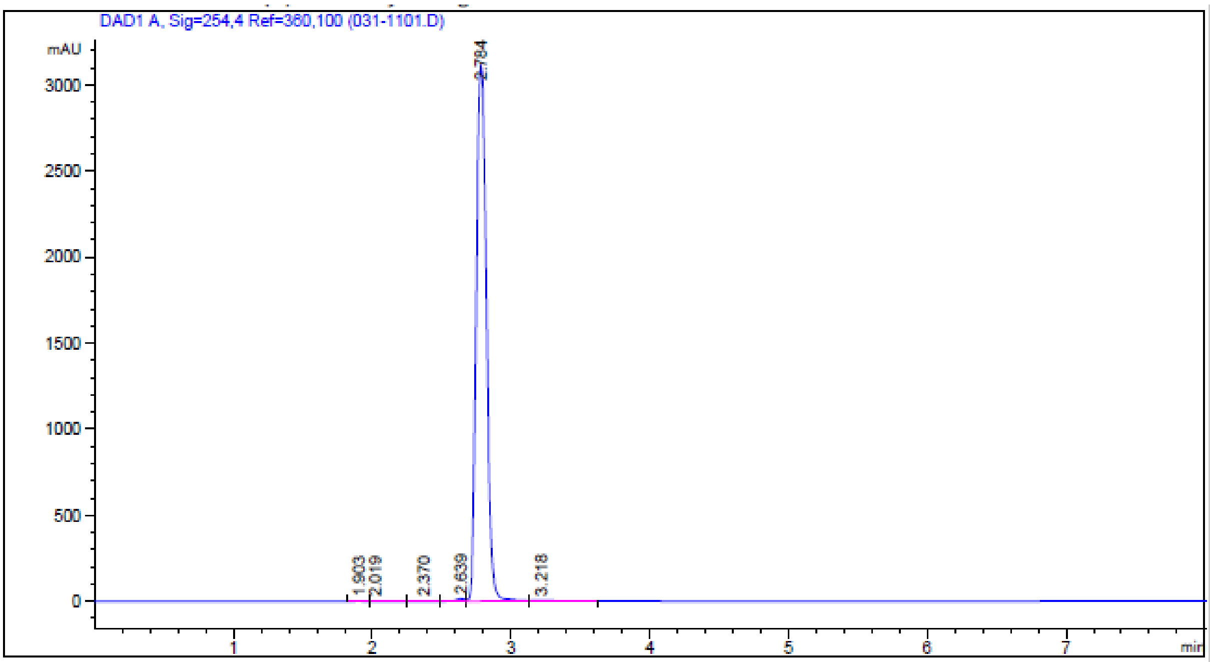


```
=====================================================================
                        Area Percent Report
=====================================================================

Sorted By             :      Signal
Multiplier            :      1.0000
Dilution              :      1.0000
Use Multiplier & Dilution Factor with ISTDs


Signal 1: DAD1 A, Sig=254,4 Ref=360,100
```

Peak #	RetTime [min]	Type	Width [min]	Area [mAU*s]	Height [mAU]	Area %
1	1.903	BV	0.0606	3.16859	7.92979e-1	0.0198
2	2.019	VB	0.1259	1.70878	1.74952e-1	0.0107
3	2.370	BB	0.0765	1.22900	2.45031e-1	7.694e-3
4	2.639	BV	0.0661	53.57443	12.48078	0.3354
5	2.784	VV	0.0834	1.58554e4	3112.51392	99.2592
6	3.218	VB	0.1557	58.64869	5.19431	0.3672

Figure S86. HPLC chromatogram of 11

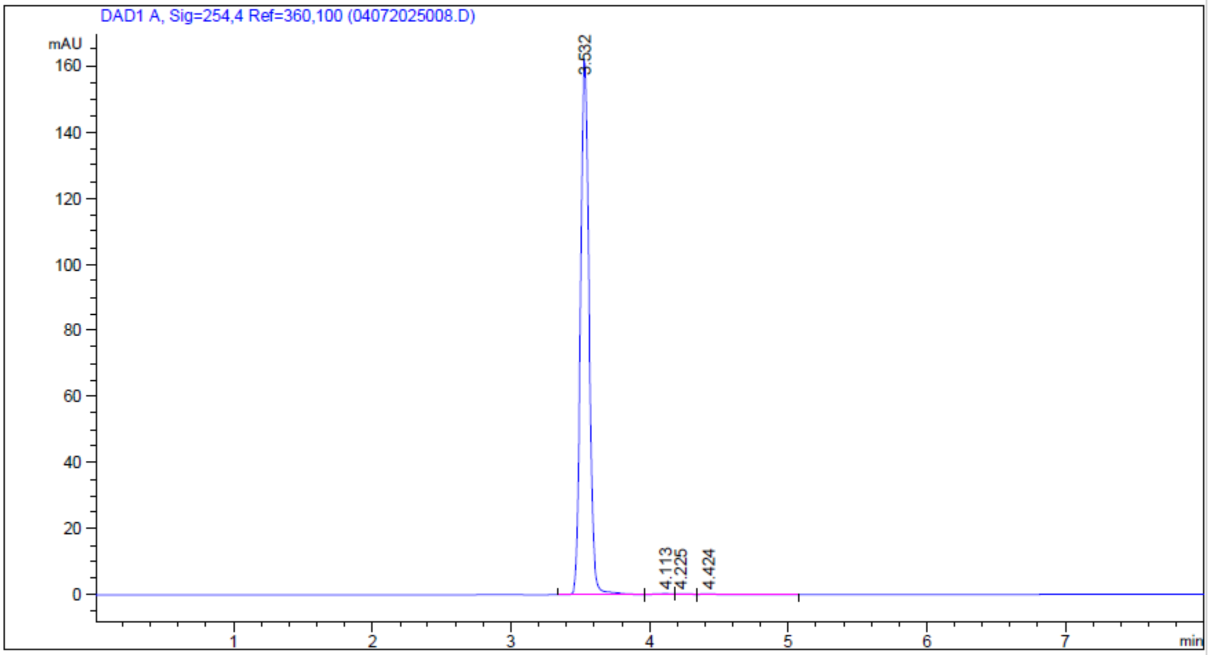

==

Area Percent Report

==

Sorted By : Signal
Multiplier : 1.0000
Dilution : 1.0000
Use Multiplier & Dilution Factor with ISTDs

Signal 1: DAD1 A, Sig=254,4 Ref=360,100

Peak #	RetTime [min]	Type	Width [min]	Area [mAU*s]	Height [mAU]	Area %
1	3.532	BV	0.0663	698.45612	162.07529	99.0496
2	4.113	VV	0.1384	2.66614	2.86917e-1	0.3781
3	4.225	VV	0.1021	1.62749	2.25737e-1	0.2308
4	4.424	VB	0.1507	2.40825	2.14985e-1	0.3415

Figure S87. HPLC chromatogram of 12

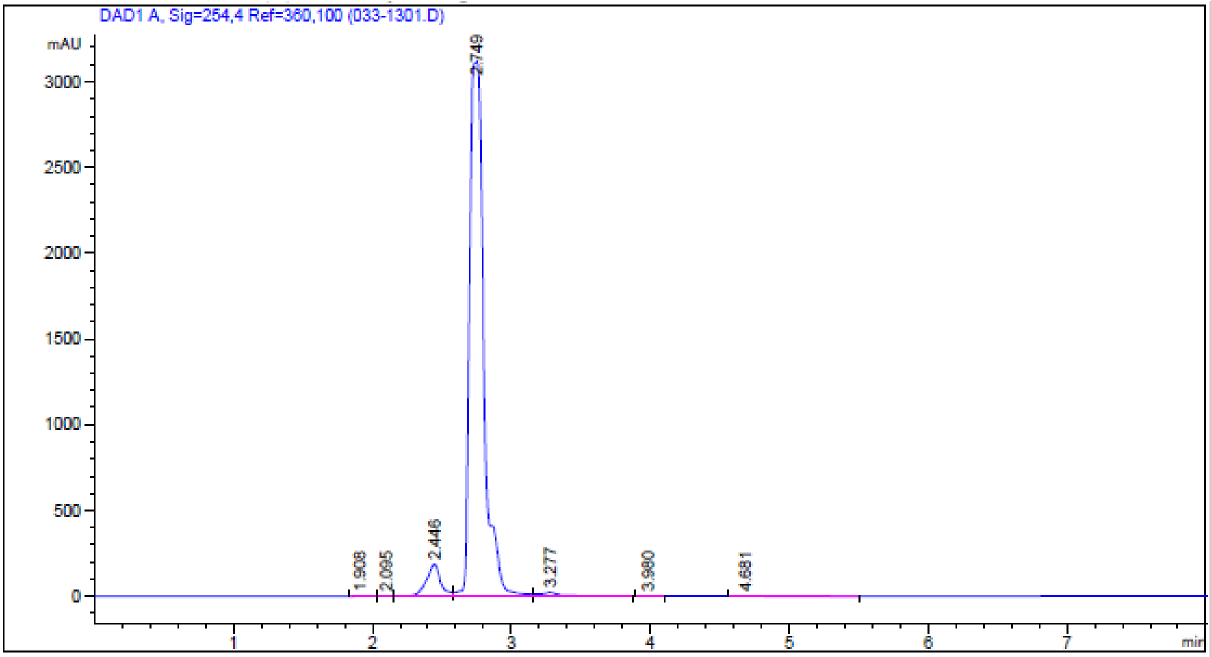


```
=====================================================================
                        Area Percent Report
=====================================================================

Sorted By             :      Signal
Multiplier            :      1.0000
Dilution              :      1.0000
Use Multiplier & Dilution Factor with ISTDs


Signal 1: DAD1 A, Sig=254,4 Ref=360,100
```

Peak #	RetTime [min]	Type	Width [min]	Area [mAU*s]	Height [mAU]	Area %
1	1.908	BV	0.0946	15.59216	2.37564	0.0662
2	2.095	VB	0.0713	3.94458	8.03166e-1	0.0167
3	2.446	BV	0.0978	1263.60730	184.70390	5.3657
4	2.749	VV	0.1127	2.20701e4	3113.74829	93.7168
5	3.277	VB	0.1281	187.02071	19.81406	0.7942
6	3.980	BB	0.0735	6.77935	1.42394	0.0288
7	4.681	BB	0.2282	2.73849	1.58021e-1	0.0116

Figure S88. HPLC chromatogram of 13

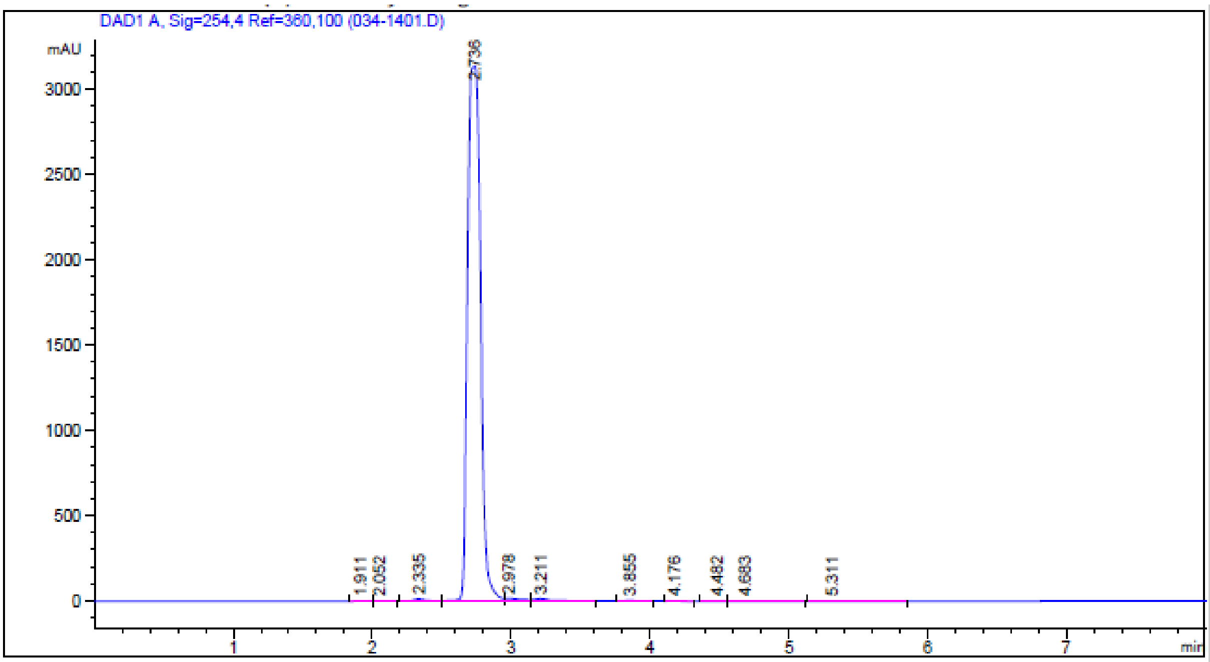

Area Percent Report

Sorted By : Signal
Multiplier : 1.0000
Dilution : 1.0000
Use Multiplier & Dilution Factor with ISTDs

Signal 1: DAD1 A, Sig=254,4 Ref=360,100

Peak #	RetTime [min]	Type	Width [min]	Area [mAU*s]	Height [mAU]	Area %
1	1.911	BV	0.0778	10.32722	1.88810	0.0502
2	2.052	VB	0.0849	4.71139	7.53667e-1	0.0229
3	2.335	BB	0.0797	46.36713	8.76292	0.2252
4	2.736	BV	0.1057	2.03140e4	3130.90381	98.6794
5	2.978	VV	0.1004	96.18201	13.28784	0.4672
6	3.211	VB	0.1174	91.52063	10.74505	0.4446
7	3.855	BB	0.0764	16.97853	3.51495	0.0825
8	4.176	BB	0.0763	9.62699e-1	1.99619e-1	4.677e-3

Figure S89. HPLC chromatogram of 14

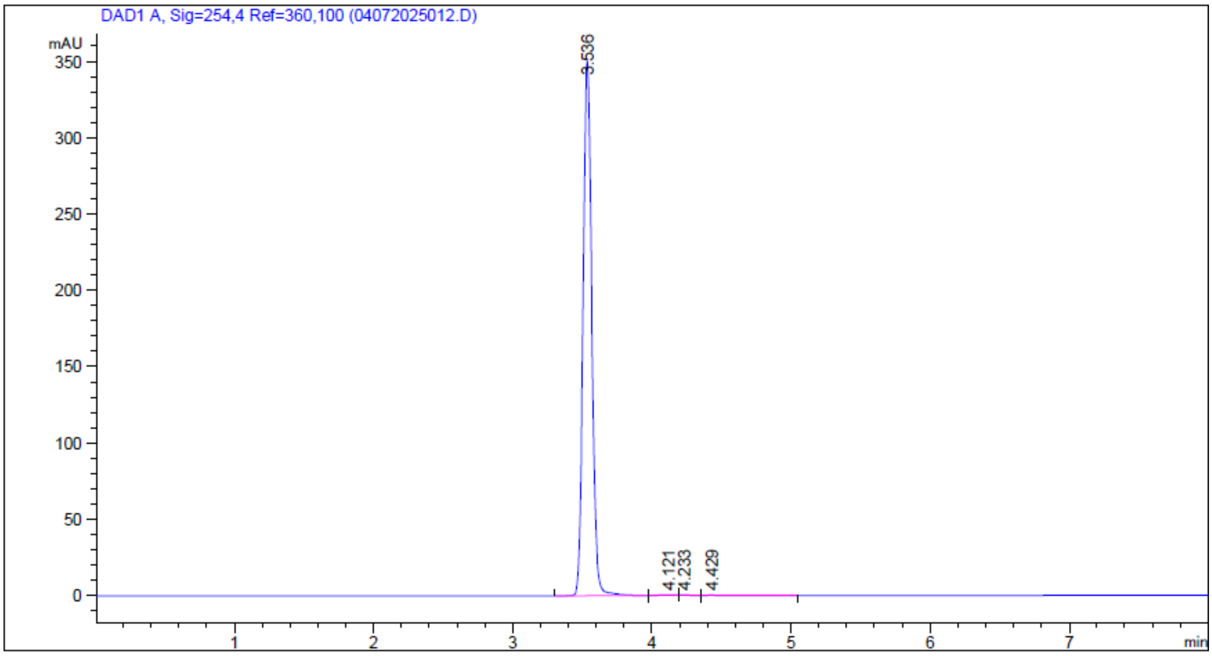

===

Area Percent Report

===

Sorted By : Signal
Multiplier : 1.0000
Dilution : 1.0000
Use Multiplier & Dilution Factor with ISTDs

Signal 1: DAD1 A, Sig=254,4 Ref=360,100

Peak #	RetTime [min]	Type	Width [min]	Area [mAU*s]	Height [mAU]	Area %
1	3.536	BV	0.0687	1524.82935	350.91873	99.2661
2	4.121	VV	0.1434	4.90933	5.05052e-1	0.3196
3	4.233	VV	0.1018	2.60107	3.71121e-1	0.1693
4	4.429	VB	0.1482	3.76244	3.47845e-1	0.2449

Figure S90. HPLC chromatogram of 15

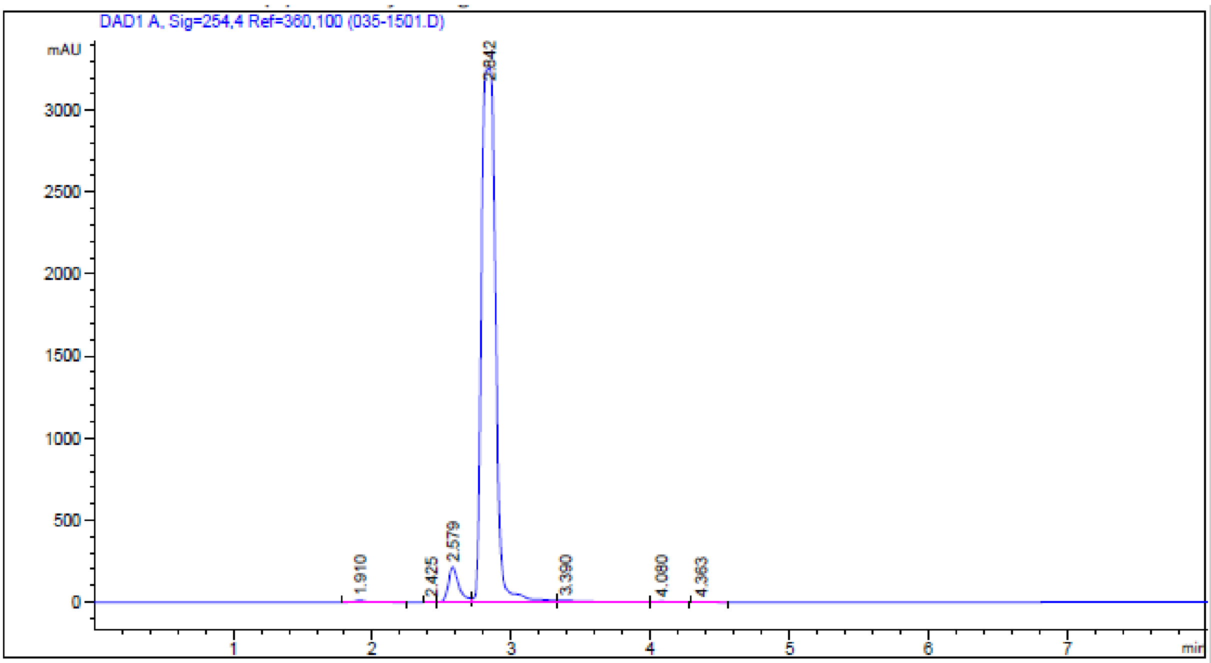

===

Area Percent Report

===

Sorted By : Signal
Multiplier : 1.0000
Dilution : 1.0000
Use Multiplier & Dilution Factor with ISTDs

Signal 1: DAD1 A, Sig=254,4 Ref=360,100

Peak #	RetTime [min]	Type	Width [min]	Area [mAU*s]	Height [mAU]	Area %
1	1.910	BB	0.0821	50.63764	8.67978	0.2107
2	2.425	BB	0.0523	1.30023	3.96433e-1	5.411e-3
3	2.579	BV	0.0792	1110.60986	211.71632	4.6218
4	2.842	VV	0.0974	2.27561e4	3262.45654	94.7001
5	3.390	VB	0.1737	96.96619	7.45956	0.4035
6	4.080	BB	0.0731	12.42125	2.72860	0.0517
7	4.363	BB	0.0767	1.62296	3.22375e-1	6.754e-3

Figure S91. HPLC chromatogram of 16

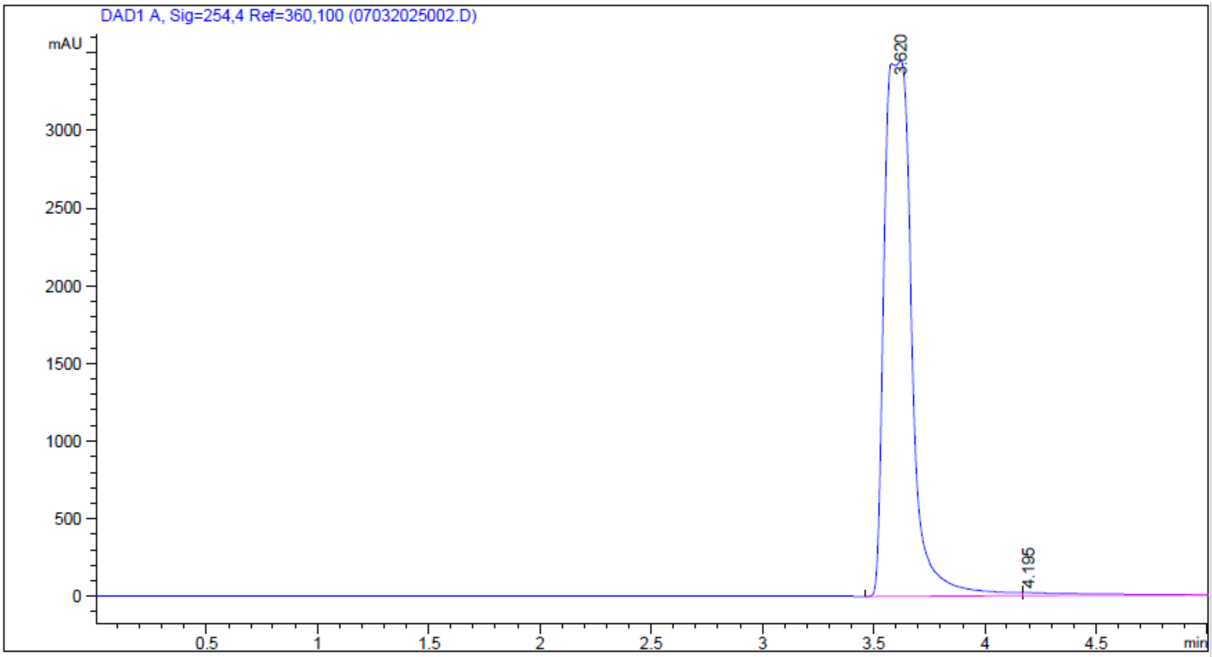

===

Area Percent Report

===

Sorted By : Signal
Multiplier : 1.0000
Dilution : 1.0000
Use Multiplier & Dilution Factor with ISTDs

Signal 1: DAD1 A, Sig=254,4 Ref=360,100

Peak #	RetTime [min]	Type	Width [min]	Area [mAU*s]	Height [mAU]	Area %
1	3.620	BV	0.1166	2.97518e4	3451.46313	98.7125
2	4.195	VBA	0.2551	388.06250	18.93274	1.2875

Figure S92. HPLC chromatogram of 17

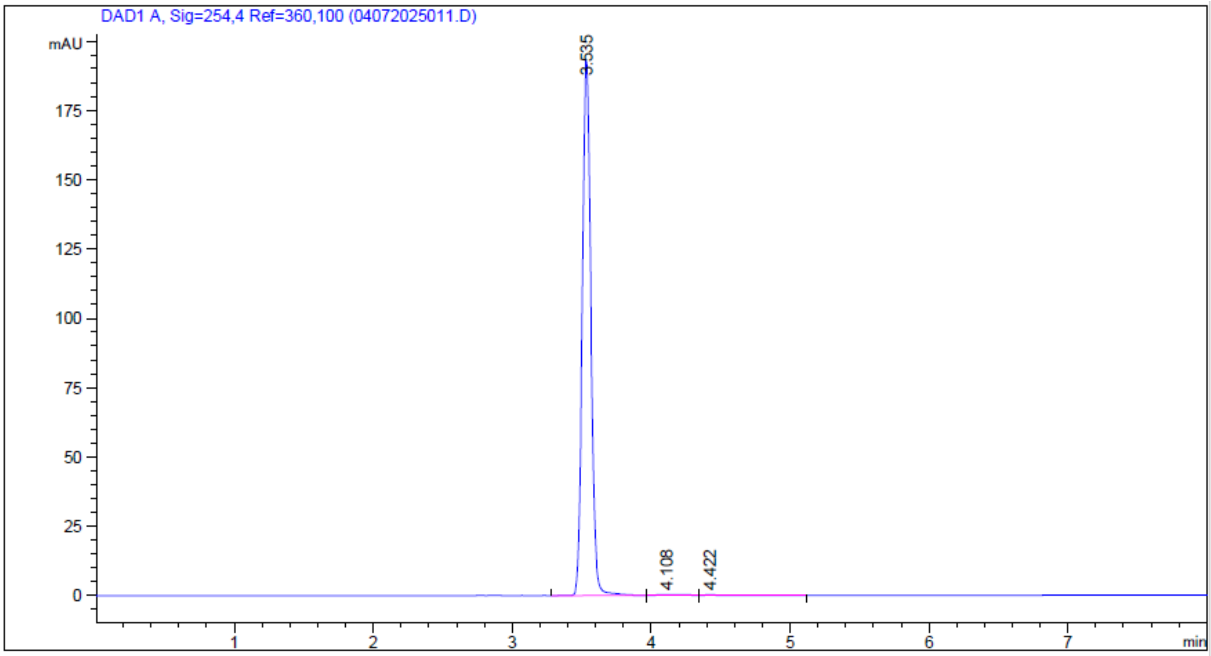

===
Area Percent Report
===

Sorted By : Signal
Multiplier : 1.0000
Dilution : 1.0000
Use Multiplier & Dilution Factor with ISTDs

Signal 1: DAD1 A, Sig=254,4 Ref=360,100

Peak #	RetTime [min]	Type	Width [min]	Area [mAU*s]	Height [mAU]	Area %
1	3.535	BV	0.0664	837.46320	193.78322	99.1627
2	4.108	VV	0.2122	4.49099	2.90721e-1	0.5318
3	4.422	VB	0.1550	2.58022	2.19781e-1	0.3055

Figure S93. HPLC chromatogram of 18
